# Association between Long-Term Exposure to Traffic-Related Air Pollution and Cardio-Metabolic Phenotypes: An MRI Data-Based Analysis

**DOI:** 10.1021/acs.est.4c03163

**Published:** 2024-10-04

**Authors:** Margarethe Woeckel, Susanne Rospleszcz, Kathrin Wolf, Susanne Breitner-Busch, Michael Ingrisch, Fabian Bamberg, Jens Ricke, Christopher L Schlett, Corinna Storz, Alexandra Schneider, Sophia Stoecklein, Annette Peters

**Affiliations:** † Institute of Epidemiology, German Research Center for Environmental Health, Helmholtz Zentrum München, Neuherberg 85764, Germany; ‡ Chair of Epidemiology, Institute for Medical Information Processing, Biometry and Epidemiology, Medical Faculty, Ludwig-Maximilians-Universität München (LMU Munich), Munich 81377, Germany; § Department of Diagnostic and Interventional Radiology, Medical Center, Faculty of Medicine, University of Freiburg, Freiburg 79106, Germany; ∥ Department of Radiology, Ludwig-Maximilians-Universität Hospital Munich, Munich 81377, Germany; ⊥ Department of Neuroradiology, Medical Center, University of Freiburg, Freiburg 79106, Germany; # German Center for Cardiovascular Disease Research (DZHK), Munich Heart Alliance, Munich 80336, Germany

**Keywords:** cardiometabolic disease, traffic-related air pollution, ultrafine particles, magnetic resonance imaging, cross-sectional study

## Abstract

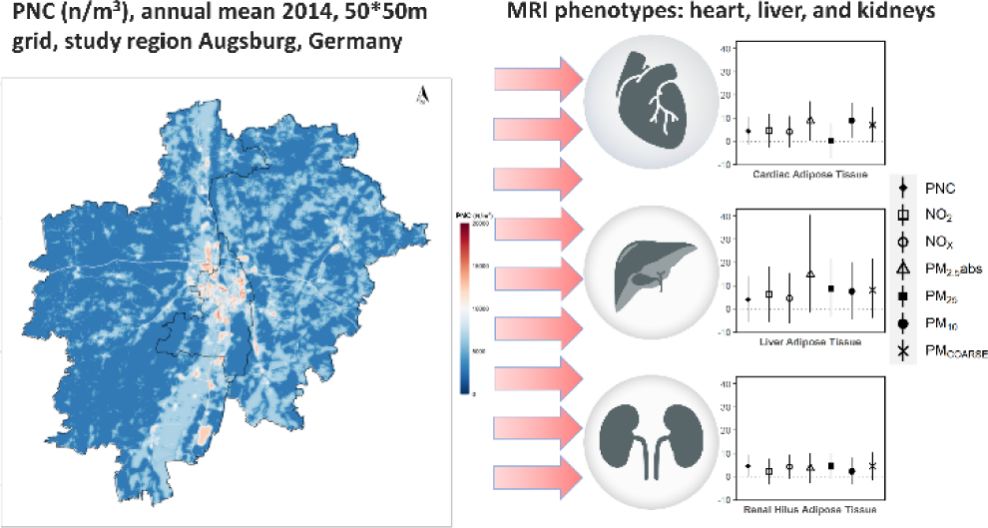

Long-term exposure to traffic-related air pollution (TRAP) is associated with cardiometabolic disease; however, its role in subclinical stages of disease development is unclear. Thus, we aimed to explore this association in a cross-sectional analysis, with cardiometabolic phenotypes derived from magnetic resonance imaging (MRI). Phenotypes of the left (LV) and right cardiac ventricle, whole-body adipose tissue (AT), and organ-specific AT were obtained by MRI in 400 participants of the KORA cohort. Land-use regression models were used to estimate residential long-term exposures to TRAP, e.g., nitrogen dioxides (NO_2_) or particle number concentration (PNC). Associations between TRAP and MRI phenotypes were modeled using linear regression. Participants’ mean age was 56 ± 9 years, and 42% were female. Long-term exposure to TRAP was associated with decreased LV wall thickness; a 6.0 μg/m^3^ increase in NO_2_ was associated with a −1.9% [95% confidence interval: −3.7%; −0.1%] decrease in mean global LV wall thickness. Furthermore, we found associations between TRAP and increased cardiac AT. A 2,242 n/cm^3^ increase in PNC was associated with a 4.3% [−1.7%; 10.4%] increase in mean total cardiac AT. Associations were more pronounced in women and in participants with diabetes. Our exploratory study indicates that long-term exposure to TRAP is associated with subclinical cardiometabolic disease states, particularly in metabolically vulnerable subgroups.

## Abstract 2

Little is known about how traffic-related air pollution (TRAP) is associated with preclinical states of cardio-metabolic disease. This study indicates that long-term exposure to TRAP leads to changes in tissue morphology of the heart, liver, pancreas, and kidneys.

## Introduction

Due to increasing urbanization, more than 70% of all European Union citizens are currently living in cities, towns, and suburbs.^1^ Despite a reduction in emissions, traffic-related air pollution (TRAP) is still the dominant source of outdoor air pollution in urbanized areas.^2^ TRAP refers to a complex mixture of air pollutants that originate directly from vehicle exhausts, or indirectly from motorized vehicles, for example from brakes and tires.^3^ The pollutants emitted that are mainly, but not exclusively, linked to motorized traffic include nitrogen dioxide (NO_2_), ultrafine particles (UFP), black carbon (BC), as well as particulate matter (PM) with an aerodynamic diameter ≤2.5 μm (PM_2.5_) and PM with an aerodynamic diameter ≤10 μm (PM_10_).

Two recent meta-analyses focusing on health effects of TRAP provide evidence, that circulatory mortality and ischemic heart disease, as well as diabetes are associated with TRAP.^4,5^ Results from animal studies suggest that air pollutants trigger an underlying pathway that plays a central role in the pathogenesis of both cardiovascular and metabolic diseases.^6,7^ Previous studies in humans, however, have either focused on cardiovascular or metabolic health outcomes, which has prevented the joint pathway from being examined in the setting of one comprehensive study.

Clinically manifest diseases such as ischemic heart disease, heart failure or obesity are preceded by a longer period of time during which pathological changes occur in the organ tissue that is later affected by the disease, without any clinically observable symptoms.^8^ Examples include a reduced left ventricular (LV) ejection fraction, which precedes heart failure, or an increase in liver fat, which is a precursor to fatty liver disease.^8,9^ Medical imaging, such as magnetic resonance imaging (MRI) or computed tomography (CT) can detect these predisease conditions in an early stage of development. In population-based research, MRI emerges as the gold standard for many applications, due to its sensitivity in detecting tissue alterations within and outside organs, while, compared to CT scans, participants are not exposed to radiation. Nevertheless, studies employing MRI in this context remain relatively scarce. In a large population-based study, air pollution exposure was associated with a larger left ventricular (LV) end-diastolic (EDV) and end-systolic volume (ESV).^10^ Both variables are considered early markers regarding progressive heart failure.^11^ LV wall thickness, an important risk factor for heart failure, had only been investigated in an echocardiography study, where the authors found no significant long-term effects of air pollution on relative LV wall thickness.^12^ Exposure to air pollutants is also known to be associated with structural and functional changes in the right ventricle (RV); for example, larger RV EDV and greater RV mass were associated with NO_2_ exposure^13^ and PM_2.5_.^10^

The association of air pollution with metabolism-related conditions has so far mainly been examined in CT-based studies. A Korean CT study with over 5,000 participants found no association of PM_10_ or NO_2_ with total adipose tissue (TAT), visceral adipose tissue (VAT), or subcutaneous adipose tissue (SAT).^14^ Likewise, the Framingham Heart Study reported no effect of 1-year PM_2.5_ exposure on SAT, VAT,^15^ or hepatic steatosis;^16^ however, both studies found an effect of residential proximity to major roads.

Studies investigating the underlying mechanisms of preclinical disease development leading to TRAP-associated health outcomes are limited. Existing research with medical imaging data has concentrated on assessing exposure to PM_10_, PM_2.5_, NO_2_, and nitrogen oxides (NO_*X*_) with no investigations conducted on other crucial traffic-related air pollutants such as UFP.^10,12−14,16^ Additionally, metabolic mechanisms remain inadequately explored. The influence of air pollutants on adipose tissue (AT) compartments such as cardiac, renal, and pancreatic AT have not been examined so far. However, comprehensive investigations are essential for a thorough understanding of the health implications linked to TRAP. Thus, the objective of this cross-sectional study was to explore the associations between long-term exposure to TRAP (NO_2_, NO_*X*_, particle number concentration (PNC) as a proxy for UFP, PM_10_, PM_2.5_, particles with an aerodynamic parameter between 2.5 and 10 μm (PM_coarse_), and PM_2.5_ absorbance (PM_25_abs) as a proxy for BC) with cardio-metabolic MRI phenotypes in a subsample from a population-based cohort. In our exploratory analysis, we hypothesize that exposure to TRAP is associated with impaired cardiac function, structural alterations in heart tissue, as well as increased abdominal and ectopic AT deposition.

## Methods

### Study Population

KORA-MRI is a cross-sectional imaging substudy nested in the second follow-up (FF4) of the population-based KORA S4 cohort (“Cooperative Health Research in the Region of Augsburg”). The study area is the City of Augsburg in southern Germany and two adjacent districts (Augsburg district, Aichach-Friedberg district). The setting and recruitment of the KORA cohort had already been described in detail.^17^

The FF4 follow-up took place between June 2013 and September 2014 and included 2279 participants, from whom 400 participated in the KORA-MRI substudy. A description of the eligibility and exclusion criteria of the MRI study, and details about the study setup are described elsewhere.^18^ Briefly, individuals were excluded if they were older than 73 years, had any history of cardiovascular disease (CVD) including myocardial infarction, stroke, revascularization, had impaired renal function, or had any contraindications to MRI.

The KORA-MRI study was approved by the institutional review board of the Ludwig-Maximilians-Universität München (LMU Munich) and the KORA FF4 study by the committee of the Bavarian Chamber of Physicians in Munich. All participants gave their written consent.

### Covariate Assessment

Anthropometric measures were taken at the KORA study center, and information about health status, medication intake, social status, physical activity, smoking, and alcohol consumption was derived by standardized questionnaires and interviews.^18^ All individuals without overt diabetes underwent an oral glucose tolerance test and were subsequently classified into three groups (normoglycemia, prediabetes, diabetes) according to WHO criteria.^19^

#### Outcome Assessment (MRI)

Whole body MRI examinations were taken within three months after the visit at the study center at a 3 Tesla MAGNETOM Skyra (Siemens Healthineers, Erlangen, Germany) using an 18 channel body coil in combination with the table-mounted spine matrix coil. The whole-body MRI comprised a comprehensive standardized protocol as described in detail previously.^18^

The detailed description regarding the measurements for cardiovascular and AT parameters can be found in Supplement S1.

We included the following variables as outcomes of interest: left ventricle: LV wall thickness for American Heart Association (AHA) segments^20^ as well as averaged over all segments (global wall thickness), end-systolic volume (ESV), end-diastolic volume (EDV), stroke volume (SV), ejection fraction (EF), diastolic myocardial mass (DMM), LV remodeling, calculated as (DMM/EDV). Right ventricle: EDV, ESV, SV, EF. Vessels diameter: Ascending aorta, infrarenal aorta, pulmonary trunk, right pulmonary artery, left pulmonary artery. Adipose tissue: Total abdominal adipose tissue (TAT), visceral abdominal adipose tissue (VAT), subcutaneous abdominal adipose tissue (SAT), total epi-and pericardial adipose tissue (cardiac AT), epicardial systolic adipose tissue, epicardial diastolic adipose tissue, pericardial systolic adipose tissue (PSAT), pericardial diastolic adipose tissue (PDAT), renal hilus AT, mean AT content of the liver and mean AT content of the pancreas.

Missing values in MRI data were due to insufficient image quality or technical malfunctions and were unrelated to exposure data or participants’ clinical characteristics.

### Exposure Assessment (Air Pollution)

Air pollution exposure was estimated as annual mean concentrations of the respective air pollutants in the time period March 2014 to April 2015 for all KORA FF4 participants using land-use regression (LUR) models.^21^ The respective residential pollution exposure was then assigned to each participant’s home address. The models are based on air pollution concentrations sampled at 20 measurement stations (located at urban traffic, urban background, rural traffic, and rural background sites) within the Augsburg study region during three 14-day periods. Measurements were taken in the cold, warm, and intermediate (spring, autumn) seasons between March 2014 and April 2015. PNC was measured by three GRIMM ultrafine particle counters (model EDM 465 UFPC, GRIMM aerosol, Ainring, Germany) and one NanoScan SMPS Nanoparticle Sizer (model 3910, TSI, Shoreview, MN, USA). Nitrogen oxides (NO_*X*_ and NO_2_) were measured with Ogawa passive samplers (Ogawa & Co., USA Inc.), while PM_10_ and PM_2.5_ were sampled using Harvard Impactors. PM_coarse_ was calculated as the difference between PM_10_ and PM_2.5_. Reflectance was measured on PM_10_ and PM_2.5_ filters and transformed into absorbance (PM_2.5_abs). According to previous studies, PM_2.5_abs was used as a proxy for BC.^22^ PM_2.5_abs has previously been found to be highly correlated with elemental carbon.^23^ As in Germany one-third of the private vehicles use diesel^24^ which is one major source of PM_2.5_abs, in this study area PM_2.5_abs is supposed to mainly reflect traffic related emissions. Besides the data from the measurement stations, information on spatial predictors was collected. The exposure models were built by regressing annual averages of the air pollution concentrations from the measurement stations against spatial predictors. All models comprised at least one predictor for traffic within a small buffer up to 100 m.

Detailed information about measurement techniques, model predictors, missing data, validation, and model quality can be found elsewhere.^21^

### Statistical Methods

All continuous variables were visually examined for normal distribution. Normally distributed variables were reported as mean and standard deviation, and variables with non-normal distributions as median and interquartile range (IQR). Differences in the study population characteristics and the air pollution variables according to sex were explored by *t* tests, Wilcoxon rank-tests, or chi-square tests, as applicable.

We calculated linear regression models to assess the association between long-term exposure to air pollution and cardiovascular or AT outcome variables. Two separate covariate models were developed to account for differences in the impact of individual covariates within the exposure-response relationship for cardiovascular and AT outcomes. Non-normal distributed outcomes were natural log-transformed to increase normality of residuals. We selected the covariates a priori in a multistep approach based on the disjunctive cause criterion (Supplement S2).^25^ Two independent covariate models were developed: one model for cardiovascular outcomes and one for AT. Age and sex were forced into each model. The following variables were tested for inclusion into the models: weight, height, waist circumference, body-mass-index (BMI), waist-to-hip-ratio, systolic blood pressure (SBP), diastolic blood pressure (DBP), pulse pressure (PP), cholesterol, high-density lipoprotein (HDL), low-density lipoprotein (LDL), triglycerides (TAG), alcohol consumption, diabetes status, hypertension, angina pectoris symptoms, intake of antihypertensive medication, intake of lipid-lowering medication, intake of antidiabetic medication, household income, marital status, years of education, smoking, and physical activity. To avoid multicollinearity, we tested the covariates for correlation before including them into the models.

Basic models have been calculated for all outcomes, adjusting for age and sex only. The minimum model for cardiovascular outcomes was adjusted for age, sex, height, and BMI. The main model was adjusted for age, sex, height, BMI, income, marital status, years of education, and smoking. We built two extended models: the first one additionally included high-density-lipoprotein (HDL) and systolic blood pressure (SBP), the second one diabetes status and intake of lipid-lowering medication.

The resulting minimum model for AT was adjusted for age, sex, and height. The main model additionally contained income and physical activity. The first extended model included TAG and PP, the second diabetes status and intake of antidiabetic medication. BMI was intentionally excluded from the AT analysis and is instead part of the sensitivity analysis.

In order to assess the robustness of our results, we performed the following sensitivity analyses based on our main models: (1) additional adjustment for high sensitive C-reactive protein (hs-CRP). (2) Additional adjustment for neighborhood socioeconomic status, (3) exclusion of all participants with late gadolinium enhancement (LGE) on MRI. (4) We did not adjust the AT models in the main analysis for BMI, as BMI can be considered as biometrical measure of body AT, and thus we might have statistically eliminated the effect we wanted to examine. To consider BMI in the covariate model, we performed a sensitivity analysis additionally adjusting the AT models for BMI. (5) We performed two-pollutant models for all outcomes of interest.

In further analyses, we performed the following stratifications: (1) sex female vs male, (2) age <65 years vs ≥65 years, (3) normoglycemia vs prediabetes vs diabetes, (4) BMI < 30 kg/m^2^ vs ≥30 kg/m^2^, (5) hs-CRP < 1 mg/L vs ≥1 mg/L, participants with hs-CRP > 10 mg/dl were excluded, (6) hypertension yes vs no.

Sample size was based on a complete case analysis per outcome since exposure data were available for all participants. Results were reported either as %-change [95% confidence intervals] of the outcome mean, or as %-change [95% confidence intervals] of the geometric outcome mean (in case of log-transformed outcomes) per interquartile range (IQR) increase in the respective air pollutant.

P-values <0.05 were considered statistically significant; all reported values were two-tailed. Statistical analyses were performed using R version 3.6.2 (The R Foundation for Statistical Computing, Vienna, Austria).

## Results

### Study Sample

The mean age was 56 ± 9 years, and 42% of the participants were female (Table 1). With a mean BMI of 28 ± 5 kg/m^3^ and a mean LDL of 140 ± 33 mg/dl, participants showed elevated cardiovascular risk factors.

**Table 1:** Characteristics of the Study Population^a^

**Participant Characteristics** (all participants: *n* = 400)	**Mean (SD)**
Age [years]	56.3 (9.2)
Weight [kg]	83.0 (16.6)
Height [cm]	171.6 (9.7)
Waist circumference [cm]	98.6 (14.3)
BMI [kg/m^3^]	28.1 (4.9)
WHR	0.9 (0.1)
SBP [mmHg]	120.6 (16.7)
DBP [mmHg]	75.3 (10.0)
PP [mmHg]	71.3 (9.9)
Cholesterol [mg/dl]	217.8 (36.3)
HDL [mg/dl]	61.9 (17.7)
LDL [mg/dl]	139.5 (32.9)
TAG [mg/dl]	131.5 (84.8)
Neighborhood SES	22.4 (21.7)
	**Median(IQR)**
Alcohol consumption [g/day]	8.5 (25.7)
hsCRP [mg/L]	1.2 (1.9)
	***N*** **(%)**
*Sex*	
Female	169 (42%)
Male	231 (58%)
*Diabetes status*	
Diabetes	54 (14%)
Prediabetes	103 (26%)
Normoglycemia	243 (60%)
Hypertension	136 (34%)
Angina pectoris	25 (6%)
Antihypertensive medication	102 (26%)
Lipid lowering medication	43 (11%)
Antidiabetic medication	32 (8%)
*Household income per month*	
<625€	14 (4%)
625€ to <1250€	106 (27%)
1250€ to <1875€	192 (48%)
1875€ to <2500€	11 (3%)
≥2500€	59 (14%)
Missing	18 (4%)
*Marital status*	
Unmarried, living alone	39 (10%)
Unmarried, living with the partner	15 (4%)
Married, living with the spouse	289 (72%)
Married, living apart	9 (2%)
Divorced	31 (8%)
Widowed	17 (4%)
*Years of education*	
8	10 (3%)
10	137 (34%)
11	55 (14%)
12	38 (9%)
13	80 (20%)
15	5 (1%)
17	77 (19%)
*Smoking habits*	
Regular	80 (20%)
Former	174 (44%)
Never	146 (36%)
*Physical activity*	
Very active	115 (29%)
Moderate active	123 (31%)
Little active	57 (14%)
Nonactive	105 (26%)
**MRI Parameter**	**Mean (SD)**
**Left Ventricle(included participants:***n* = 379)	
End-diastolic volume [ml]	129.1 (33.0)
End-systolic volume [ml]	40.8 (18.1)
Stroke volume [ml]	88.4 (20.7)
Ejection fraction [%]	69.2 (8.2)
Diastolic myocardial mass [g]	140.7 (35.1)
*Diastolic average wall thickness*	
Basal segments [mm]	10.0 (1.7)
Mid segments [mm]	9.6 (1.8)
Apical segments [mm]	8.4 (1.5)
Lateral segments [mm]	9.8 (1.6)
Septal segments [mm]	9.5 (1.7)
Anterior segments [mm]	9.4 (1.9)
Inferior segments [mm]	9.4 (1.5)
Global segments [mm]	9.5 (1.5)
Left ventricular remodeling [g/ml]	1.1 (0.3)
**Right Ventricle (included participants:***n* = 337)	
End-diastolic volume [ml]	165.5 (39.8)
End-systolic volume [ml]	79.1 (25.9)
Stroke volume [ml]	86.4 (19.5)
Ejection fraction [%]	52.8 (7.0)
**Vessels(included participants:***n* = 371)	
Diameter ascending Aorta [cm]	3.3 (0.4)
Maximum diameter infrarenal Aorta [cm]	1.5 (0.2)
Diameter pulmonary trunk [cm]	2.7 (0.3)
Diameter right pulmonary artery [cm]	1.8 (0.3)
Diameter left pulmonary artery [cm]	1.9 (0.2)
**Whole Body AT(included participants:***n* = 384)	
Total AT [l]	12.6 (5.5)
Visceral AT [l]	4.5 (2.7)
Subcutaneous AT [l]	8.1 (3.7)
**Cardiac AT(included participants:***n* = 341)	
Epi- and pericardial AT [ml]	130.3 (73.3)
Systolic epicardial AT [cm^2^]	8.9 (4.6)
Systolic pericardial AT [cm^2^]	29.8 (16.5)
Diastolic epicardial AT [cm^2^]	8.2 (4.3)
Diastolic pericardial AT [cm^2^]	27.0 (15.4)
**Liver and Pancreatic AT(included participants:***n* = 384)	
Mean AT content of the liver [%]	8.9 (8.1)
Mean AT content of the pancreas [%]	7.7 (7.0)
**Renal AT(included participants:***n* = 366	
Renal hilus AT [ml]	40.0 (18.0)
**Environmental Exposure(all participants:***n* = 400)	**Mean (IQR)**
PM_10_ [μg/m^3^]	16.5 (2.1)
PM_2.5_ [μg/m^3^]	11.7 (1.4)
PM_coarse_ [μg/m^3^]	4.8 (1.5)
PNC [n/cm^3^]	7,076.8 (2,241.8)
NO_2_ [μg/m^3^]	13.6 (6.0)
NO_*X*_ [μg/m^3^]	21.1 (9.7)
PM_25_abs [10^–5^ m^–1^]	1.2 (0.3)

a SD: standard deviation; IQR: interquartile range; BMI: body mass index; WHR: waist-to-hip ratio; SBP: systolic blood pressure; DBP: diastolic blood pressure; PP: pulse pressure; HDL: high density lipoprotein; LDL: low density lipoprotein; TAG: triacylglycerides; SES: socio-economic status; hsCRP: high sensitive c-reactive protein; AT: adipose tissue; PM_10_: particulate matter with an aerodynamic diameter ≥10 μm; PM_2.5_: particulate matter with an aerodynamic diameter ≥2.5 μm; PM_coarse_: particles with an aerodynamic parameter 10-2.5 μm; PNC: particle number concentration; NO_2_: nitrogen dioxide; NO_*X*_: nitrogen oxides; PM_25_abs: PM2.5 absorbance.

**Figure 1:**
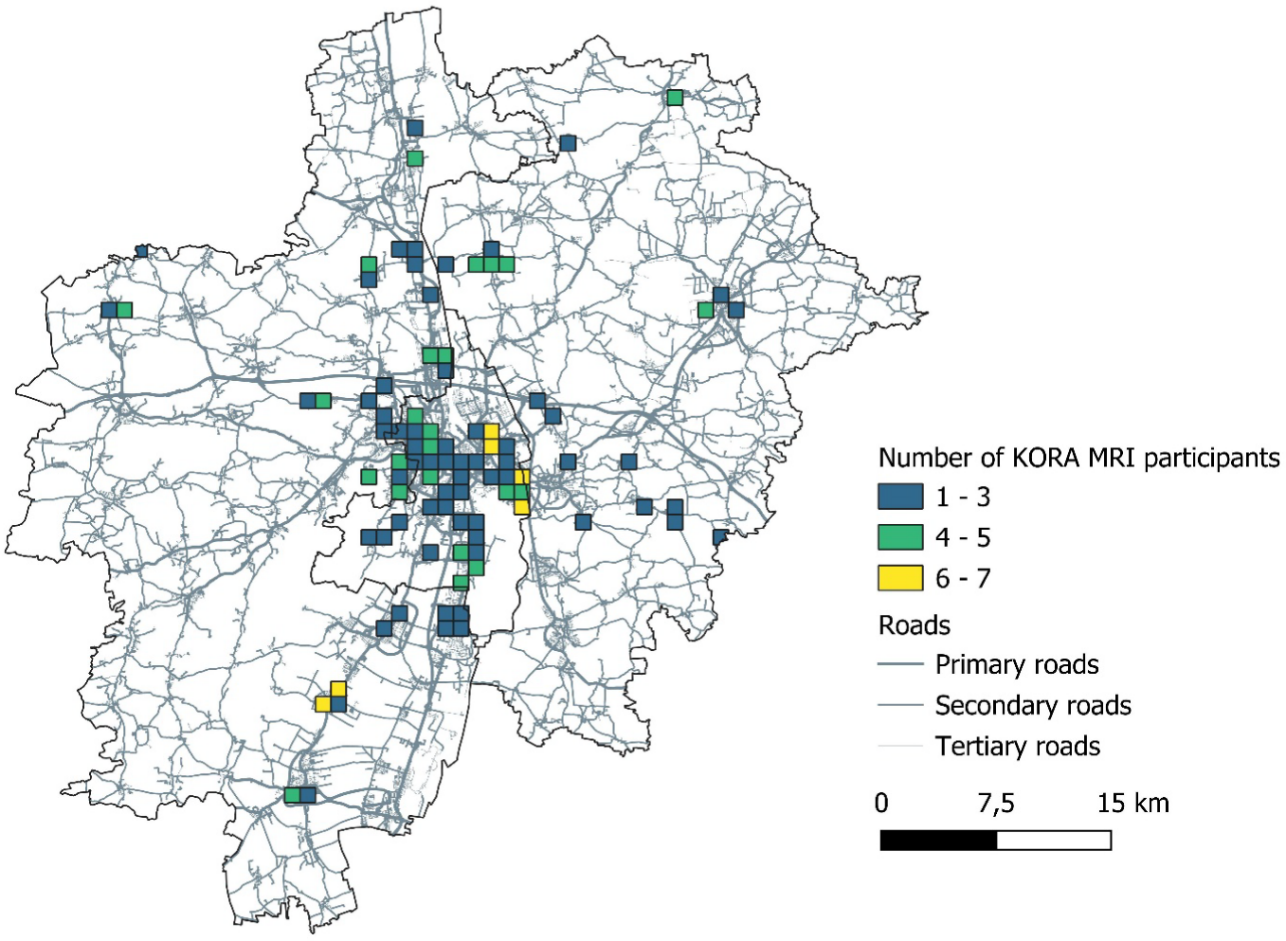
Number of participants of the KORA-MRI Study per 1 × 1 km grid. Map of the city of Augsburg, Augsburg County, and Aichach-Friedberg County.

Data on MRI outcomes and the number of participants included in respective analyses are presented in Table 1, Figure 1 provides a map with the distribution of the participant’s residences within the study area.

There were no significant differences in air pollution exposures and outcomes between women and men (Tables S1 and S2). With a mean annual PM_2.5_ exposure of 11.7 μg/m^3^ (Tables 1 and S3), the participants’ exposure levels were below the EU annual limit of 25 μg/m^3^, but above the WHO recommendation of an annual mean of 5 μg/m^3^. The same applies to NO_2_ (13.6 μg/m^3^ compared to 40 μg/m^3^ (EU) and 10 μg/m^3^ (WHO)) and PM_10_ (16.5 μg/m^3^ compared to 40 μg/m^3^ (EU) and 15 μg/m^3^ (WHO)).^26,27^

#### Air Pollution and Cardiovascular Outcomes

**Figure 2:**
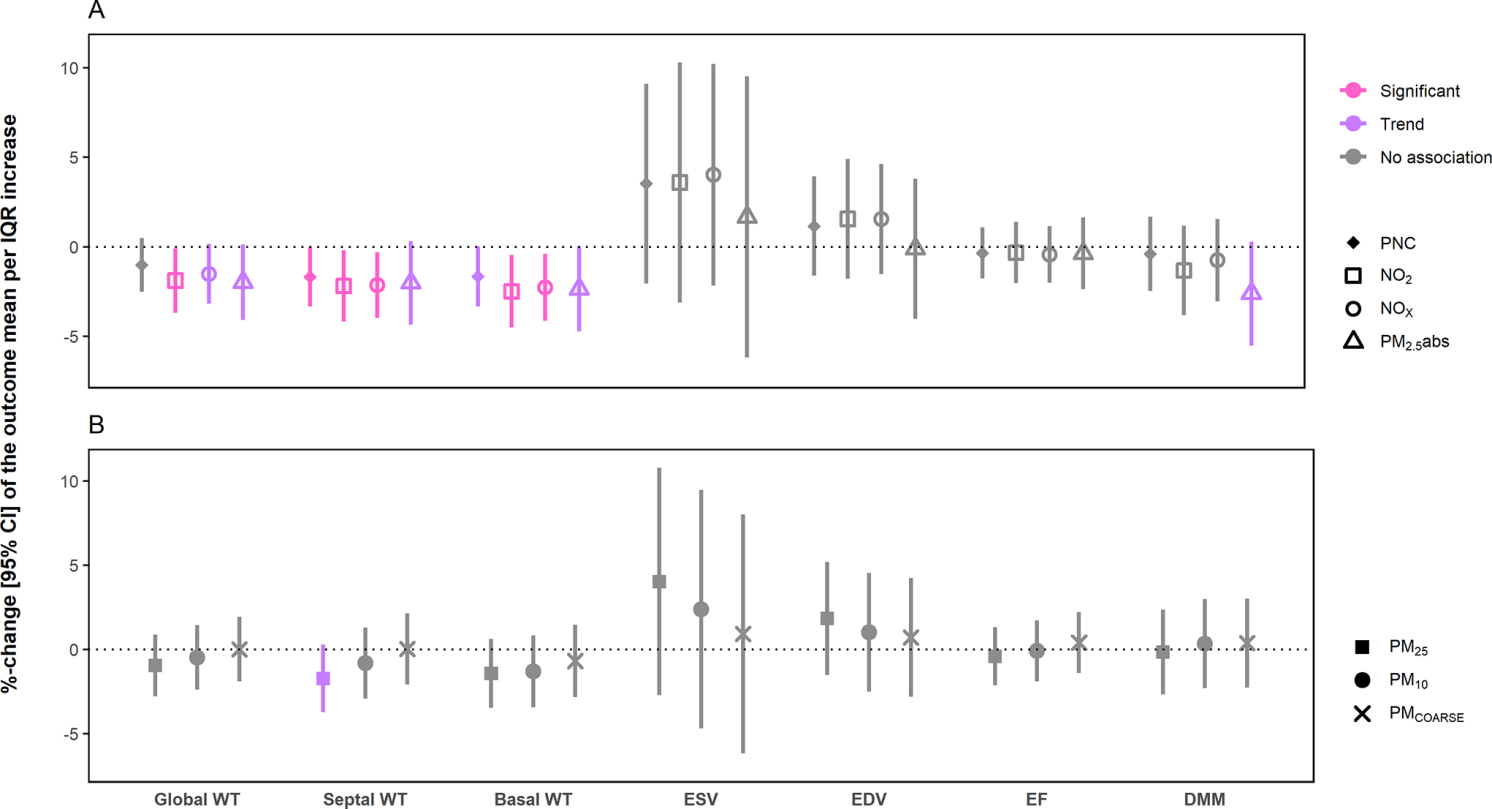
Association between long-term exposure to TRAP and selected parameter of the left ventricle. The plot is divided into panels (A,B) for better readability. Results are presented as %-change of the outcome mean and 95% confidence intervals, per IQR increase in the respective air pollutant. Strength of association: significant: *p*-value <0.05. Trend: 0.1 > *p*-value ≥ 0.05. No association: *p*-value ≥0.1. Global WT: global diastolic left ventricular wall thickness. Septal WT: septal diastolic left ventricular wall thickness. Basal: basal diastolic left ventricular wall thickness. ESV: end-systolic volume. EDV: end-diastolic volume. EF: ejection fraction. DMM: diastolic myocardial mass. Models were adjusted for age, sex, height, BMI, income, marital status, years of education, and smoking.

Our results showed a significant negative association between long-term exposure to residential air pollution and global LV diastolic wall thickness (Figure 2 and Table S4). An IQR increase in NO_2_ was associated with a −1.89% [−3.70%, −0.09%] decrease in global LV wall thickness. Similar patterns were found for NO_2_ exposure and septal, basal, lateral, and inferior segments. Exposure to NO_*X*_, PM_2.5_abs, and, to some extent, PNC also showed negative associations with average wall thickness in different segments, e.g., an IQR increase in NO_*X*_ was associated with a −2.16% [−3.98%, −0.33%] decrease in septal LV wall thickness. Furthermore, TRAP exposure showed a pattern toward an association regarding functional parameters of the LV or RV (Table S5). In analyses with vessels, we saw a weak positive association between exposures to NO_*X*_ or PNC and the mean diameter of the ascending aorta (Table S5).

#### Sensitivity Analyses

Additional adjustment for either hs-CRP or neighborhood SES or exclusion of participants with LGE did not significantly alter the observed results (results not shown). Additionally adjusting the models for a second pollutant (two-pollutant models, Table S19) did not change the observed associations from the main models.

#### Stratified Analyses

**Figure 3:**
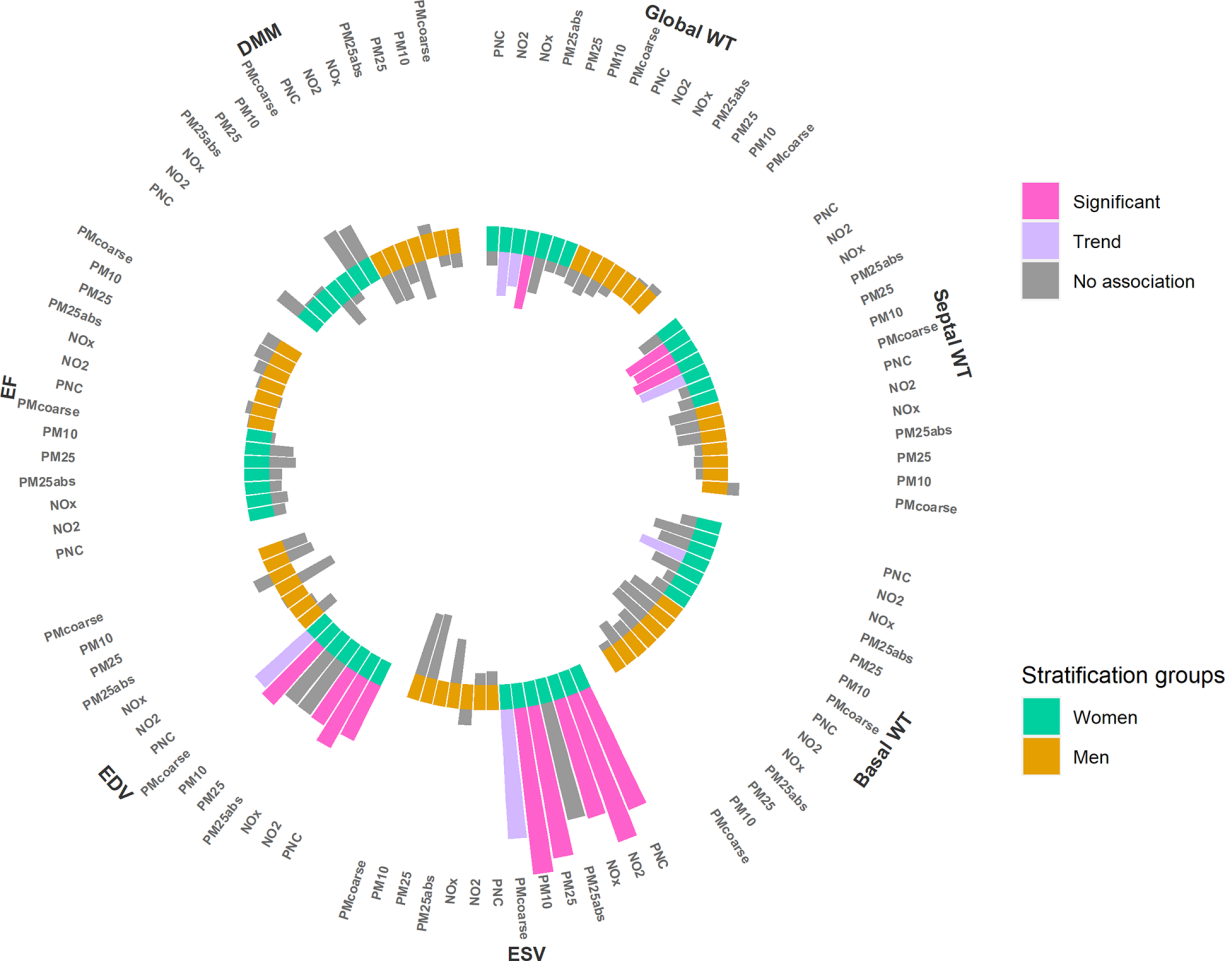
Association between long-term exposure to TRAP and selected LV parameters, stratified by sex. The height of the bars indicates the effect size. The direction of the bars indicates the direction of the association. Bars toward the center indicate a negative association, bars toward outside a positive association. Strength of association: significant: *p*-value < 0.05. Trend: 0.1 > *p*-value ≥ 0.05. No association: *p*-value ≥ 0.1. WT: diastolic left ventricular wall thickness. ESV: end-systolic volume. EDV: end-diastolic volume. EF: ejection fraction. DMM: diastolic myocardial mass. Models were adjusted for age, sex, height, BMI, income, marital status, years of education, and smoking.

In sex-stratified analyses, the negative associations for LV wall thickness were only observed in women (Figure 3 and Table S7). Additionally, in women we observed positive associations between various air pollutants and ESV and EDV. When stratified by age categories, the negative association between air pollution and LV wall thickness we observed in the main model was only present for participants older than 64 years (Table S6). Stratification by diabetes status showed associations mainly in participants with diabetes (Table S8). Additional information about stratified analyses is provided in Tables S9–S11.

### Air Pollution and Adipose Tissue

**Figure 4:**
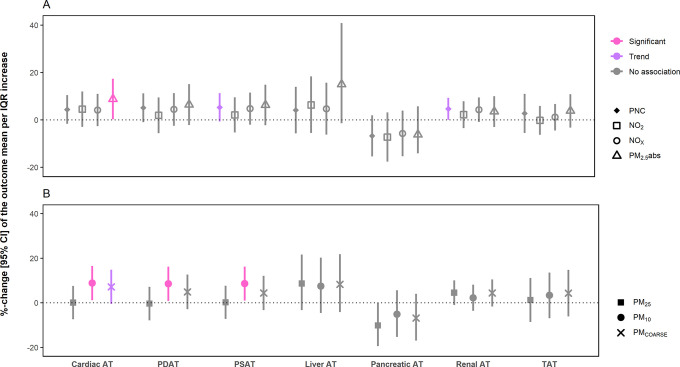
Association between long-term exposure to TRAP and the adipose tissue of heart, liver, pancreas, kidneys, and total adipose tissue. The plot is divided into panels (A,B) for better readability. Results are presented as %-change of the outcome mean and 95% confidence intervals, per IQR increase in the respective air pollutant. Strength of association: significant: *p*-value < 0.05. Trend: 0.1 > *p*-value ≥ 0.05. No association: *p*-value ≥ 0.1. Cardiac AT: Epi- and pericardial adipose tissue. PDAT: pericardial diastolic adipose tissue. PSAT: pericardial systolic adipose tissue. Liver AT: mean adipose content of the liver. Renal AT: renal hilus adipose tissue. Pancreatic AT: mean adipose content of the pancreas. TAT: total abdominal adipose tissue. Models were adjusted for age, sex, height, income, and physical activity.

We observed a significant positive association between long-term exposure to TRAP and cardiac AT. A similar trend, that means an indication for an association, was observed for liver and renal hilus AT, while the associations for pancreatic AT were in the opposite direction (Figure 4 and Table S12). An IQR increase in PM_10_ was associated with an 8.83% [1.19%, 16.49%] increase in total epi- and pericardial AT, and comparable effect estimates were seen for diastolic and systolic pericardial AT (8.53% [0.84%, 16.23%] and 8.57% [0.10%, 16.17%], respectively. A similar pattern was found for PNC; an IQR increase was associated with an 4.33% [−1.72%, 10.39%] increase in mean total epi- and pericardial AT. No association was seen between TRAP and TAT, VAT, or SAT.

#### Sensitivity Analyses

Sensitivity analyses with additional adjustment for either hs-CRP or neighborhood SES, or exclusion of participants with LGE did not change the results considerably. When additionally adjusting our AT models for BMI, all significant associations disappeared (results not shown). In the two-pollutant models, the observed associations remained mainly unchanged (Table S20).

#### Stratified Analyses

**Figure 5:**
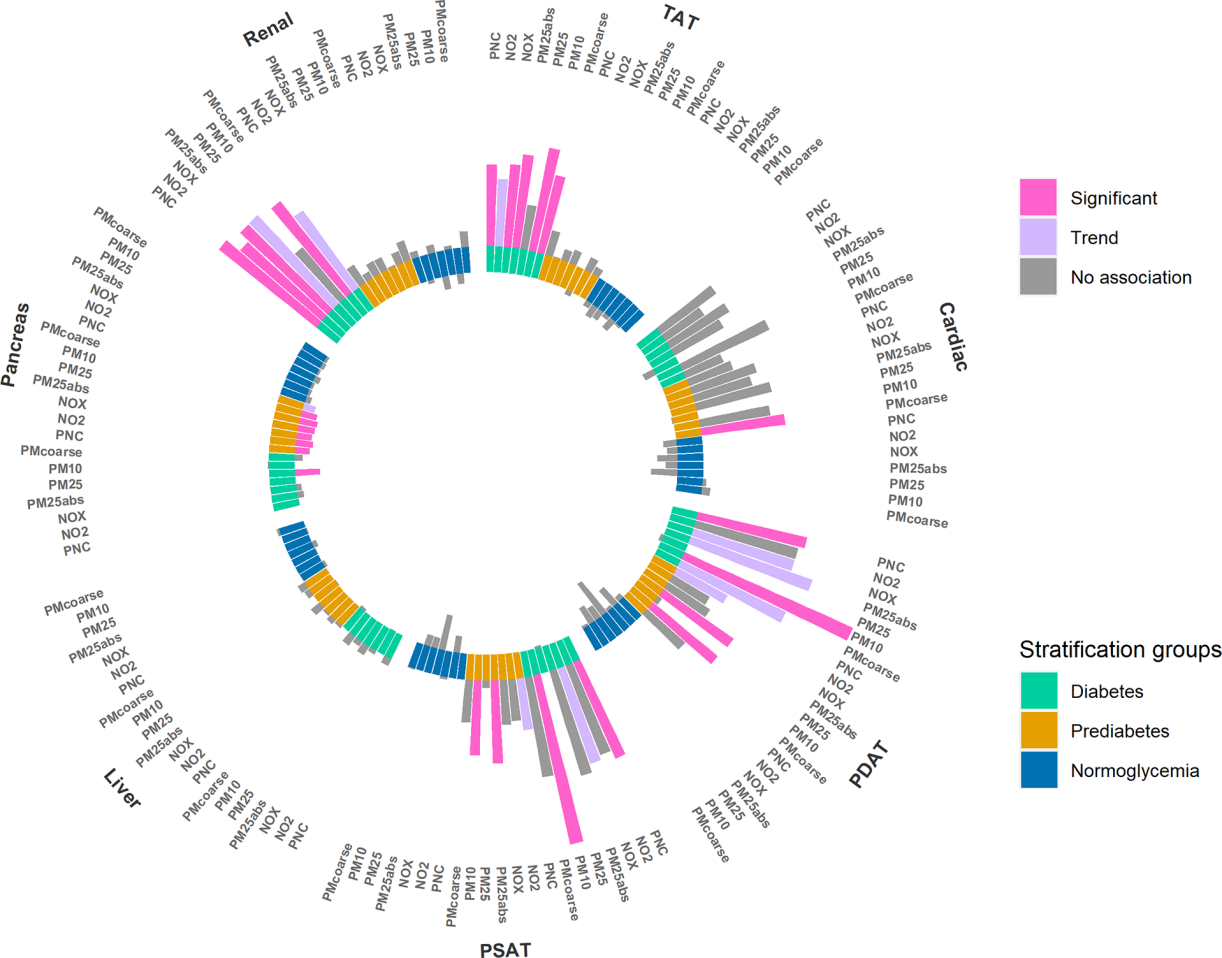
Association between long-term exposure to TRAP and the adipose tissue of heart, liver, pancreas, kidneys, and total abdominal adipose tissue, stratified by diabetes status. The height of the bars indicates the effect size. The direction of the bars indicates the direction of the association. Bars toward the center indicate a negative association, bars toward outside a positive association. Strength of association: Significant: *p*-value < 0.05. Trend: 0.1 > *p*-value ≥ 0.05. No association: *p*-value ≥ 0.1. TAT: total abdominal adipose tissue. Cardiac: epi- and pericardial adipose tissue. PDAT: pericardial diastolic adipose tissue. PSAT: pericardial systolic adipose tissue. Liver: mean adipose content of the liver. Pancreas: mean adipose content of the pancreas. Renal: renal hilus adipose tissue. Models were adjusted for age, sex, height, income, and physical activity.

The positive association between TRAP and cardiac AT was only present in women, participants younger 65 years and participants with hypertension (Tables S13, S14 and S18,). Furthermore, in participants with diabetes, we observed a positive association between several air pollutants and TAT, as well as for cardiac and renal hilus AT (Figure 5 and Table S15), but opposite trends for pancreatic AT. Additional results from stratified analyses can be found in Tables S16 and S17.

## Discussion

In this cross-sectional analysis, we investigated the association between long-term exposure to traffic-related air pollution and cardio-metabolic MRI phenotypes in a sample from a population-based cohort without prior CVD. Our main findings were: (1) TRAP such as NO_2_, NO_*X*_, PM2_5abs_ and PNC were associated with decreasing LV wall thickness; (2) as a trend, TRAP was associated with functional LV parameters; (3) as a trend, TRAP was associated with increased cardiac, liver and renal AT; (4) women and participants with unfavorable metabolic characteristics tended to be more vulnerable to TRAP.

Even at low concentrations, traffic-related emissions have been shown to have adverse effects on health outcomes, including cardiovascular mortality.^4,28,29^ As these health effects may depend on the source, TRAP should be considered in air pollution studies due to its varying composition.^3^

Our results suggest that increased TRAP exposure is associated with a global and segment-specific decrease in LV wall thickness. LV wall thickness plays a predictive role in cardiovascular mortality and contributes to cardiac remodeling.^30^ TRAP related decrease in wall thickness may serve as an early indicator regarding air pollution-driven cardiac wall remodeling toward preclinical dilated cardiomyopathy. After coronary artery disease, dilated cardiomyopathy is a leading cause of heart failure.^31^ A recent UK study found significant associations between long-term NO_2_ exposure and LV remodeling in patients with dilated cardiomyopathy,^32^ particularly in women. This aligns with our results, suggesting that women are more susceptible to harmful effects of air pollution on LV wall thickness.

Functional parameters of the LV and RV are crucial in developing cardiac dilatation.^11,33^ Comparable to our results, a large MRI study found long-term exposure to air pollution being associated with higher LV EDV and ESV.^10^ Additionally, we observed a trend toward an increasing diameter of the ascending aorta, supporting the hypothesis of increased cardiac dilatation.

We must stress that regular function and morphology are defined by MRI parameters within a range. Both lower and higher values outside the range can indicate pathologies; thus, associations pointing to an increase or decrease in cardiac parameters require a nuanced interpretation. A decrease in wall thickness might indicate an early dilatation, whereas an increase in wall thickness might indicate a beginning myocardial stiffening, depending on the initial wall thickness values. Since our sample was free of overt CVD, and values were within the nonpathological range, the associations we report have to be interpreted as subclinical changes, indicating potentially progressing disease.

Our novel findings in relation to TRAP and cardiac AT are particularly relevant in the context of cardiovascular disease development, such as heart failure, where pericardial AT serves as a significant adverse prognostic marker.^34,35^ We found associations between exposure to PM_10_, PM_25abs_ or PNC and increasing total cardiac AT or pericardial AT. This might indicate that chronic air pollution exposure acts as a driver toward pathological cardiac AT deposition.

In the same line, exposure to TRAP showed a trend toward higher renal hilus and liver AT. While there a no studies on renal AT in humans, Li et al. (2017) found significant associations with proximity to major roads but not with PM_2.5_ exposure on liver AT content.^16^

The complex interplay between type 2 diabetes and AT metabolism has been the focus of several studies,^36,37^ but few have looked at the interference of air pollution exposure. In our study, after stratification according to diabetes status the observed associations between exposure to TRAP and TAT were only present in individuals with diabetes. The same applies for cardiac AT, PSAT, PDAT, and renal hilus AT, where we detected an association with TRAP exposure only in diabetics or prediabetics. Other imaging studies on this topic are scarce. An U.S. study on long-term PM_2.5_ exposure and fatty liver disease found a positive association, which remained unaffected by diabetes status.^38^ The association of TRAP with decreased pancreatic fat in our study deserves further investigation. While it could be hypothesized that the neuroendocrine stress response induced by sustained exposure to TRAP leads to a redistribution of adipose tissue (e.g., a shift from pancreatic to hepatic fat), this is highly speculative. Given the small data set and cross-sectional nature of our study, we strongly advise to interpret this result with caution, since it might be due to residual confounding, or an underestimation of pancreatic fat content.

One important common underlying mechanism of the effects on cardio-metabolic diseases development is subclinical inflammation triggered by air pollution.^39−41^ Activation of inflammatory pathways causes oxidative stress and endothelial dysfunction. The air pollution-driven activation of proinflammatory factors like interleukin-6 and tumor necrosis factor α,^6,7^ leads, among others, to adipocyte accumulation.^42^ The subsequent accumulation of ectopic fat is associated with an increased risk of developing both, clinically manifest cardiovascular and metabolic diseases.^9,37^

TRAP is composed of various components^3^ that, when considered individually, have different pathophysiological mechanisms. After inhalation, PM accumulates in the lungs, where, in particular, ultrafine particles can translocate into the pulmonary circulation.^43^ The formation of reactive oxidative species leads to an increase in oxidative stress, which in turn impairs vascular function.^43^ The accumulation of particulate matter in the lungs also induces an inflammatory response, which leads to an increase in pro-inflammatory biomarkers.^44^ Gaseous pollutants such as NO_2_ and NO_*X*_ are oxidizing gases that lead to an increase in oxidative stress by reducing important antioxidants.^45^ However, since both particulate matter and gaseous pollutants occur together in terms of TRAP and have the same emission sources, it must be assumed that the observed long-term effects also arise from the interaction of the various components of the pollutant mixture.^46^

Metabolically vulnerable subgroups, such as the elderly, participants with diabetes or prediabetes, high BMI, or elevated hs-CRP levels, appear to exhibit an increased susceptibility to the detrimental effects of air pollution exposure. In a mouse study, diabetic mice exposed to diesel exhaust particles showed increased AT contents, while nondiabetic mice did not.^47^ In the elderly population, vascular endothelial dysfunction is more pronounced due to lower bioavailability of protective nitric oxide molecules synthesized by the endothelium.^48^ This further limits the ability to cope with oxidative stress caused by air pollution, leading to an increased susceptibility to harmful air pollutant effects. Furthermore, sex-specific susceptibility to air pollution-related cardiovascular conditions, as indicated by our results and corroborated by previous studies,^32^ underscores the need for a more in-depth exploration of underlying mechanisms contributing to this vulnerability in women. Evidence from animal studies suggests that sex hormones may be a key driver of the observed differences between women and men.^49^ Data from a large-scale study with mice, for example, show that high testosterone levels were cardioprotective against the harmful effects of exposure to PM_2.5_.^50^ Both, our study and others emphasize the significance of influencing factors, including age, sex, lifestyle habits, and preexisting conditions such as diabetes, in rendering individuals more susceptible to air pollution health impacts.^38,51^

A main strength of the current study is the comprehensive panel of exposures and outcomes. While previous studies have only looked at selected air pollutants and focused on particular systems such as the functional heart parameter or specific fat compartments, we examine a broad panel of both, air pollutants and MRI outcomes. Traffic-related pollutants such as PNC have not been analyzed in cardio-metabolic imaging studies before, and the availability of both cardiovascular and body fat composition phenotypes allows for the analysis of shared underlying mechanisms. Due to the large number of air pollution and outcome variables potential exposure-response relations could be explored. Moreover, MRI provides detailed and robust measurements and is considered the gold standard for evaluating cardiac function and morphology as well as volumetric AT. Furthermore, the MRI study is part of a carefully conducted cohort study, comprising information on various covariates which allow for a comprehensive model adjustment.

However, this study faces several limitations. Due to the relatively small number of 400 participants, the statistical power was limited. As we performed multiple analyses in an exploratory fashion, we cannot rule out the possibility that some results were observed by chance. We decided not to adjust for multiple testing, but rather to interpret the observed results as pattern, indicating similar associations in correlated outcomes for different air pollutants from the same source. Moreover, the time period of the MRI measurements (2013–14) and the modeled annual average concentrations in TRAP (2014–15) do not align. However, previous studies have demonstrated, that spatial contrasts remain stable over long periods of time.^22,52^ In addition, air pollution exposure was modeled only at the place of residence. If people spend a considerable amount of time away from home, for example at work, this could lead to an underestimation of exposure, especially for participants who live in the countryside and work in the city. Relocations during the exposure period were also not taken into account. Furthermore, we had only cross-sectional data available, with a small study region, little variation in the study population regarding racial background, and with exclusion criteria for MRI. This limits the generalizability; however we have previously shown through weighted analyses that results are valid for a much larger underlying cohort.^53^

Our study provides further evidence that long-term exposure to different air pollutants is associated with subclinical changes within the cardio-metabolic system. Although our study has an explorative character, it provides an essential contribution to an improved understanding of the role of environmental risk factors in the context of cardio-metabolic disease progression or development. The increased susceptibility of various subgroups is of particular public health relevance and should be further investigated.

## Supporting Information Available

MRI measurements (supplement S1); sequential covariate models (supplement S2); participant characteristics and environmental exposure (Table S1); outcome description (Table S2); correlation matrix for air pollutants (Table S3); air pollution and left ventricular diastolic average wall thickness (Table S4); air pollution and cardiovascular outcomes (Table S5); stratified analyses: air pollution and cardiovascular outcomes (Tables S6–S11); air pollution and adipose tissue outcomes (Table S12); stratified analyses: air pollution and adipose tissue outcomes (Tables S13–S18); two-pollutant models and cardiovascular outcomes (Table S19); two-pollutant models and adipose tissue outcomes (Table S20) (PDF)
