# Supplementary material for: Association between Long-Term Exposure to Traffic-Related Air Pollution and Cardio-Metabolic Phenotypes: An MRI Data-Based Analysis
Source: Environ Sci Technol. 2024 Oct 4;58(41):18064–75. doi: 10.1021/acs.est.4c03163 (PMC11483729; doi:10.1021/acs.est.4c03163)
Supplement: Supplementary file 1 — es4c03163_si_001.pdf [file es4c03163_si_001.pdf]

Association between long-term exposure to traffic-related air pollution and cardio-metabolic phenotypes – an MRI-data based analysis

Margarethe Woeckel* [1,2], Susanne Rospleszcz [1,2,4], Kathrin Wolf [1], Susanne Breitner-Busch [1,2], Michael Ingrisch [3], Fabian Bamberg [4], Jens Ricke [3], Christopher L Schlett [4], Corinna Storz [5], Alexandra Schneider [1], Sophia Stoecklein [3], Annette Peters [1,2,6].

1) Institute of Epidemiology, German Research Center for Environmental Health, Helmholtz Zentrum München, Neuherberg, Germany.

2) Chair of Epidemiology, Institute for Medical Information Processing, Biometry and Epidemiology, Medical Faculty, Ludwig-Maximilians-Universität München (LMU Munich), Munich, Germany.

3) Department of Radiology, Ludwig-Maximilians-Universität Hospital Munich, Munich, Germany.

4) Department of Diagnostic and Interventional Radiology, Medical Center, University of Freiburg, Faculty of Medicine, Freiburg, Germany.

5) Department of Neuroradiology, Medical Center, University of Freiburg, Freiburg, Germany.

6) German Center for Cardiovascular Disease Research (DZHK), Munich Heart Alliance, Munich, Germany.

*Corresponding author

Table of contents

Supplement S1 – MRI measurements S3

Supplement S2 – Sequential covariate models. S6

Supplement Table S1: Participant characteristics and environmental exposure according to sex S7

Supplement Table S2: Outcome description according to sex S10

Supplement Table S3: Correlation matrix for air pollutants S12

Supplement Table S4 – Air pollution and left ventricular diastolic average wall thickness S13

Supplement Table S5 – Air pollution and cardiovascular outcomes S14

Supplement Table S6 – Air pollution and cardiovascular outcomes, stratified by age S16

Supplement Table S7 – Air pollution and cardiovascular outcomes, stratified by sex S19

Supplement Table S9 – Air pollution and cardiovascular outcomes, stratified by BMI S26

Supplement Table S10 – Air pollution and cardiovascular outcomes, stratified by hs-CRP S29

Supplement Table S11 – Air pollution and cardiovascular outcomes, stratified by hypertension S32

Supplement Table S12 – Air pollution and adipose tissue outcomes S35

Supplement Table S13 – Air pollution and adipose tissue outcomes, stratified by age S37

Supplement Table S14 – Air pollution and adipose tissue outcomes, stratified by sex S39

Supplement Table S15 – Air pollution and adipose tissue outcomes, stratified by diabetes status S41
Supplement Table S16 – Air pollution and adipose tissue outcomes, stratified by BMI S44
Supplement Table S17 – Air pollution and adipose tissue outcomes, stratified by hs-CRP S46
Supplement Table S18 – Air pollution and adipose tissue outcomes, stratified by hypertension 48
Supplement Table S19 – Two-pollutant models and cardiovascular outcomes S50
Supplement Table S20 – Two-pollutant models and adipose tissue outcomes S57

Supplement S1 – MRI measurements

Left ventricular wall thickness: Cardiac function and morphology were assessed using cine steady-state free precession (cine-SSFP) sequences with ECG gating, capturing 10 layers and 25 phases per cardiac cycle in short-axis and 4-chamber views. Key imaging parameters included an echo time of 1.46 ms, repetition time of 29.97 ms, in-plane voxel size of 1.5 × 1.5 mm, flip angle ranging from 62 to 63°, field-of-view measuring 297 × 360 mm, matrix size of 240 × 160 mm, and a slice thickness of 8 mm.

The cine-SSFP sequences were analyzed using semi-automated analysis using cvi42 software (Circle Cardiovascular Imaging, Calgary, Canada) by two blinded, independent readers. They manually selected the left ventricular (LV) apex and base, and automatic border detection was applied to the LV endocardial and epicardial borders in short-axis views and corrected manually, if necessary.

Inter-observer and intra-observer agreement were assessed. The agreement levels were quantified using the Intraclass Correlation Coefficient (ICC).

Mean wall thickness was determined at end-diastole following the American Heart Association's 16-segment model. Segment measurements were depicted in a polar plot based on glycemic status. For subsequent statistical analysis, segments were grouped by level (basal: AHA segments 1–6; mid-cavity: AHA segments 7–12; apical: AHA segments 12–16) and region (lateral: AHA segments 5, 6, 11, 12, and 16; septal: AHA segments 2, 3, 8, and 9; anterior: AHA segments 1, 7, and 13; inferior: AHA segments 4, 10, and 15).

Left ventricular function: Cine-steady-state free precession sequences were analyzed semi-automatically with commercial software (cvi42; Circle Cardiovascular Imaging, Calgary, Alberta, Canada) by two blinded readers independently. Initially, the LV endocardium was automatically detected and manually adjusted to ensure accuracy, following established guidelines. Inter-reader variability was minimal.

Filling and ejection rates: LV volume changes were quantified using in-house software, calculating gradients and time lags. This software displays LV volume over time, its rate of change, and identifies peak gradients during early passive LV filling and late filling due to atrial contraction.

Body adipose tissue: Using the volume-interpolated three-dimensional in/opposed-phase volumetric interpolated breath-hold examination–Dixon sequence, a fat-selective tomogram was computed (5 mm slice thickness at 5 mm increment). An in-house algorithm developed with Matlab R2013a was employed for the semi-automatic quantification of adipose tissue spanning from the femoral head to the cardiac apex, visceral fat extending from the femoral head to the diaphragm, and subcutaneous adipose tissue from the femoral head to the cardiac apex. Manual adjustments were made to all segmentations as needed.

Liver fat: We utilized a modified single-voxel spectroscopy sequence (STEAM) with high-speed T2-corrected multi-echo (HISTO) for 1H Magnetic Resonance Spectroscopy (MRS). The sequence parameters included a TR of 3000 ms, a 10 ms mixing time between the second and third radiofrequency pulses, and five TEs (12.00 ms, 24.00 ms, 36.00 ms, 48.00 ms, and 72.00 ms). We acquired 1024 points within a 1200 Hz bandwidth, using a 30 x 30 x 30 mm3 voxel size, placed in the right (segment VIII) and left (segment II) liver lobes. The sequence took about 15 seconds in a single breath-hold. Automatic post-processing and lipid content estimation were performed based on previously described principles (ref. 36). The mean liver fat signal fraction was calculated from measurements in both lobes.

Pancreatic fat: For pancreatic fat content assessment (proton-density fat fraction [PDFFpanc]), circular regions of interest (ROI) measuring about 100 mm2 were drawn in the pancreatic head, body, and tail across MRI slices in a 3D multi-echo Dixon sequence of the upper abdomen. A dedicated offline workstation (Syngo Via, Siemens Healthineers, Erlangen, Germany) was used for this. Any images with significant artifacts were excluded. Variability was assessed in 40 subjects, showing low intra- and inter-reader variability.

Further information regarding the measurements can be found in Rospleszcz, S. et al. (2018). Association of glycemic status and segmental left ventricular wall thickness in subjects without prior cardiovascular disease: a cross-sectional study. BMC cardiovascular disorders, 18(1), 162. Bamberg, F. et al. (2017). Subclinical Disease Burden as Assessed by Whole-Body MRI in Subjects With Prediabetes, Subjects With

Diabetes, and Normal Control Subjects From the General Population: The KORA-MRI Study. Diabetes, 66(1), 158–169. Bayerl, C. et al. (2018). Alcohol consumption, but not smoking is associated with higher MR-derived liver fat in an asymptomatic study population. PloS one, 13(2), e0192448. Heber, S. D., Hetterich, H., Lorbeer, R., Bayerl, C., Machann, J., Auweter, S., Storz, C., Schlett, C. L., Nikolaou, K., Reiser, M., Peters, A., & Bamberg, F. (2017). Pancreatic fat content by magnetic resonance imaging in subjects with prediabetes, diabetes, and controls from a general population without cardiovascular disease. PloS one, 12(5), e0177154.

Supplement S2 – Sequential covariate models.

We conducted a multi-step approach to select the covariate models:

1. Define a pool of covariates due to disjunctive cause criterion (VanderWeele et al 2011). Covariates have been considered if they are 1) cause of the exposure, or 2) cause of the outcome, or 3) both.
2. Assign covariates to different blocks by topic. E.g. biological related covariates (BMI, waist-to-hip ratio, height, etc), social related covariates (socio-economic status, education, etc.), lifestyle covariates (smoking, physical activity, etc).
3. Check for missings, missing rates >5% are considered as problematic. Not applicable to any of the covariates of interest.
4. Test correlation of continuous covariates in each topic block. If covariates correlate highly with each other, select the covariate with the highest correlation with the outcome.
5. Perform multivariate analysis with the covariates for each topic block. Select only significant covariates with p-values <0.05. Continuous covariates were included as linear and smooth terms, selection via AIC.
6. Build sequential models for the covariates selected in the procedure above.

Supplement Table S1: Participant characteristics and environmental exposure according to sex

(A) PARTICIPANT CHARACTERISTICS			
	**Women** n = 169	**Men** n = 231	**Test** p-value
	**Mean *(SD)***	**Mean *(SD)***	
**Age** [years]	56.3 *(9.0)*	56.3 *(9.3)*	0.970 ([a])
**Weight** [kg]	73.7 *(14.8)*	90.0 *(14.4)*	<0.001 ([a])
**BMI** [kg/m^3]	27.7 *(5.5)*	28.5 *(4.4)*	0.11 ([a])
**Height** [cm]	163.3 *(6.5)*	177.7 *(11.3)*	<0.001 ([a])
**SBP** [mmHg]	113.0 *(14.5)*	126.2 *(16.2)*	<0.001 ([a])
**DBP** [mmHg]	72.0 *(8.5)*	77.7 *(10.3)*	<0.001 ([a])
**PP** [mmHg]	70.9 (7.7)	71.5 (11.3)	0.51 ([a])
**Cholesterol** [mg/dl]	219.8 *(34.9)*	216.6 *(37.4)*	0.39 ([a])
**HDL** [mg/dl]	70.1 *(17.3)*	55.8 *(15.1)*	<0.001 ([a])
**LDL** [mg/dl]	137.4 *(32.2)*	141.3 *(33.3)*	0.24 ([a])
**TAG** [mg/dl]	103.0 *(45.8)*	153.0 *(99.4)*	<0.001 ([a])
	**Median *(IQR)***	**Median *(IQR)***	
**Alcohol consumption** [g/day]	2.9 *(12.3)*	20.0 *(16.4)*	<0.001 ([b])
**hsCRP** [mg/l]	1.4 *(2.1)*	1.1 *(1.7)*	0.07 ([b])
	**N *(%)***	**N *(%)***	
**Diabetes status**			0.002 ([c])
Diabetes	14 *(8%)*	40 *(17%)*	
Prediabetes	37 *(22%)*	66 *(29%)*	
Normoglycemia	118 *(70%)*	125 *(54%)*	
**Antihypertensive medication**	47 *(28%)*	55 *(24%)*	0.423 ([c])
**Lipid lowering medication**	17 *(10%)*	25 *(11%)*	0.940 ([c])
missing	0 *(0%)*	0 *(0%)*	
**Antidiabetic medication**	12 *(7%)*	20 *(9%)*	0.704 ([c])
**Household income per month**			0.615 ([d])
<625€	7 *(4%)*	7 *(3%)*	
625€ to <1250€	45 *(27%)*	61 *(26%)*	
1250€ to <1875€	79 *(47%)*	113 *(49%)*	
1875€ to <2500€	6 *(4%)*	5 *(2%)*	
≥2500€	20 *(12%)*	39 *(17%)*	

missing	12 *(5%)*	6 *(3%)*	
**Marital status**			<0.001 ([d])
Unmarried, living alone	15 *(8%)*	24 *(10%)*	
Unmarried, living with the partner	8 *(5%)*	7 *(3%)*	
Married, living with the spouse	107 *(65%)*	182 *(79%)*	
Married, living apart	5 *(3%)*	4 *(2%)*	
Divorced	19 *(11%)*	12 *(5%)*	
Widowed	15 *(8%)*	2 *(1%)*	
**Years of education**			<0.001 ([d])
8	6 *(4%)*	4 *(2%)*	
10	71 *(42%)*	66 *(29%)*	
11	31 *(18%)*	22 *(10%)*	
12	11 *(7%)*	27 *(11%)*	
13	31 *(18%)*	49 *(21%)*	
15	0 *(0%)*	5 *(2%)*	
17	19 *(11%)*	58 *(25%)*	
**Smoking habits**			0.079 ([c])
Regular	35 *(21%)*	45 *(20%)*	
Former	63 *(38%)*	111 *(48%)*	
Never	70 *(42%)*	74 *(32%)*	
**Physical activity**			0.107 ([c])
Very active	47 *(28%)*	68 *(30%)*	
Moderate active	61 *(36%)*	62 *(27%)*	
Little active	25 *(15%)*	31 *(13%)*	
Non-active	35 *21%)*	69 *(30%)*	
**(B) ENVIRONMENTAL EXPOSURE**			
**PM_{10}** [µg/m^3]	16.5 *(2.1)*	16.4 *(2.0)*	0.516 ([a])
**$PM_{2.5}$** [µg/m^3]	11.7 *(1.4)*	11.7 *(1.4)*	0.776 ([a])
**PM_{coarse}** [µg/m^3]	4.8 *(1.3)*	4.8 *(1.5)*	0.566 ([a])
**PNC** [n/m^3]	7,045.2 (2,140.1*)*	7,124.1 (2,320.1*)*	0.695 ([a])
**NO_2** [µg/m^3]	13.6 *(6.1)*	13.7 *(5.8)*	0.866 ([a])
**NO_X** [µg/m^3]	20.8 *(9.6)*	21.4 *(9.5)*	0.439 ([a])
**PM25abs** [10^{-5} m^{-1}]	1.2 *(0.3)*	1.2 *(0.3)*	0.787 ([a])

Table S1: Participant characteristics and environmental exposure according to sex. SD: standard deviation; IQR: interquartile range; SBP: systolic blood pressure in mmHg; DBP: diastolic blood pressure in mmHg; PP: pulse pressure

in mmHg; BMI: Body mass index; WHR: Waist-to-hip ratio; HDL: High densitiy lipoprotein; LDL: Low density lipoprotein; TAG: Triacylglycerides; hsCRP: high sensitive c-reactive protein. PM_{10}: particulate matter with an aerodynamic diameter ≥ 10μm. $PM_{2.5}$: particulate matter with an aerodynamic diameter ≥ 2.5μm. PM_{coarse}: particles with an aerodynamic parameter 10-2.5 μm. PNC: particle number concentration. NO_2: nitrogen dioxide. NO_X: nitrogen oxides. PM_{25abs}: $PM_{2.5}$ absorbance.

t-Test: ([a]); Wilcoxon-Rank-Test for non-normal distributed variables: ([b]); Chi-Square-Test: ([c]); Fishers Exact Test: ([d]).

Supplement Table S2: Outcome description according to sex

CARDIOVASCULAR SYSTEM	Mean *(SD)*	Mean *(SD)*	Test
**LEFT VENTRICLE**	**Women** n = 161	**Men** n = 218	
End-diastolic volume [ml]	116.4 (27.0)	138.5 (33.8)	**
End-systolic volume [ml]	34.4 (15.1)	45.5 (18.8)	**
Stroke volume [ml]	82.0 (18.2)	93.1 (21.3)	**
Ejection fraction [%]	71.0 (7.9)	67.8 (8.1)	**
Diastolic myocardial mass [g]	115.1 (26.0)	159.7 (28.3)	**
Diastolic average wall thickness – basal AHA segments [mm]	9.0 (1.4)	10.7 (1.5)	**
Diastolic average wall thickness – mid AHA segments [mm]	8.5 (1.6)	10.4 (1.6)	**
Diastolic average wall thickness – apical AHA segments [mm]	7.7 (1.4)	8.9 (1.4)	**
Diastolic average wall thickness – lateral AHA segments [mm]	8.9 (1.6)	10.4 (1.4)	**
Diastolic average wall thickness – septal AHA segments [mm]	8.4 (1.4)	10.3 (1.5)	**
Diastolic average wall thickness – anterior AHA segments [mm]	8.4 (1.6)	10.1 (1.7)	**
Diastolic average wall thickness – inferior AHA segments [mm]	8.5 (1.4)	10.1 (1.3)	**
Diastolic average wall thickness – global AHA segments [mm]	8.6 (1.3)	10.1 (1.3)	**
Left ventricular remodeling [g/ml]	1.0 (0.3)	1.2 (0.3)	**
**RIGHT VENTRICLE**	**Women** n = 143	**Men** n = 194	
End-diastolic volume [ml]	144.3 (31.0)	181.0 (38.5)	**
End-systolic volume [ml]	64.1 (19.0)	90.1 (24.7)	**
Stroke volume [ml]	80.3 (16.9)	90.9 (20.1)	**
Ejection fraction [%]	60.9 (6.1)	50.5 (6.7)	**
**VESSLES**	**Women** n = 161	**Men** n = 210	
Diameter ascending Aorta [cm]	3.2 (0.6)	3.4 (0.4)	**
Maximum diameter infrarenal Aorta [cm]	1.4 (0.2)	1.6 (0.2)	**
Diameter pulmonary trunc [cm]	2.7 (0.4)	2.7 (0.3)	
Diameter right pulmonary artery [cm]	1.8 (0.3)	1.9 (0.3)	**
Diameter left pulmonary artery [cm]	1.9 (0.2)	2.0 (0.2)	**
**ADIPOSE TISSUE**			

**WHOLE BODY ADIPOSE TISSUE**	**Women**<br>n = 161	**Men**<br>n = 223	
Total adipose tissue [l]	12.0 (5.8)	13.0 (5.2)	
Visceral adipose tissue [l]	3.0 (2.0)	5.6 (2.6)	**
Subcutaneous adipose tissue [l]	9.1 (4.2)	7.4 (3.2)	**
**ORGAN ADIPOSE TISSUE**	**Women**<br>n = 138	**Men**<br>n = 203	
Epi – and pericardial adipose tissue [ml]	98.1 (56.8)	152.2 (75.3)	**
Systolic epicardial adipose tissue [cm^2]	7.3 (3.7)	10.0 (4.8)	**
Systolic pericardial adipose tissue [cm^2]	20.9 (10.3)	35.9 (17.2)	**
Diastolic epicardial adipose tissue [cm^2]	6.5 (3.5)	9.2 (4.5)	**
Diastolic pericardial adipose tissue [cm^2]	18.5 (9.5)	32.8 (15.9)	**
	**Women**<br>n = 160	**Men**<br>n = 224	
Mean adipose tissue content of the liver [%]	6.3 (6.3)	10.7 (8.7)	**
Mean adipose tissue content of the pancreas [%]	6.3 (5.2)	8.8 (7.8)	**
	**Women**<br>n = 158	**Men**<br>n = 208	
Renal hilus adipose tissue [ml]	29.6 (13.4)	47.9 (17.0)	**

Supplement Table S2: SD: standard deviation; IQR: interquartile range; [unit]; t-Test: ** when p-value <0.05.

Supplement Table S3: Correlation matrix for air pollutants

	PM_{10}	PM_{25}	PM_{Coarse}	PNC	NO_2	NO_X	PM_{25}abs
**PM_{10}**	1	0.50	0.79	0.82	0.76	0.75	0.80
**PM_{25}**		1	0.53	0.62	0.70	0.75	0.60
**PM_{Coarse}**			1	0.76	0.81	0.73	0.77
**PNC**				1	0.78	0.93	0.76
**NO_2**					1	0.85	0.86
**NO_X**						1	0.73
**PM_{25}abs**							1

Supplement Table S3: Correlation matrix for air pollutants with the respective Pearson correlation coefficients. PM_{10}: particulate matter with an aerodynamic diameter ≥ 10µm. $PM_{2.5}$: particulate matter with an aerodynamic diameter ≥ 2.5µm. PM_{coarse}: particles with an aerodynamic parameter 10-2.5 µm. PNC: particle number concentration. NO_2: nitrogen dioxide. NO_X: nitrogen oxides. PM_{25abs}: $PM_{2.5}$ absorbance.

Supplement Table S4 – Air pollution and left ventricular diastolic average wall thickness

Outcome	PM_{10}	PM_{coarse}	$PM_{2.5}$	PNC	NO_2	NO_x	$PM_{25}abs$
	%-change [95%-CI]	*%-change [95%-CI]*	*%-change [95%-CI]*	*%-change [95%-CI]*	*%-change [95%-CI]*	*%-change [95%-CI]*	*%-change [95%-CI]*
**Global segments**							
Basic model	-0.10 [-2.13, 1.93]	0.09 [-1.93, 2.11]	-0.70 [-2.65, 1.26]	-0.73 [-2.34, 0.88]	-1.71 [-3.63, 0.22]*	-1.28 [-3.07, 0.51]	-1.38 [-3.63, 0.87]
Main model	-0.49 [-2.40, 1.42]	0.00 [-1.91, 1.91]	-0.97 [-2.79, 0.86]	-1.03 [-2.53, 0.48]	-1.89 [-3.70, -0.09]**	-1.52 [-3.19, 0.14]*	-1.98 [-4.09, 0.13]*
**Basal segments**							
Basic model	-0.78 [-3.01, 1.44]	-0.33 [-2.54, 1.88]	-1.04 [-3.17, 1.10]	-1.15 [-2.91, 0.61]	-2.11 [-4.21, -0.01]**	-1.75 [-3.70, 0.21]*	-1.61 [-4.06, 0.85]
Main model	-1.31 [-3.45, 0.83]	-0.70 [-2.84, 1.45]	-1.43 [-3.47, 0.61]	-1.67 [-3.35, 0.02]*	-2.50 [-4.51, -0.48]**	-2.27 [-4.13, -0.41]**	-2.36 [-4.72, 0.01]*
**Mid segments**							
Basic model	0.12 [-2.31, 2.56]	0.10 [-2.32, 2.52]	-0.83 [-3.17, 1.51]	-0.72 [-2.65, 1.21]	-2.00 [-4.31, 0.30]*	-1.38 [-3.53, 0.76]	-1.92 [-4.61, 0.76]
Main model	-0.20 [-2.47, 2.07]	0.19 [-2.08, 2.46]	-1.01 [-3.18, 1.15]	-0.93 [-2.72, 0.86]	-2.10 [-4.24, 0.05]*	-1.51 [-3.49, 0.47]	-2.47 [-4.97, 0.03]
**Apical segments**							
Basic model	0.73 [-1.75, 3.21]	0.83 [-1.64, 3.30]	0.13 [-2.26, 2.52]	-0.03 [-2.00, 1.94]	-0.51 [-2.88, 1.85]	-0.30 [-2.49, 1.89]	-0.07 [-2.82, 2.68]
Main model	0.48 [-2.01, 2.97]	0.91 [-1.57, 3.40]	-0.08 [-2.46, 2.29]	-0.09 [-2.05, 1.88]	-0.52 [-2.88, 1.84]	-0.26 [-2.43, 1.92]	-0.52 [-3.27, 2.24]
**Lateral segments**							
Basic model	-0.43 [-2.56, 1.70]	-0.42 [-2.53, 1.70]	-0.68 [-2.73, 1.37]	-0.74 [-2.43, 0.94]	-1.89 [-3.91, 0.12]*	-1.28 [-3.16, 0.60]	-1.66 [-4.01, 0.69]
Main model	-0.74 [-2.78, 1.30]	-0.47 [-2.51, 1.56]	-0.85 [-2.79, 1.10]	-0.94 [-2.55, 0.66]	-1.99 [-3.91, -0.07]**	-1.43 [-3.21, 0.34]	-2.25 [-4.50, -0.01]**
**Inferior segments**							
Basic model	-0.51 [-2.66, 1.64]	0.11 [-2.02, 2.25]	-0.69 [-2.76, 1.37]	-0.74 [-2.44, 0.96]	-2.16 [-4.19, -0.13]**	-1.43 [-3.32, 0.46]	-2.38 [-4.75, -0.01]**
Main model	-0.98 [-3.05, 1.09]	-0.05 [-2.12, 2.02]	-0.93 [-2.90, 1.05]	-1.04 [-2.66, 0.59]	-2.38 [-4.33, -0.44]**	-1.63 [-3.43, 0.17]*	-3.01 [-5.28, -0.74]**
**Anterior segments**							
Basic model	0.70 [-1.92, 3.32]	0.33 [-2.28, 2.93]	-0.15 [-2.67, 2.37]	-0.48 [-2.56, 1.59]	-1.51 [-3.99, 0.98]	-1.08 [-3.40, 1.23]	-0.61 [-3.51, 2.29]
Main model	0.41 [-2.12, 2.95]	0.41 [-2.13, 2.95]	-0.44 [-2.86, 1.98]	-0.71 [-2.71, 1.29]	-1.68 [-4.08, 0.72]	-1.31 [-3.52, 0.91]	-1.12 [-3.93, 1.69]
**Septal segments**							
Basic model	-0.31 [-2.53, 1.91]	0.26 [-1.94, 2.47]	-1.39 [-3.53, 0.74]	-1.21 [-2.97, 0.55]	-1.84 [-3.95, 0.26]*	-1.72 [-3.68, 0.23]*	-1.33 [-3.79, 1.12]
Main model	-0.83 [-2.93, 1.28]	0.02 [-2.09, 2.12]	-1.74 [-3.74, 0.27]*	-1.69 [-3.34, -0.04]**	-2.19 [-4.17, -0.20]**	-2.16 [-3.98, -0.33]**	-2.02 [-4.35, 0.30]*

Table S4: Diastolic average wall thickness and long-term exposure to air pollution – percent change and confidence intervals of the outcome mean per interquartile range increase in the air pollutant of interest. Basic model: adjusted for age and sex. Main model: adjusted for age, sex, height, body mass index, income, marital status, education years and smoking. Significant association ($p > 0.05$): **. Trend ($p < 0.1$): *.

Supplement Table S5 – Air pollution and cardiovascular outcomes

Outcome	PM_{10}	PM_{coarse}	$PM_{2.5}$	PNC	NO_2	NO_x	$PM_{25}abs$
	%-change [95%-CI]	*%-change [95%-CI]*	*%-change [95%-CI]*	*%-change [95%-CI]*	*%-change [95%-CI]*	*%-change [95%-CI]*	*%-change [95%-CI]*
**LEFT VENTRICLE**							
**End-diastolic volume**							
Basic model	6.86 [-4.14, 17.86]	3.29 [-7.65, 14.23]	4.53 [-6.03, 15.09]	5.32 [-3.39, 14.03]	3.56 [-6.91, 14.03]	4.07 [-5.64, 13.77]	2.36 [-9.83, 14.55]
Main model	3.12 [-7.76, 13.99]	2.15 [-8.73, 13.03]	5.64 [-4.73, 16.00]	3.54 [-5.03, 12.12]	4.79 [-5.52, 15.09]	4.75 [-4.75, 14.26]	-0.37 [-12.42, 11.69]
**End-systolic volume**							
Basic model	1.11 [-0.86, 3.08]	0.58 [-1.38, 2.54]	0.92 [-0.97, 2.81]	1.18 [-0.38, 2.73]	0.92 [-0.95, 2.79]	0.97 [-0.77, 2.70]	0.80 [-1.38, 2.98]
Main model	0.68 [-1.35, 2.71]	0.26 [-1.77, 2.29]	1.15 [-0.78, 3.08]	1.01 [-0.59, 2.61]	1.03 [-0.90, 2.95]	1.15 [-0.62, 2.92]	0.47 [-1.78, 2.72]
**Stroke volume**							
Basic model	1.50 [-1.79, 4.78]	0.65 [-2.62, 3.91]	0.72 [-2.43, 3.88]	0.73 [-1.88, 3.33]	0.29 [-2.83, 3.42]	0.46 [-2.44, 3.35]	-0.12 [-3.76, 3.52]
Main model	0.40 [-2.85, 3.64]	0.59 [-2.66, 3.83]	0.90 [-2.19, 4.00]	0.15 [-2.41, 2.71]	0.71 [-2.37, 3.79]	0.50 [-2.33, 3.34]	-0.90 [-4.49, 2.69]
**Ejection fraction**							
Basic model	-0.18 [-1.91, 1.55]	0.04 [-1.68, 1.76]	-0.38 [-2.04, 1.28]	-0.41 [-1.78, 0.96]	-0.41 [-2.05, 1.24]	-0.36 [-1.88, 1.17]	-0.47 [-2.38, 1.44]
Main model	-0.09 [-1.89, 1.72]	0.39 [-1.42, 2.19]	-0.42 [-2.15, 1.30]	-0.35 [-1.78, 1.07]	-0.34 [-2.05, 1.38]	-0.45 [-2.02, 1.13]	-0.37 [-2.37, 1.63]
**Diastolic myocardial mass**							
Basic model	1.53 [-1.37, 4.42]	0.85 [-2.03, 3.73]	0.16 [-2.61, 2.94]	0.41 [-1.88, 2.71]	-1.17 [-3.92, 1.59]	-0.43 [-2.99, 2.12]	-1.23 [-4.43, 1.98]
Main model	0.34 [-2.30, 2.97]	0.36 [-2.27, 3.00]	-0.18 [-2.69, 2.34]	-0.40 [-2.48, 1.68]	-1.33 [-3.83, 1.17]	-0.76 [-3.07, 1.55]	-2.62 [-5.53, 0.29]*
**Left ventricular remodeling**							
Basic model	-0.40 [-4.25, 3.45]	0.02 [-3.81, 3.84]	-0.76 [-4.45, 2.93]	-1.09 [-4.14, 1.96]	-2.39 [-6.04, 1.26]	-1.55 [-4.94, 1.84]	-2.02 [-6.28, 2.24]
Main model	-0.50 [-4.27, 3.28]	0.13 [-3.64, 3.91]	-1.15 [-4.75, 2.45]	-1.19 [-4.16, 1.79]	-2.98 [-6.55, 0.58]	-1.95 [-5.25, 1.34]	-2.65 [-6.83, 1.52]
**RIGHT VENTRICLE**							
**End-diastolic volume**							
Basic model	0.14 [-3.09, 3.38]	-1.50 [-4.81, 1.82]	0.03 [-3.15, 3.21]	-0.23 [-2.89, 2.43]	-0.49 [-3.65, 2.68]	-0.46 [-3.43, 2.52]	-1.78 [-5.46, 1.91]
Main model	-1.29 [-4.40, 1.83]	-1.86 [-5.08, 1.36]	0.58 [-2.47, 3.62]	-0.94 [-3.49, 1.61]	-0.42 [-3.47, 2.63]	-0.43 [-3.27, 2.41]	-2.94 [-6.50, 0.62]
**End-systolic volume**							
Basic model	-0.65 [-4.98, 3.69]	-2.28 [-6.72, 2.16]	-1.22 [-5.48, 3.03]	-0.63 [-4.19, 2.93]	-1.45 [-5.68, 2.79]	-1.21 [-5.19, 2.77]	-2.71 [-7.65, 2.23]
Main model	-2.20 [-6.50, 2.10]	-3.16 [-7.61, 1.29]	-0.48 [-4.68, 3.73]	-1.36 [-4.89, 2.16]	-1.78 [-5.99, 2.43]	-1.14 [-5.06, 2.78]	-4.28 [-9.20, 0.64]*
**Stroke volume**							

Basic model	0.80 [-2.46, 4.07]	-0.86 [-4.21, 2.49]	1.14 [-2.06, 4.35]	0.07 [-2.61, 2.75]	0.37 [-2.82, 3.56]	0.19 [-2.81, 3.19]	-0.96 [-4.68, 2.77]
Main model	-0.49 [-3.69, 2.70]	-0.73 [-4.05, 2.58]	1.52 [-1.60, 4.64]	-0.61 [-3.22, 2.01]	0.81 [-2.32, 3.94]	0.18 [-2.74, 3.09]	-1.74 [-5.40, 1.93]
**Ejection fraction**							
Basic model	0.80 [-1.08, 2.68]	0.87 [-1.05, 2.80]	1.34 [-0.50, 3.18]	0.39 [-1.16, 1.93]	1.12 [-0.71, 2.96]	0.74 [-0.98, 2.47]	0.97 [-1.17, 3.12]
Main model	0.98 [-0.99, 2.94]	1.32 [-0.71, 3.35]	1.16 [-0.76, 3.08]	0.42 [-1.19, 2.03]	1.44 [-0.48, 3.36]	0.68 [-1.12, 2.47]	1.36 [-0.89, 3.61]
**VESSLES**							
**Diameter ascending Aorta**							
Basic model	1.43 [-0.18, 3.03]*	0.33 [-1.30, 1.95]	0.16 [-1.38, 1.69]	1.55 [0.27, 2.83]**	0.53 [-1.04, 2.09]	1.35 [-0.09, 2.78]*	0.74 [-1.05, 2.53]
Main model	1.09 [-0.57, 2.75]	0.37 [-1.35, 2.08]	0.30 [-1.28, 1.87]	1.31 [-0.02, 2.64]*	0.83 [-0.80, 2.45]	1.28 [-0.19, 2.75]*	0.87 [-1.00, 2.73]
**Maximum diameter infrarenal**							
Basic model	0.00 [-1.77, 1.77]	-1.21 [-2.99, 0.58]	-0.51 [-2.19, 1.17]	-0.14 [-1.55, 1.28]	-0.74 [-2.46, 0.97]	-0.40 [-1.98, 1.18]	-1.19 [-3.15, 0.77]
Main model	-0.43 [-2.14, 1.28]	-1.25 [-3.00, 0.51]	-0.65 [-2.27, 0.97]	-0.52 [-1.89, 0.84]	-0.77 [-2.44, 0.90]	-0.57 [-2.08, 0.95]	-1.57 [-3.49, 0.34]
**Diameter pulmonary trunk**							
Basic model	1.15 [-0.77, 3.07]	0.22 [-1.72, 2.16]	-1.49 [-3.31, 0.33]	1.00 [-0.53, 2.54]	-0.34 [-2.20, 1.53]	0.24 [-1.48, 1.95]	0.29 [-1.85, 2.42]
Main model	0.49 [-1.40, 2.39]	-0.03 [-1.98, 1.91]	-1.13 [-2.92, 0.65]	0.73 [-0.78, 2.24]	0.00 [-1.84, 1.85]	0.32 [-1.36, 2.00]	-0.27 [-2.40, 1.85]
**Diameter right pulmonary artery**							
Basic model	0.95 [-1.19, 3.09]	0.70 [-1.46, 2.87]	-0.18 [-2.21, 1.86]	0.78 [-0.93, 2.50]	0.41 [-1.67, 2.49]	0.50 [-1.41, 2.40]	1.02 [-1.36, 3.40]
Main model	0.28 [-1.89, 2.46]	0.30 [-1.94, 2.55]	-0.10 [-2.16, 1.96]	0.37 [-1.38, 2.11]	0.51 [-1.62, 2.64]	0.36 [-1.57, 2.29]	0.36 [-2.08, 2.81]
**Diameter left pulmonary artery**							
Basic model	-0.16 [-1.96, 1.65]	0.11 [-1.71, 1.93]	-1.25 [-2.95, 0.46]	0.04 [-1.40, 1.48]	-0.13 [-1.88, 1.62]	-0.51 [-2.11, 1.10]	-0.45 [-2.46, 1.55]
Main model	-0.56 [-2.37, 1.26]	0.11 [-1.76, 1.97]	-1.12 [-2.83, 0.60]	-0.13 [-1.58, 1.32]	0.17 [-1.60, 1.94]	-0.35 [-1.96, 1.25]	-0.64 [-2.68, 1.39]

Table S5: Cardiovascular outcomes and long-term exposure to air pollution – percent change and confidence intervals of the outcome mean per interquartile range increase in the air pollutant of interest. Basic model: adjusted for age and sex. Main model: adjusted for age, sex, height, body mass index, income, marital status, education years and smoking. Significant association ($p > 0.05$): **. Trend ($p < 0.1$): *.

Supplement Table S6 – Air pollution and cardiovascular outcomes, stratified by age

Outcome	PM_{10}	PM_{coarse}	$PM_{2.5}$	PNC	NO_2	NO_x	$PM_{25}abs$
	%-change [95%-CI]	*%-change [95%-CI]*	*%-change [95%-CI]*	*%-change [95%-CI]*	*%-change [95%-CI]*	*%-change [95%-CI]*	*%-change [95%-CI]*
**LEFT VENTRICLE**							
**End-diastolic volume**							
Age <65 years	1.49 [-2.3, 5.28]	0.76 [-3.03, 4.54]	2.58 [-1.1, 6.27]	1.31 [-1.68, 4.31]	1.04 [-2.63, 4.7]	1.33 [-1.98, 4.64]	0.42 [-3.86, 4.71]
Age ≥65 years	0.61 [-8.43, 9.65]	1.96 [-7.02, 10.94]	-0.82 [-9.3, 7.65]	1.27 [-5.87, 8.41]	4.64 [-3.34, 12.61]	3.74 [-4.5, 11.97]	-1.25 [-11.02, 8.51]
**End-systolic volume**							
Age <65 years	2.28 [-5.22, 9.78]	0.33 [-7.15, 7.81]	4.14 [-3.15, 11.42]	3.25 [-2.68, 9.17]	2.09 [-5.16, 9.34]	3.19 [-3.35, 9.72]	1.39 [-7.08, 9.86]
Age ≥65 years	4.89 [-8, 17.77]	5.62 [-7.17, 18.41]	0.96 [-11.17, 13.09]	2.81 [-7.4, 13.01]	8.71 [-2.62, 20.04]	6.02 [-5.75, 17.78]	1.74 [-12.24, 15.71]
**Stroke volume**							
Age <65 years	1.09 [-2.41, 4.6]	0.94 [-2.55, 4.44]	1.87 [-1.53, 5.28]	0.42 [-2.35, 3.19]	0.58 [-2.81, 3.96]	0.47 [-2.59, 3.53]	0 [-3.96, 3.95]
Age ≥65 years	-1.49 [-10.62, 7.64]	0.21 [-8.88, 9.29]	-1.67 [-10.23, 6.88]	0.53 [-6.69, 7.75]	2.69 [-5.42, 10.81]	2.66 [-5.69, 11.01]	-2.81 [-12.65, 7.04]
**Ejection fraction**							
Age <65 years	0.03 [-2.07, 2.14]	0.62 [-1.47, 2.72]	-0.38 [-2.43, 1.66]	-0.25 [-1.91, 1.41]	-0.1 [-2.13, 1.93]	-0.33 [-2.17, 1.5]	-0.27 [-2.64, 2.1]
Age ≥65 years	-0.97 [-4.7, 2.76]	-0.78 [-4.49, 2.93]	-0.27 [-3.78, 3.23]	-0.07 [-3.03, 2.88]	-0.97 [-4.3, 2.36]	-0.39 [-3.82, 3.04]	-0.14 [-4.18, 3.9]
**Diastolic myocardial mass**							
Age <65 years	1.4 [-1.3, 4.11]	1.71 [-0.99, 4.41]	1.8 [-0.83, 4.43]	0.5 [-1.64, 2.65]	-0.27 [-2.89, 2.35]	0.17 [-2.2, 2.54]	-1.17 [-4.23, 1.89]
Age ≥65 years	-3.59 [-11.3, 4.12]	-4.24 [-11.88, 3.4]	-5.6 [-12.73, 1.54]	-2.64 [-8.74, 3.45]	-3.78 [-10.63, 3.07]	-3.42 [-10.48, 3.64]	-4.01 [-12.33, 4.31]
**Left ventricular remodeling**							
Age <65 years	0.0 [-0.46, 0.46]	0.0 [-0.45, 0.45]	0.18 [-0.29, 0.64]	-0.04 [-0.41, 0.32]	-0.12 [-0.56, 0.33]	-0.06 [-0.47, 0.34]	-0.21 [-0.73, 0.32]
Age ≥65 years	-0.64 [-1.93, 0.65]	-0.56 [-1.76, 0.64]	-0.85 [-2.12, 0.41]	-0.63 [-1.64, 0.37]	-1.27 [-2.38, -0.16]**	-1.14 [-2.29, 0]	-0.39 [-1.77, 0.99]
**Global segments**							
Age <65 years	0.04 [-1.99, 2.07]	0.88 [-1.14, 2.9]	0.1 [-1.87, 2.08]	-0.45 [-2.05, 1.16]	-1 [-2.96, 0.96]	-0.71 [-2.48, 1.06]	-1.41 [-3.7, 0.87]
Age ≥65 years	-3.66 [-8.9, 1.58]	-4.29 [-9.47, 0.88]	-4.08 [-8.96, 0.8]	-3.04 [-7.18, 1.09]	-5.16 [-9.72, -0.61]**	-5.16 [-9.86, -0.46]**	-2 [-7.72, 3.73]
**Basal segments**							
Age <65 years	-0.82 [-3.17, 1.54]	0.16 [-2.19, 2.5]	-0.09 [-2.38, 2.2]	-1.18 [-3.04, 0.68]	-1.76 [-4.03, 0.5]	-1.54 [-3.59, 0.5]	-2.12 [-4.76, 0.52]
Age ≥65 years	-3.92 [-9.51, 1.68]	-4.65 [-10.17, 0.87]	-6.33 [-11.4, -1.25]**	-3.49 [-7.9, 0.91]	-5.22 [-10.11, -0.33]	-6.02 [-11, -1.04]**	-1.54 [-7.67, 4.58]

**Mid segments**							
Age <65 years	0.24 [-2.11, 2.58]	1.02 [-1.31, 3.36]	-0.14 [-2.43, 2.14]	-0.36 [-2.22, 1.5]	-1.01 [-3.27, 1.26]	-0.62 [-2.67, 1.42]	-1.7 [-4.34, 0.94]
Age ≥65 years	-2.83 [-9.36, 3.71]	-3.82 [-10.28, 2.65]	-2.6 [-8.73, 3.52]	-2.78 [-7.93, 2.37]	-5.83 [-11.5, -0.16]**	-5.07 [-10.95, 0.82]	-2.59 [-9.66, 4.48]
**Apical segments**							
Age <65 years	1.25 [-1.4, 3.89]	1.94 [-0.68, 4.57]	0.88 [-1.7, 3.45]	0.7 [-1.4, 2.79]	0.35 [-2.21, 2.91]	0.63 [-1.68, 2.93]	0.31 [-2.68, 3.29]
Age ≥65 years	-4.71 [-11.68, 2.25]	-4.58 [-11.51, 2.35]	-2.7 [-9.28, 3.89]	-2.75 [-8.29, 2.79]	-4.05 [-10.27, 2.16]	-3.91 [-10.31, 2.49]	-1.83 [-9.45, 5.79]
**Lateral segments**							
Age <65 years	0.22 [-1.93, 2.38]	0.75 [-1.4, 2.89]	0.7 [-1.39, 2.79]	-0.28 [-1.98, 1.42]	-0.59 [-2.67, 1.49]	-0.38 [-2.26, 1.50]	-1.17 [-3.6, 1.26]
Age ≥65 years	-5.69 [-11.29, -0.09]**	-6.17 [-11.7, -0.64]**	-5.36 [-10.61, -0.12]**	-3.4 [-7.88, 1.09]	-7.07 [-11.9, -2.24]**	-6.07 [-11.14, -1.00]**	-4.22 [-10.37, 1.94]
**Inferior segments**							
Age <65 years	-0.77 [-3.02, 1.47]	0.49 [-1.75, 2.72]	-0.27 [-2.46, 1.91]	-0.55 [-2.32, 1.23]	-2.06 [-4.22, 0.09]	-1.04 [-3.00, 0.91]	-2.89 [-5.4, -0.38]**
Age ≥65 years	-2.29 [-7.56, 2.98]	-2.16 [-7.41, 3.08]	-2.5 [-7.43, 2.43]	-2.07 [-6.22, 2.09]	-3.12 [-7.78, 1.54]	-3.61 [-8.38, 1.16]	-0.97 [-6.69, 4.76]
**Anterior segments**							
Age <65 years	0.95 [-1.72, 3.61]	1.44 [-1.21, 4.09]	0.39 [-2.21, 2.99]	-0.26 [-2.37, 1.85]	-0.72 [-3.29, 1.86]	-0.61 [-2.94, 1.71]	-0.61 [-3.61, 2.4]
Age ≥65 years	-3.58 [-10.97, 3.8]	-5.26 [-12.53, 2.02]	-2.62 [-9.56, 4.32]	-2.61 [-8.46, 3.23]	-5.82 [-12.28, 0.65]	-4.83 [-11.54, 1.88]	-1.22 [-9.26, 6.81]
**Septal segments**							
Age <65 years	1.25 [-1.4, 3.89]	1.94 [-0.68, 4.57]	0.88 [-1.7, 3.45]	0.7 [-1.4, 2.79]	0.35 [-2.21, 2.91]	0.63 [-1.68, 2.93]	0.31 [-2.68, 3.29]
Age ≥65 years	-4.71 [-11.68, 2.25]	-4.58 [-11.51, 2.35]	-2.7 [-9.28, 3.89]	-2.75 [-8.29, 2.79]	-4.05 [-10.27, 2.16]	-3.91 [-10.31, 2.49]	-1.83 [-9.45, 5.79]
**RIGHT VENTRICLE**							
**End-diastolic volume**							
Age <65 years	-0.67 [-4.07, 2.73]	-1.6 [-5.09, 1.88]	1.6 [-1.82, 5.01]	-0.59 [-3.37, 2.19]	-0.56 [-3.94, 2.82]	-0.22 [-3.32, 2.88]	-2.29 [-6.26, 1.67]
Age ≥65 years	-2.64 [-10.87, 5.58]	-0.9 [-9.65, 7.84]	-1.53 [-9.41, 6.36]	-1.44 [-8.22, 5.34]	1.22 [-6.26, 8.7]	0.14 [-7.56, 7.85]	-3.45 [-12.7, 5.81]
**End-systolic volume**							
Age <65 years	-1.72 [-6.51, 3.08]	-2.98 [-7.9, 1.95]	0.52 [-4.31, 5.35]	-1.17 [-5.1, 2.76]	-1.92 [-6.68, 2.85]	-0.94 [-5.32, 3.44]	-3.91 [-9.51, 1.68]
Age ≥65 years	-2.65 [-13.12, 7.82]	-1.32 [-12.43, 9.79]	-2.51 [-12.52, 7.49]	-1.15 [-9.78, 7.48]	-0.14 [-9.66, 9.38]	-0.24 [-10.03, 9.55]	-3.04 [-14.84, 8.76]
**Stroke volume**							
Age <65 years	0.19 [-3.2, 3.58]	-0.48 [-3.97, 3.01]	2.52 [-0.89, 5.92]	-0.14 [-2.92, 2.65]	0.62 [-2.76, 3.99]	0.37 [-2.72, 3.47]	-0.89 [-4.86, 3.07]
Age ≥65 years	-2.52 [-12, 6.96]	-0.38 [-10.44, 9.69]	-0.48 [-9.57, 8.6]	-1.65 [-9.46, 6.15]	2.64 [-5.95, 11.22]	0.59 [-8.27, 9.46]	-3.63 [-14.3, 7.03]

**Ejection fraction**							
Age <65 years	1.07 [-1.07, 3.2]	1.33 [-0.86, 3.52]	1.2 [-0.94, 3.35]	0.57 [-1.18, 2.32]	1.44 [-0.68, 3.56]	0.71 [-1.24, 2.65]	1.61 [-0.89, 4.10]
Age ≥65 years	0.69 [-4.83, 6.21]	1.15 [-4.69, 6.98]	0.9 [-4.37, 6.17]	0.00 [-4.54, 4.54]	1.65 [-3.33, 6.63]	0.46 [-4.68, 5.61]	0.02 [-6.2, 6.24]
**VESSLES**							
**Diameter ascending Aorta**							
Age <65 years	0.96 [-0.83, 2.74]	-0.01 [-1.85, 1.83]	0.6 [-1.19, 2.39]	1.07 [-0.35, 2.5]	0.59 [-1.2, 2.38]	1.08 [-0.51, 2.68]	0.84 [-1.22, 2.89]
Age ≥65 years	2.48 [-2.16, 7.13]	2.16 [-2.55, 6.87]	0.62 [-3.39, 4.64]	3.72 [-0.14, 7.58]	2.49 [-1.67, 6.66]	3.73 [-0.5, 7.96]	2.8 [-2.33, 7.93]
**Maximum diameter infrarenal**							
Age <65 years	-0.65 [-2.56, 1.27]	-1.27 [-3.23, 0.69]	-0.52 [-2.44, 1.39]	-0.45 [-1.98, 1.08]	-0.89 [-2.81, 1.02]	-0.31 [-2.02, 1.39]	-1.8 [-3.99, 0.39]
Age ≥65 years	0.77 [-3.61, 5.16]	-1.43 [-5.85, 2.99]	-0.47 [-4.23, 3.28]	-0.15 [-3.87, 3.57]	-0.26 [-4.19, 3.68]	-1.37 [-5.4, 2.67]	0.17 [-4.68, 5.01]
**Diameter pulmonary trunk**							
Age <65 years	1.57 [-0.41, 3.55]	0.99 [-1.05, 3.02]	0.22 [-1.77, 2.20]	1.56 [-0.02, 3.14]	1.37 [-0.61, 3.35]	1.36 [-0.41, 3.12]	1.14 [-1.13, 3.42]
Age ≥65 years	-2.6 [-8.29, 3.08]	-3.69 [-9.4, 2.02]	-5.00 [-9.74, - 0.27]**	-2.08 [-6.89, 2.74]	-3.68 [-8.73, 1.37]	-3.49 [-8.7, 1.73]	-3.27 [-9.54, 3.00]
**Diameter right pulmonary artery**							
Age <65 years	0.99 [-1.33, 3.31]	1.4 [-0.98, 3.78]	1.37 [-0.95, 3.69]	1.05 [-0.81, 2.9]	1.6 [-0.71, 3.92]	1.09 [-0.98, 3.15]	1.85 [-0.81, 4.5]
Age ≥65 years	-4.46 [-10.18, 1.26]	-5.73 [-11.44, - 0.02]**	-4.2 [-9.08, 0.68]	-3.91 [-8.74, 0.92]	-4.76 [-9.85, 0.34]	-3.93 [-9.22, 1.37]	-6.21 [-12.46, 0.03]
**Diameter left pulmonary artery**							
Age <65 years	-0.2 [-2.09, 1.68]	0.41 [-1.52, 2.34]	-0.67 [-2.55, 1.21]	0.02 [-1.49, 1.52]	0.65 [-1.23, 2.54]	-0.12 [-1.8, 1.56]	-0.13 [-2.29, 2.03]
Age ≥65 years	-1.46 [-6.78, 3.87]	-0.34 [-5.73, 5.06]	-2.01 [-6.55, 2.54]	-0.19 [-4.72, 4.35]	-0.58 [-5.37, 4.21]	-0.22 [-5.15, 4.72]	-0.96 [-6.86, 4.93]

Table S5: Cardiovascular outcomes and long-term exposure to air pollution, stratified by age – percent change and confidence intervals of the outcome mean per interquartile range increase in the air pollutant of interest. Main model: adjusted for sex, height, body mass index, income, marital status, education years and smoking. Significant results with p-value <0.05 are marked with **.

Supplement Table S7 – Air pollution and cardiovascular outcomes, stratified by sex

Outcome	PM_{10}	PM_{coarse}	$PM_{2.5}$	PNC	NO_2	NO_x	$PM_{25}abs$
	%-change [95%-CI]	*%-change [95%-CI]*	*%-change [95%-CI]*	*%-change [95%-CI]*	*%-change [95%-CI]*	*%-change [95%-CI]*	*%-change [95%-CI]*
**LEFT VENTRICLE**							
**End-diastolic volume**							
Female	4.65 [0.63, 8.66]**	4.24 [-0.12, 8.6]	3.61 [-0.73, 7.96]	3.66 [0.56, 6.76]**	4.67 [0.53, 8.8]**	3.73 [0.19, 7.28]**	3.93 [-0.87, 8.74]
Male	-1.63 [-7.03, 3.78]	-1.48 [-6.5, 3.54]	1.02 [-3.7, 5.74]	-1.06 [-5.41, 3.3]	-0.13 [-4.96, 4.69]	0.07 [-4.65, 4.78]	-2.51 [-8.22, 3.2]
**End-systolic volume**							
Female	10.19 [2.52, 17.87]**	7.91 [-0.48, 16.31]	9.13 [0.84, 17.43]**	7.69 [1.76, 13.63]**	9.21 [1.27, 17.16]**	7.38 [0.58, 14.19]**	7.66 [-1.57, 16.9]
Male	-4.02 [-13.96, 5.92]	-3.92 [-13.16, 5.31]	0.02 [-8.68, 8.71]	-0.84 [-8.86, 7.18]	-0.71 [-9.6, 8.17]	0.93 [-7.75, 9.61]	-2.87 [-13.4, 7.65]
**Stroke volume**							
Female	2.02 [-2.1, 6.14]	2.49 [-1.96, 6.93]	0.99 [-3.44, 5.42]	1.74 [-1.44, 4.92]	2.56 [-1.67, 6.79]	2 [-1.62, 5.62]	2.17 [-2.72, 7.05]
Male	-0.57 [-5.54, 4.41]	-0.36 [-4.99, 4.26]	1.48 [-2.86, 5.82]	-1.14 [-5.15, 2.87]	0.12 [-4.32, 4.56]	-0.32 [-4.66, 4.02]	-2.39 [-7.64, 2.86]
**Ejection fraction**							
Female	-1.43 [-3.96, 1.09]	-0.23 [-2.97, 2.51]	-1.57 [-4.27, 1.14]	-0.73 [-2.69, 1.22]	-0.99 [-3.59, 1.62]	-0.7 [-2.93, 1.53]	-0.81 [-3.82, 2.19]
Male	0.99 [-1.59, 3.56]	0.92 [-1.48, 3.31]	0.62 [-1.63, 2.88]	0.07 [-2.01, 2.15]	0.34 [-1.96, 2.64]	-0.11 [-2.36, 2.14]	0.18 [-2.55, 2.91]
**Diastolic myocardial mass**							
Female	2.45 [-0.81, 5.72]	2.26 [-1.27, 5.8]	-0.5 [-4.03, 3.03]	1.6 [-0.93, 4.13]	0.14 [-3.24, 3.53]	0.23 [-2.67, 3.12]	-1.65 [-5.54, 2.24]
Male	-0.66 [-4.71, 3.39]	-0.85 [-4.61, 2.91]	0.55 [-2.99, 4.09]	-1.82 [-5.07, 1.44]	-1.9 [-5.5, 1.71]	-1.11 [-4.64, 2.42]	-2.49 [-6.76, 1.78]
**Left ventricular remodeling**							
Female	0.18 [-0.68, 1.04]	-0.16 [-0.75, 0.44]	-0.38 [-0.97, 0.2]	-0.18 [-0.6, 0.25]	-0.52 [-1.08, 0.04]	-0.35 [-0.83, 0.13]	-0.66 [-1.3, -0.02]
Male	-0.18 [-0.72, 0.37]	0.1 [-0.53, 0.73]	0.04 [-0.55, 0.63]	-0.10 [-0.65, 0.45]	-0.25 [-0.86, 0.35]	-0.15 [-0.74, 0.44]	-0.05 [-0.77, 0.67]
**Global segments**							
Female	-0.66 [-3.3, 1.97]	-0.66 [-3.5, 2.19]	-2.11 [-4.92, 0.7]	-0.85 [-2.88, 1.18]	-2.61 [-5.29, 0.07]	-1.98 [-4.28, 0.32]	-3.47 [-6.54, -0.40]**
Male	0.16 [-2.63, 2.95]	0.42 [-2.16, 3.01]	0.03 [-2.4, 2.46]	-0.95 [-3.19, 1.3]	-1.22 [-3.7, 1.26]	-0.93 [-3.36, 1.49]	-0.53 [-3.47, 2.42]
**Basal segments**							
Female	-0.57 [-3.37, 2.24]	-0.95 [-3.98, 2.08]	-1.69 [-4.68, 1.31]	-0.93 [-3.09, 1.23]	-2.39 [-5.25, 0.47]	-1.88 [-4.33, 0.57]	-3.05 [-6.34, 0.24]
Male	-1.41 [-4.64, 1.83]	-0.43 [-3.44, 2.59]	-0.88 [-3.71, 1.95]	-2.03 [-4.63, 0.57]	-2.32 [-5.19, 0.56]	-2.34 [-5.15, 0.47]	-1.28 [-4.71, 2.15]
**Mid segments**							
Female	-0.35 [-3.43, 2.73]	-0.29 [-3.62, 3.04]	-2.6 [-5.88, 0.67]	-0.82 [-3.2, 1.55]	-2.68 [-5.81, 0.46]	-2.11 [-4.79, 0.58]	-3.89 [-7.49, -0.3]**

Male	0.34 [-2.99, 3.67]	0.43 [-2.66, 3.53]	0.22 [-2.69, 3.13]	-0.85 [-3.53, 1.83]	-1.59 [-4.56, 1.37]	-0.9 [-3.81, 2]	-1.21 [-4.73, 2.31]
**Apical segments**							
Female	-1.37 [-5.06, 2.31]	-0.78 [-4.77, 3.21]	-2.08 [-6.02, 1.87]	-0.78 [-3.63, 2.07]	-2.94 [-6.71, 0.83]	-2 [-5.23, 1.23]	-3.58 [-7.92, 0.76]
Male	2.65 [-0.81, 6.12]	1.94 [-1.29, 5.16]	1.34 [-1.69, 4.38]	0.82 [-1.99, 3.62]	1.34 [-1.76, 4.44]	1.5 [-1.53, 4.53]	1.97 [-1.7, 5.65]
**Lateral segments**							
Female	-0.1 [-3.07, 2.86]	-0.32 [-3.52, 2.89]	-1.48 [-4.65, 1.69]	-0.32 [-2.61, 1.97]	-1.87 [-4.91, 1.16]	-1.15 [-3.75, 1.45]	-2.9 [-6.38, 0.59]
Male	-0.9 [-3.77, 1.97]	-0.65 [-3.32, 2.01]	-0.16 [-2.67, 2.34]	-1.33 [-3.64, 0.97]	-1.97 [-4.52, 0.57]	-1.56 [-4.05, 0.93]	-1.54 [-4.57, 1.49]
**Inferior segments**							
Female	-1.04 [-4.04, 1.96]	-0.54 [-3.79, 2.7]	-1.65 [-4.87, 1.56]	-0.75 [-3.07, 1.57]	-2.82 [-5.88, 0.23]	-1.86 [-4.48, 0.77]	-4.57 [-8.05, -1.09]**
Male	-0.24 [-3.17, 2.69]	0.41 [-2.32, 3.13]	-0.11 [-2.67, 2.45]	-0.97 [-3.33, 1.39]	-1.72 [-4.32, 0.89]	-1.11 [-3.66, 1.44]	-1.28 [-4.38, 1.81]
**Anterior segments**							
Female	-1.51 [-4.94, 1.92]	-1.11 [-4.82, 2.61]	-2.87 [-6.53, 0.79]	-1.57 [-4.22, 1.07]	-3.84 [-7.32, -0.36]**	-3.05 [-6.04, -0.07]**	-4.25 [-8.27, -0.24]**
Male	2.44 [-1.29, 6.18]	1.26 [-2.22, 4.75]	1 [-2.28, 4.27]	0.24 [-2.78, 3.27]	-0.32 [-3.67, 3.03]	0.21 [-3.07, 3.48]	1.13 [-2.83, 5.1]
**Septal segments**							
Female	-0.59 [-3.23, 2.06]	-0.87 [-3.73, 1.98]	-2.82 [-5.62, -0.02]	-1.36 [-3.39, 0.67]	-2.76 [-5.45, -0.08]**	-2.6 [-4.88, -0.31]**	-3.17 [-6.26, -0.08]**
Male	-0.43 [-3.66, 2.8]	0.72 [-2.27, 3.72]	-0.55 [-3.37, 2.27]	-1.63 [-4.22, 0.97]	-1.4 [-4.28, 1.47]	-1.4 [-4.21, 1.41]	-0.5 [-3.92, 2.91]
**RIGHT VENTRICLE**							
**End-diastolic volume**							
Female	-0.41 [-4.01, 3.19]	-2.03 [-6.08, 2.02]	0.35 [-3.72, 4.43]	-0.48 [-3.48, 2.52]	0.68 [-3.21, 4.56]	0.27 [-3.19, 3.73]	-1.29 [-5.76, 3.18]
Male	-1.74 [-6.81, 3.33]	-1.54 [-6.33, 3.25]	1.19 [-3.18, 5.57]	-1.01 [-5.1, 3.08]	-0.9 [-5.46, 3.66]	-0.4 [-4.8, 4]	-3.74 [-9.14, 1.66]
**End-systolic volume**							
Female	-0.45 [-5.38, 4.47]	-3.25 [-8.78, 2.27]	0.8 [-4.77, 6.37]	-0.54 [-4.64, 3.56]	0.96 [-4.35, 6.27]	0.45 [-4.28, 5.18]	-1.68 [-7.79, 4.43]
Male	-3.51 [-10.56, 3.54]	-3 [-9.66, 3.67]	-0.78 [-6.87, 5.31]	-1.58 [-7.27, 4.11]	-3.71 [-10.03, 2.62]	-1.64 [-7.76, 4.48]	-6.18 [-13.69, 1.32]
**Stroke volume**							
Female	-0.4 [-4.24, 3.45]	-0.96 [-5.29, 3.37]	0.04 [-4.31, 4.38]	-0.42 [-3.62, 2.78]	0.48 [-3.67, 4.62]	0.13 [-3.56, 3.83]	-0.95 [-5.72, 3.82]
Male	-0.17 [-5.28, 4.95]	-0.27 [-5.1, 4.57]	2.92 [-1.46, 7.31]	-0.57 [-4.69, 3.55]	1.63 [-2.96, 6.22]	0.64 [-3.79, 5.08]	-1.51 [-6.98, 3.96]
**Ejection fraction**							
Female	0.21 [-2.41, 2.83]	1.42 [-1.53, 4.36]	0 [-2.97, 2.96]	0.25 [-1.93, 2.43]	0.00 [-2.83, 2.83]	0.02 [-2.5, 2.54]	0.59 [-2.66, 3.85]
Male	1.81 [-1.2, 4.81]	1.33 [-1.52, 4.17]	1.89 [-0.7, 4.47]	0.54 [-1.89, 2.97]	2.69 [0.01, 5.37]	1.12 [-1.49, 3.73]	2.31 [-0.9, 5.51]
**VESSLES**							

**Diameter ascending Aorta**							
Female	1.35 [-0.98, 3.69]	1.09 [-1.5, 3.69]	0.47 [-1.96, 2.91]	1.44 [-0.4, 3.28]	1.3 [-1.12, 3.71]	1.73 [-0.34, 3.79]	0.84 [-1.91, 3.6]
Male	0.82 [-1.59, 3.22]	-0.39 [-2.69, 1.91]	0.08 [-2.05, 2.21]	1.1 [-0.86, 3.06]	0.35 [-1.91, 2.6]	0.79 [-1.34, 2.93]	0.88 [-1.74, 3.49]
**Maximum diameter infrarenal**							
Female	-0.62 [-2.82, 1.57]	-0.95 [-3.38, 1.48]	0.78 [-1.49, 3.05]	-0.06 [-1.79, 1.67]	0.6 [-1.66, 2.87]	0.69 [-1.26, 2.64]	-0.66 [-3.25, 1.92]
Male	-0.27 [-2.9, 2.37]	-1.51 [-4.02, 1.01]	-1.66 [-3.97, 0.66]	-1.11 [-3.26, 1.04]	-1.75 [-4.2, 0.71]	-1.76 [-4.09, 0.56]	-2.27 [-5.12, 0.58]
**Diameter pulmonary trunk**							
Female	4.26 [1.67, 6.85]**	3.6 [0.69, 6.51]**	1.91 [-0.85, 4.67]	3.51 [1.48, 5.55]**	3.56 [0.85, 6.26]**	3.73 [1.43, 6.03]**	3.52 [0.42, 6.62]**
Male	-2.53 [-5.19, 0.14]	-2.57 [-5.11, -0.02]**	-2.69 [-5.03, -0.34]**	-1.51 [-3.69, 0.67]	-2.43 [-4.92, 0.07]	-2.37 [-4.73, -0.01]**	-2.91 [-5.8, -0.01]**
**Diameter right pulmonary artery**							
Female	1.47 [-1.73, 4.66]	1.74 [-1.8, 5.27]	0.33 [-2.99, 3.64]	1.26 [-1.26, 3.77]	0.89 [-2.41, 4.19]	1.22 [-1.62, 4.06]	1.44 [-2.32, 5.2]
Male	-1.2 [-4.26, 1.87]	-0.96 [-3.9, 1.98]	-0.63 [-3.34, 2.09]	-0.84 [-3.36, 1.67]	-0.28 [-3.16, 2.59]	-0.81 [-3.53, 1.91]	-1.05 [-4.39, 2.29]
**Diameter left pulmonary artery**							
Female	0.16 [-2.38, 2.69]	1.85 [-0.94, 4.64]	-1.29 [-3.91, 1.33]	0.66 [-1.33, 2.65]	0.52 [-2.09, 3.14]	0.13 [-2.12, 2.39]	0.64 [-2.34, 3.62]
Male	-0.96 [-3.53, 1.6]	-1.19 [-3.64, 1.26]	-0.93 [-3.19, 1.34]	-0.65 [-2.74, 1.44]	-0.25 [-2.66, 2.15]	-0.66 [-2.94, 1.61]	-1.79 [-4.57, 0.99]

Table S7: Cardiovascular outcomes and long-term exposure to air pollution, stratified by sex – percent change and confidence intervals of the outcome mean per interquartile range increase in the air pollutant of interest. Main model: adjusted for age, height, body mass index, income, marital status, education years and smoking. Significant results with p-value <0.05 are marked with **.

Supplement Table S8 – Air pollution and cardiovascular outcomes, stratified by diabetes status

Outcome	PM_{10}	PM_{coarse}	$PM_{2.5}$	PNC	NO_2	NO_x	$PM_{25}abs$
	%-change [95%-CI]	*%-change [95%-CI]*	*%-change [95%-CI]*	*%-change [95%-CI]*	*%-change [95%-CI]*	*%-change [95%-CI]*	*%-change [95%-CI]*
**LEFT VENTRICLE**							
**End-diastolic volume**							
Diabetes	11.92 [-1.43, 25.27]	6.01 [-4.98, 17.01]	11.27 [0.05, 22.49]**	9.94 [-0.8, 20.67]	8.25 [-3.62, 20.12]	13.18 [0.71, 25.64]	8.84 [-0.09, 17.76]
Prediabetes	-1.96 [-8.57, 4.65]	-0.71 [-6.66, 5.24]	-0.59 [-6.33, 5.15]	-0.68 [-6.55, 5.2]	-0.97 [-7.28, 5.33]	-0.31 [-6.07, 5.45]	-0.77 [-6.13, 4.59]
Normoglycemia	1.05 [-3.9, 6.01]	1.22 [-3.29, 5.74]	2.69 [-1.52, 6.89]	2.78 [-1.01, 6.57]	0.82 [-3.59, 5.24]	3.68 [-0.52, 7.88]	1.67 [-1.73, 5.06]
**End-systolic volume**							
Diabetes	30.13 [0.87, 59.39]**	12.64 [-11.86, 37.13]	25.25 [0.32, 50.17]**	24.7 [1.13, 48.26]**	24.98 [-0.76, 50.71]	26.21 [-1.87, 54.29]	24.63 [5.47, 43.8]**
Prediabetes	-2.08 [-14.06, 9.9]	0.58 [-10.2, 11.36]	-0.63 [-11.03, 9.76]	-0.1 [-10.75, 10.54]	2.6 [-8.8, 14.0]	0.25 [-10.19, 10.69]	0.68 [-9.03, 10.39]
Normoglycemia	0.39 [-8.79, 9.56]	-1.54 [-9.89, 6.82]	3.39 [-4.42, 11.19]	3.86 [-3.17, 10.88]	-4.49 [-12.65, 3.67]	6.32 [-1.45, 14.1]	1.23 [-5.07, 7.52]
**Stroke volume**							
Diabetes	3.26 [-8.34, 14.86]	2.89 [-6.39, 12.17]	4.7 [-5.07, 14.47]	3.05 [-6.3, 12.39]	0.42 [-9.76, 10.6]	7 [-3.8, 17.8]	1.48 [-6.38, 9.33]
Prediabetes	-2 [-8.11, 4.11]	-1.3 [-6.8, 4.2]	-0.57 [-5.87, 4.74]	-0.92 [-6.35, 4.51]	-2.63 [-8.44, 3.17]	-0.52 [-5.85, 4.81]	-1.43 [-6.38, 3.52]
Normoglycemia	1.39 [-3.58, 6.37]	2.47 [-2.05, 6.99]	2.4 [-1.83, 6.63]	2.29 [-1.52, 6.09]	3.21 [-1.2, 7.63]	2.47 [-1.76, 6.7]	1.86 [-1.55, 5.26]
**Ejection fraction**							
Diabetes	-5.39 [-13.16, 2.38]	-0.82 [-7.21, 5.57]	-2.74 [-9.47, 3.99]	-2.8 [-9.17, 3.57]	-4.06 [-10.9, 2.77]	-2.18 [-9.73, 5.37]	-3.45 [-8.72, 1.81]
Prediabetes	0.3 [-3.32, 3.91]	-0.55 [-3.8, 2.7]	-0.02 [-3.15, 3.12]	-0.15 [-3.36, 3.06]	-1.42 [-4.85, 2.01]	-0.24 [-3.39, 2.9]	-0.54 [-3.47, 2.39]
Normoglycemia	0.46 [-2.26, 3.17]	1.43 [-1.03, 3.9]	-0.1 [-2.41, 2.21]	-0.22 [-2.31, 1.86]	2.32 [-0.08, 4.72]	-0.92 [-3.23, 1.39]	0.43 [-1.43, 2.3]
**Diastolic myocardial mass**							
Diabetes	-1.14 [-10.56, 8.28]	-1.41 [-8.95, 6.12]	0.91 [-7.1, 8.92]	3.48 [-4.03, 10.99]	-0.25 [-8.49, 7.98]	8.18 [-0.31, 16.68]	2.55 [-3.76, 8.86]
Prediabetes	-2.44 [-7.97, 3.1]	1.61 [-3.38, 6.6]	-2.01 [-6.81, 2.79]	-1.54 [-6.46, 3.39]	-0.44 [-5.73, 4.85]	-4.1 [-8.86, 0.66]	-1.04 [-5.54, 3.46]
Normoglycemia	-2.43 [-6.34, 1.49]	0.41 [-3.17, 3.99]	-0.86 [-4.21, 2.49]	-0.13 [-3.15, 2.89]	1.23 [-2.27, 4.73]	0.88 [-2.47, 4.23]	0.02 [-2.68, 2.72]
**Left ventricular remodeling**							
Diabetes	-1.04 [-3.01, 0.94]	-1.04 [-2.84, 0.77]	-0.94 [-3.1, 1.22]	-0.83 [-2.35, 0.7]	-1.69 [-3.56, 0.18]	-1.02 [-2.84, 0.8]	-2.01 [-4.2, 0.18]
Prediabetes	0.18 [-0.68, 1.04]	0.32 [-0.49, 1.13]	-0.29 [-1.07, 0.49]	0.04 [-0.7, 0.77]	-0.09 [-0.87, 0.69]	0.03 [-0.77, 0.83]	0.11 [-0.79, 1.01]
Normoglycemia	0.06 [-0.45, 0.57]	0.02 [-0.5, 0.54]	-0.18 [-0.66, 0.31]	-0.11 [-0.5, 0.28]	-0.4 [-0.88, 0.08]	-0.27 [-0.7, 0.17]	-0.43 [-0.99, 0.14]
**Global segments**							

Diabetes	-4.79 [-12.35, 2.76]	-2.9 [-9.02, 3.21]	-3.86 [-10.3, 2.58]	-2.18 [-8.38, 4.02]	-2.48 [-9.19, 4.22]	-1.31 [-8.64, 6.02]	-1.76 [-6.95, 3.43]
Prediabetes	-1.91 [-5.72, 1.91]	0.27 [-3.19, 3.72]	-2.06 [-5.36, 1.24]	-1.64 [-5.03, 1.75]	-0.96 [-4.61, 2.68]	-2.99 [-6.27, 0.29]	-1.44 [-4.54, 1.65]
Normoglycemia	-2.13 [-4.89, 0.63]	0.16 [-2.37, 2.69]	-1.93 [-4.28, 0.42]	-1.44 [-3.56, 0.68]	0.18 [-2.3, 2.66]	-0.58 [-2.94, 1.79]	-0.8 [-2.7, 1.11]
**Basal segments**							
Diabetes	-4.62 [-11.89, 2.64]	-3.61 [-9.44, 2.22]	-5.81 [-11.81, 0.19]	-3.96 [-9.81, 1.89]	-3.61 [-9.99, 2.77]	-5.73 [-12.51, 1.06]	-2.61 [-7.55, 2.34]
Prediabetes	-3.28 [-8.11, 1.55]	-0.24 [-4.63, 4.15]	-3.1 [-7.27, 1.08]	-3.08 [-7.36, 1.2]	-2.48 [-7.1, 2.14]	-4.24 [-8.39, -0.09]**	-2.64 [-6.55, 1.28]
Normoglycemia	-2.51 [-5.62, 0.61]	-1.17 [-4.02, 1.67]	-2.78 [-5.42, -0.14]**	-2.52 [-4.9, -0.14]**	-0.8 [-3.59, 1.99]	-0.83 [-3.5, 1.84]	-1.7 [-3.84, 0.43]
**Mid segments**							
Diabetes	-6.16 [-15.6, 3.27]	-4.09 [-11.71, 3.52]	-3.56 [-11.69, 4.57]	-1.63 [-9.41, 6.16]	-2.98 [-11.37, 5.41]	1.69 [-7.48, 10.86]	-1.72 [-8.23, 4.78]
Prediabetes	-2.56 [-7.2, 2.07]	0.75 [-3.44, 4.94]	-2.66 [-6.66, 1.35]	-1.51 [-5.64, 2.62]	-0.28 [-4.72, 4.16]	-3.34 [-7.34, 0.66]	-0.99 [-4.76, 2.78]
Normoglycemia	-2.39 [-5.57, 0.79]	0.75 [-2.16, 3.66]	-1.79 [-4.5, 0.92]	-1.3 [-3.75, 1.15]	0.59 [-2.26, 3.43]	-0.68 [-3.41, 2.04]	-0.66 [-2.85, 1.54]
**Apical segments**							
Diabetes	-2.86 [-12.48, 6.77]	0.33 [-7.41, 8.08]	-0.98 [-9.2, 7.25]	-0.01 [-7.81, 7.8]	0.34 [-8.12, 8.79]	1.4 [-7.78, 10.57]	-0.34 [-6.87, 6.2]
Prediabetes	1.62 [-3.56, 6.79]	0.35 [-4.31, 5.01]	0.75 [-3.74, 5.24]	0.67 [-3.93, 5.27]	0.54 [-4.4, 5.47]	-0.24 [-4.75, 4.27]	-0.14 [-4.34, 4.06]
Normoglycemia	-1.05 [-4.71, 2.61]	1.54 [-1.79, 4.87]	-0.71 [-3.82, 2.41]	0.21 [-2.6, 3.02]	1.23 [-2.02, 4.49]	0.06 [-3.06, 3.18]	0.56 [-1.95, 3.07]
**Lateral segments**							
Diabetes	-7.27 [-15.1, 0.55]	-5.18 [-11.51, 1.16]	-5.74 [-12.44, 0.96]	-3.52 [-10.04, 3.01]	-4.8 [-11.79, 2.19]	-2.81 [-10.56, 4.93]	-2.92 [-8.38, 2.54]
Prediabetes	-2.01 [-6.22, 2.19]	0.1 [-3.7, 3.9]	-1.72 [-5.37, 1.92]	-0.62 [-4.37, 3.13]	-1.02 [-5.04, 2.99]	-3.31 [-6.92, 0.3]	-0.33 [-3.76, 3.09]
Normoglycemia	-2.24 [-5.19, 0.72]	-0.12 [-2.82, 2.59]	-2.12 [-4.64, 0.39]	-1.66 [-3.92, 0.61]	0.09 [-2.56, 2.74]	-0.2 [-2.73, 2.33]	-1.01 [-3.04, 1.03]
**Inferior segments**							
Diabetes	-4.55 [-11.74, 2.64]	-2.41 [-8.25, 3.42]	-2.4 [-8.6, 3.81]	-0.83 [-6.77, 5.1]	-1.54 [-7.96, 4.87]	0.00 [-6.99, 7.00]	-0.81 [-5.77, 4.16]
Prediabetes	-2.08 [-6.34, 2.19]	1.79 [-2.04, 5.62]	-2.1 [-5.79, 1.59]	-0.85 [-4.65, 2.95]	-0.65 [-4.72, 3.43]	-2.34 [-6.04, 1.35]	-0.17 [-3.65, 3.3]
Normoglycemia	-3.29 [-6.35, -0.23]	-0.44 [-3.25, 2.38]	-2.55 [-5.16, 0.06]	-1.79 [-4.15, 0.58]	-0.78 [-3.53, 1.98]	-0.5 [-3.13, 2.14]	-1.15 [-3.26, 0.97]
**Anterior segments**							
Diabetes	-6.57 [-17.14, 4.01]	-3.6 [-12.18, 4.97]	-5.82 [-14.79, 3.16]	-3.29 [-11.94, 5.37]	-2.72 [-12.13, 6.68]	-1.11 [-11.38, 9.16]	-2.89 [-10.12, 4.35]
Prediabetes	0.38 [-4.85, 5.6]	0.47 [-4.22, 5.17]	-1.13 [-5.65, 3.4]	-0.43 [-5.07, 4.21]	0.62 [-4.35, 5.6]	-1.57 [-6.11, 2.96]	-0.45 [-4.68, 3.78]
Normoglycemia	-1.65 [-5.21, 1.9]	0.97 [-2.27, 4.22]	-1.72 [-4.74, 1.31]	-1.28 [-4.01, 1.45]	1.12 [-2.05, 4.29]	-0.53 [-3.56, 2.51]	-0.39 [-2.83, 2.06]
**Septal segments**							

Diabetes	-1.88 [-9.21, 5.46]	-0.96 [-6.85, 4.94]	-2.44 [-8.65, 3.76]	-1.63 [-7.55, 4.28]	-1.1 [-7.52, 5.33]	-1.36 [-8.34, 5.62]	-0.99 [-5.95, 3.98]
Prediabetes	-4.24 [-8.91, 0.42]	-1.26 [-5.53, 3.00]	-4.15 [-8.17, -0.13]**	-5.11 [-9.18, -1.04]**	-2.87 [-7.35, 1.62]	-4.78 [-8.79, -0.77]**	-4.98 [-8.68, -1.28]**
Normoglycemia	-1.68 [-4.75, 1.39]	0.29 [-2.51, 3.09]	-1.8 [-4.41, 0.81]	-1.38 [-3.74, 0.97]	0.18 [-2.56, 2.92]	-1.22 [-3.84, 1.4]	-0.79 [-2.89, 1.32]
**RIGHT VENTRICLE**							
**End-diastolic volume**							
Diabetes	1.91 [-11.04, 14.86]	-0.02 [-11.03, 11.0]	3.44 [-7.09, 13.97]	1.97 [-7.9, 11.85]	-0.59 [-11.37, 10.2]	5.82 [-4.86, 16.5]	1.28 [-6.87, 9.43]
Prediabetes	-6.19 [-11.87, -0.51]**	-4.58 [-9.82, 0.66]	-3.42 [-8.46, 1.61]	-4.45 [-9.68, 0.78]	-4.55 [-10.11, 1.01]	-1.92 [-7.03, 3.19]	-4.93 [-9.69, -0.17]**
Normoglycemia	-0.47 [-5.33, 4.39]	-0.18 [-4.59, 4.22]	1.57 [-2.51, 5.65]	1.76 [-2.01, 5.53]	0.0 [-4.09, 4.08]	2.98 [-1.13, 7.08]	0.75 [-2.62, 4.11]
**End-systolic volume**							
Diabetes	4.37 [-12.92, 21.65]	-0.88 [-15.63, 13.87]	3.5 [-10.65, 17.64]	3.16 [-10.04, 16.37]	1.17 [-13.26, 15.6]	6.39 [-8, 20.79]	3.12 [-7.75, 13.99]
Prediabetes	-10.97 [-19.17, -2.77]**	-8.52 [-16.09, -0.94]**	-7.77 [-15.02, -0.52]**	-7.96 [-15.55, -0.38]**	-6.35 [-14.51, 1.81]	-3.85 [-11.31, 3.62]	-7.87 [-14.81, -0.93]**
Normoglycemia	-0.6 [-7.45, 6.26]	-0.13 [-6.35, 6.09]	1.82 [-3.95, 7.59]	2.02 [-3.3, 7.34]	-1.19 [-6.95, 4.57]	3.35 [-2.46, 9.15]	0.87 [-3.88, 5.62]
**Stroke volume**							
Diabetes	0.01 [-13.51, 13.52]	0.94 [-10.54, 12.42]	3.61 [-7.37, 14.59]	1.02 [-9.3, 11.33]	-2.03 [-13.25, 9.19]	5.52 [-5.66, 16.69]	-0.31 [-8.82, 8.2]
Prediabetes	-1.84 [-8.42, 4.74]	-1.12 [-7.13, 4.89]	0.5 [-5.23, 6.24]	-1.36 [-7.35, 4.63]	-3 [-9.33, 3.33]	-0.17 [-5.94, 5.6]	-2.37 [-7.85, 3.11]
Normoglycemia	-0.42 [-5.23, 4.39]	-0.32 [-4.68, 4.04]	1.31 [-2.73, 5.36]	1.48 [-2.25, 5.22]	1.01 [-3.03, 5.05]	2.55 [-1.52, 6.63]	0.58 [-2.75, 3.92]
**Ejection fraction**							
Diabetes	-0.44 [-9.17, 8.29]	2.01 [-5.37, 9.39]	1.81 [-5.29, 8.92]	-0.07 [-6.73, 6.6]	-0.44 [-7.7, 6.82]	1.28 [-6.03, 8.6]	-0.99 [-6.48, 4.49]
Prediabetes	4.08 [-0.35, 8.51]	3.72 [-0.32, 7.76]	3.92 [0.08, 7.75]**	2.88 [-1.18, 6.95]	1.78 [-2.57, 6.13]	1.48 [-2.47, 5.43]	2.47 [-1.26, 6.21]
Normoglycemia	0.19 [-2.83, 3.2]	-0.02 [-2.75, 2.72]	-0.23 [-2.77, 2.31]	-0.34 [-2.69, 2]	1.03 [-1.5, 3.56]	-0.09 [-2.66, 2.47]	-0.15 [-2.24, 1.94]
**VESSLES**							
**Diameter ascending Aorta**							
Diabetes	-1.57 [-8.79, 5.65]	-0.25 [-5.71, 5.22]	-1.49 [-7.35, 4.37]	-1.22 [-6.34, 3.91]	-1.04 [-6.66, 4.57]	-1.08 [-6.37, 4.22]	-0.87 [-5.34, 3.6]
Prediabetes	1.75 [-1.9, 5.41]	1.95 [-1.4, 5.29]	2.02 [-1.13, 5.17]	3.22 [0.04, 6.4]**	2.35 [-1.19, 5.89]	0.61 [-2.47, 3.7]	3.71 [0.71, 6.71]**
Normoglycemia	0.75 [-1.79, 3.29]	-0.09 [-2.43, 2.25]	0.77 [-1.43, 2.97]	1.04 [-0.94, 3.01]	0.64 [-1.58, 2.86]	0.82 [-1.4, 3.03]	1.03 [-0.71, 2.76]
**Maximum diameter infrarenal**							
Diabetes	-0.75 [-7.98, 6.49]	-1.14 [-6.59, 4.32]	0.47 [-5.41, 6.35]	-0.69 [-5.83, 4.44]	1.7 [-3.91, 7.3]	-1.56 [-6.84, 3.72]	0.5 [-3.98, 4.99]
Prediabetes	-2.04 [-6.71, 2.64]	-1.74 [-6.03, 2.56]	-1.55 [-5.6, 2.5]	-1.17 [-5.33, 2.99]	-1.55 [-6.1, 3.01]	-3.5 [-7.36, 0.36]	-0.78 [-4.73, 3.18]
Normoglycemia	-2.33 [-4.66, 0]	-0.99 [-3.15, 1.17]	-0.89 [-2.92, 1.15]	-0.48 [-2.31, 1.34]	-0.89 [-2.94, 1.17]	0.86 [-1.19, 2.91]	-0.83 [-2.43, 0.77]

**Diameter pulmonary trunk**							
Diabetes	-3.68 [-12.2, 4.85]	-3.08 [-9.51, 3.35]	-5.94 [-12.64, 0.77]	-6.08 [-11.84, -0.32]**	-3.13 [-9.74, 3.49]	-7.93 [-13.64, -2.23]**	-3.65 [-8.84, 1.54]
Prediabetes	-1.22 [-5.14, 2.71]	-1.62 [-5.21, 1.97]	-0.26 [-3.66, 3.15]	-0.68 [-4.17, 2.8]	-1.4 [-5.21, 2.41]	-2.04 [-5.31, 1.23]	-0.21 [-3.53, 3.11]
Normoglycemia	1.89 [-0.77, 4.56]	2.68 [0.25, 5.12]**	2.00 [-0.3, 4.31]	2.54 [0.49, 4.59]	2.17 [-0.15, 4.49]	1.62 [-0.7, 3.95]	2.52 [0.73, 4.31]**
**Diameter right pulmonary artery**							
Diabetes	-3.26 [-10.15, 3.62]	0.02 [-5.25, 5.29]	-1.72 [-7.35, 3.92]	-3.21 [-8.04, 1.61]	0.16 [-5.26, 5.59]	-3.18 [-8.18, 1.81]	-1.98 [-6.25, 2.29]
Prediabetes	1.54 [-3.38, 6.47]	0.1 [-4.43, 4.63]	1.31 [-2.95, 5.57]	-0.31 [-4.69, 4.07]	-0.92 [-5.71, 3.87]	-0.36 [-4.49, 3.78]	-0.95 [-5.1, 3.2]
Normoglycemia	0.23 [-3.05, 3.52]	0.37 [-2.64, 3.39]	0.22 [-2.63, 3.06]	0.89 [-1.66, 3.43]	-0.06 [-2.93, 2.81]	0.51 [-2.35, 3.37]	0.86 [-1.38, 3.09]
**Diameter left pulmonary artery**							
Diabetes	1.14 [-4.63, 6.91]	0.8 [-3.56, 5.16]	0.41 [-4.29, 5.1]	-0.39 [-4.49, 3.71]	1.94 [-2.5, 6.39]	0.05 [-4.19, 4.28]	0.84 [-2.73, 4.4]
Prediabetes	0.68 [-3.09, 4.46]	0.06 [-3.41, 3.53]	1.12 [-2.15, 4.38]	0.42 [-2.93, 3.77]	-1.11 [-4.78, 2.55]	-0.95 [-4.12, 2.21]	0.51 [-2.68, 3.7]
Normoglycemia	-0.06 [-2.74, 2.63]	0.44 [-2.04, 2.91]	0.96 [-1.36, 3.29]	0.37 [-1.71, 2.46]	-0.62 [-2.97, 1.72]	0.18 [-2.16, 2.53]	0.07 [-1.76, 1.9]

Table S8: Cardiovascular outcomes and long-term exposure to air pollution, stratified by diabetes status – percent change and confidence intervals of the outcome mean per interquartile range increase in the air pollutant of interest. Main model: adjusted for age, sex, height, body mass index, income, marital status, education years and smoking. Significant results with p-value <0.05 are marked with **.

Supplement Table S9 – Air pollution and cardiovascular outcomes, stratified by BMI

Outcome	PM_{10}	PM_{coarse}	$PM_{2.5}$	PNC	NO_2	NO_x	$PM_{25}abs$
	%-change [95%-CI]	*%-change [95%-CI]*	*%-change [95%-CI]*	*%-change [95%-CI]*	*%-change [95%-CI]*	*%-change [95%-CI]*	*%-change [95%-CI]*
**LEFT VENTRICLE**							
**End-diastolic volume**							
BMI <30 [kg/m²]	2.36 [-2.02, 6.74]	2.84 [-1.44, 7.11]	3.48 [-0.66, 7.61]	3.14 [-0.25, 6.52]	2.78 [-1.41, 6.97]	3.23 [-0.49, 6.95]	-0.51 [-5.27, 4.24]
BMI ≥30 [kg/m²]	0.01 [-5.64, 5.66]	-1.76 [-7.61, 4.09]	-0.9 [-6.41, 4.61]	-0.76 [-5.33, 3.81]	0.14 [-5.07, 5.35]	-0.24 [-5.37, 4.88]	-0.15 [-6.56, 6.27]
**End-systolic volume**							
BMI <30 [kg/m²]	1.62 [-6.48, 9.72]	3.05 [-4.85, 10.96]	5.32 [-2.33, 12.97]	4.37 [-1.89, 10.63]	4.57 [-3.17, 12.31]	4.87 [-2.01, 11.76]	1.43 [-7.34, 10.2]
BMI ≥30 [kg/m²]	6.32 [-4.36, 16.99]	0.46 [-10.69, 11.61]	3.3 [-7.15, 13.76]	5.06 [-3.59, 13.71]	3.21 [-6.68, 13.09]	5.11 [-4.59, 14.81]	3.13 [-9.06, 15.32]
**Stroke volume**							
BMI <30 [kg/m²]	2.63 [-1.58, 6.85]	2.71 [-1.4, 6.83]	2.6 [-1.4, 6.59]	2.55 [-0.71, 5.82]	1.95 [-2.09, 6]	2.46 [-1.14, 6.05]	-1.46 [-6.03, 3.12]
BMI ≥30 [kg/m²]	-2.92 [-8.16, 2.32]	-2.78 [-8.22, 2.67]	-2.83 [-7.95, 2.28]	-3.45 [-7.67, 0.77]	-1.27 [-6.12, 3.58]	-2.71 [-7.47, 2.04]	-1.66 [-7.64, 4.32]
**Ejection fraction**							
BMI <30 [kg/m²]	0.61 [-1.79, 3.01]	0.35 [-2, 2.69]	-0.52 [-2.79, 1.76]	-0.12 [-1.98, 1.75]	-0.47 [-2.77, 1.83]	-0.36 [-2.41, 1.68]	-0.5 [-3.1, 2.1]
BMI ≥30 [kg/m²]	-1.97 [-4.73, 0.78]	-0.41 [-3.3, 2.48]	-1.36 [-4.07, 1.34]	-1.8 [-4.03, 0.43]	-0.84 [-3.4, 1.73]	-1.79 [-4.29, 0.72]	-1.08 [-4.23, 2.08]
**Diastolic myocardial mass**							
BMI <30 [kg/m²]	1.07 [-2.13, 4.26]	1.49 [-1.63, 4.6]	0.61 [-2.42, 3.63]	-0.08 [-2.56, 2.4]	-0.75 [-3.81, 2.31]	-0.4 [-3.13, 2.32]	-1.8 [-5.25, 1.65]
BMI ≥30 [kg/m²]	-1.16 [-6.05, 3.74]	-2.16 [-7.23, 2.9]	-1.58 [-6.35, 3.19]	-1.14 [-5.11, 2.82]	-2.55 [-7.05, 1.94]	-1.76 [-6.19, 2.67]	-4.11 [-9.62, 1.41]
**Left ventricular remodeling**							
BMI <30 [kg/m²]	-0.11 [-0.66, 0.44]	-0.02 [-0.56, 0.52]	-0.17 [-0.69, 0.35]	-0.32 [-0.75, 0.1]	-0.31 [-0.84, 0.21]	-0.33 [-0.79, 0.14]	-0.1 [-0.69, 0.49]
BMI ≥30 [kg/m²]	-0.22 [-1, 0.57]	-0.12 [-0.93, 0.7]	-0.11 [-0.88, 0.65]	-0.10 [-0.74, 0.53]	-0.53 [-1.25, 0.18]	-0.3 [-1.01, 0.41]	-0.67 [-1.55, 0.21]
**Global segments**							
BMI <30 [kg/m²]	-0.48 [-2.85, 1.89]	0 [-2.31, 2.31]	-0.77 [-3.02, 1.47]	-1.66 [-3.49, 0.16]	-1.9 [-4.16, 0.35]	-2.02 [-4.02, -0.02]**	-0.83 [-3.39, 1.73]
BMI ≥30 [kg/m²]	-1.17 [-4.55, 2.2]	-0.94 [-4.44, 2.57]	-1.31 [-4.59, 1.98]	-0.7 [-3.43, 2.04]	-2.42 [-5.51, 0.66]	-1.55 [-4.6, 1.51]	-3.53 [-7.32, 0.25]
**Basal segments**							
BMI <30 [kg/m²]	-1.81 [-4.51, 0.89]	-1.26 [-3.91, 1.38]	-1 [-3.57, 1.56]	-2.61 [-4.69, -0.54]**	-2.64 [-5.21, -0.07]**	-2.98 [-5.26, -0.71]**	-1.14 [-4.07, 1.79]
BMI ≥30 [kg/m²]	-1.09 [-4.84, 2.67]	-0.49 [-4.39, 3.41]	-2.31 [-5.95, 1.33]	-0.8 [-3.84, 2.24]	-2.84 [-6.26, 0.59]	-2.02 [-5.41, 1.37]	-3.97 [-8.18, 0.24]
**Mid segments**							

BMI <30 [kg/m²]	0.01 [-2.79, 2.8]	0.76 [-1.97, 3.48]	-0.79 [-3.44, 1.85]	-1.54 [-3.69, 0.62]	-1.72 [-4.39, 0.94]	-1.96 [-4.33, 0.41]	-0.97 [-3.99, 2.05]
BMI ≥30 [kg/m²]	-1.31 [-5.31, 2.69]	-1.84 [-5.99, 2.3]	-1.13 [-5.03, 2.77]	-0.74 [-3.98, 2.51]	-3.11 [-6.76, 0.54]	-1.58 [-5.21, 2.05]	-4.47 [-8.95, 0.01]
**Apical segments**							
BMI <30 [kg/m²]	1.06 [-2.16, 4.28]	0.96 [-2.18, 4.1]	-0.35 [-3.4, 2.7]	-0.22 [-2.72, 2.28]	-0.95 [-4.03, 2.13]	-0.45 [-3.2, 2.3]	-0.06 [-3.55, 3.42]
BMI ≥30 [kg/m²]	-1.12 [-5.29, 3.05]	-0.2 [-4.53, 4.13]	0.15 [-3.92, 4.22]	-0.45 [-3.83, 2.93]	-0.57 [-4.41, 3.27]	-0.68 [-4.46, 3.11]	-1.22 [-5.96, 3.51]
**Lateral segments**							
BMI <30 [kg/m²]	-0.39 [-2.87, 2.1]	-0.26 [-2.69, 2.17]	-0.74 [-3.1, 1.61]	-1.43 [-3.35, 0.49]	-1.67 [-4.04, 0.7]	-1.75 [-3.86, 0.36]	-0.88 [-3.57, 1.81]
BMI ≥30 [kg/m²]	-1.85 [-5.54, 1.84]	-1.57 [-5.41, 2.26]	-0.81 [-4.42, 2.81]	-0.8 [-3.8, 2.2]	-2.85 [-6.23, 0.52]	-1.65 [-5, 1.7]	-4.16 [-8.3, -0.02]**
**Inferior segments**							
BMI <30 [kg/m²]	-1.44 [-4.07, 1.2]	-0.26 [-2.85, 2.32]	-0.56 [-3.07, 1.94]	-2.01 [-4.05, 0.02]	-2.51 [-5.02, 0.00]	-2.46 [-4.69, -0.23]**	-2.4 [-5.25, 0.44]
BMI ≥30 [kg/m²]	-0.93 [-4.39, 2.52]	-0.52 [-4.11, 3.07]	-1.54 [-4.9, 1.82]	-0.08 [-2.88, 2.72]	-2.81 [-5.96, 0.34]	-1.04 [-4.18, 2.09]	-3.67 [-7.55, 0.2]
**Anterior segments**							
BMI <30 [kg/m²]	0.46 [-2.74, 3.66]	0.1 [-3.02, 3.23]	-0.57 [-3.6, 2.46]	-1.45 [-3.93, 1.03]	-1.94 [-4.99, 1.11]	-1.98 [-4.7, 0.74]	0.09 [-3.37, 3.55]
BMI ≥30 [kg/m²]	-0.63 [-5.02, 3.76]	-0.41 [-4.97, 4.15]	-0.22 [-4.5, 4.06]	-0.41 [-3.97, 3.15]	-1.97 [-6, 2.07]	-1.15 [-5.13, 2.83]	-2.83 [-7.79, 2.13]
**Septal segments**							
BMI <30 [kg/m²]	-1.03 [-3.74, 1.69]	0.24 [-2.41, 2.89]	-1.1 [-3.67, 1.47]	-2.19 [-4.28, -0.1]**	-2.17 [-4.75, 0.42]	-2.47 [-4.76, -0.17]**	-0.55 [-3.49, 2.39]
BMI ≥30 [kg/m²]	-1.09 [-4.57, 2.4]	-1.22 [-4.84, 2.39]	-2.94 [-6.31, 0.42]	-1.48 [-4.3, 1.33]	-2.71 [-5.89, 0.47]	-2.5 [-5.63, 0.64]	-3.9 [-7.8, 0.00]
**RIGHT VENTRICLE**							
**End-diastolic volume**							
BMI <30 [kg/m²]	0.78 [-3.35, 4.91]	0.35 [-3.75, 4.44]	2.77 [-1.11, 6.64]	1.2 [-2.15, 4.55]	1.4 [-2.6, 5.41]	1.75 [-1.87, 5.36]	-3.08 [-7.61, 1.44]
BMI ≥30 [kg/m²]	-3.34 [-8.34, 1.66]	-4.6 [-10.2, 1.01]	-3.19 [-8.38, 2]	-2.9 [-7.05, 1.24]	-2.57 [-7.47, 2.32]	-2.83 [-7.69, 2.03]	-3.52 [-9.53, 2.48]
**End-systolic volume**							
BMI <30 [kg/m²]	-0.05 [-5.94, 5.85]	-0.72 [-6.56, 5.13]	2.16 [-3.39, 7.71]	0.79 [-4, 5.59]	0.8 [-4.92, 6.52]	1.54 [-3.63, 6.71]	-4.38 [-10.84, 2.08]
BMI ≥30 [kg/m²]	-3.18 [-9.78, 3.41]	-4.92 [-12.31, 2.48]	-3.98 [-10.8, 2.84]	-2.12 [-7.6, 3.37]	-3.96 [-10.38, 2.46]	-2.81 [-9.22, 3.59]	-3.79 [-11.7, 4.11]
**Stroke volume**							
BMI <30 [kg/m²]	1.47 [-2.62, 5.55]	1.25 [-2.8, 5.3]	3.27 [-0.56, 7.1]	1.52 [-1.8, 4.84]	1.93 [-2.03, 5.9]	1.92 [-1.67, 5.5]	-1.94 [-6.44, 2.55]
BMI ≥30 [kg/m²]	-3.47 [-8.9, 1.96]	-4.34 [-10.45, 1.76]	-2.45 [-8.1, 3.21]	-3.65 [-8.14, 0.84]	-1.28 [-6.62, 4.06]	-2.89 [-8.18, 2.39]	-3.27 [-9.8, 3.26]
**Ejection fraction**							
BMI <30 [kg/m²]	0.91 [-1.76, 3.57]	0.94 [-1.69, 3.58]	0.71 [-1.8, 3.22]	0.42 [-1.75, 2.58]	0.57 [-2.01, 3.16]	0.14 [-2.2, 2.48]	1.19 [-1.73, 4.12]
BMI ≥30 [kg/m²]	0.19 [-2.94, 3.32]	0.84 [-2.69, 4.36]	1.01 [-2.24, 4.25]	-0.59 [-3.19, 2.01]	1.69 [-1.34, 4.73]	0.14 [-2.9, 3.18]	0.67 [-3.09, 4.42]

VESSLES							
**Diameter ascending Aorta**							
BMI <30 [kg/m²]	2.25 [0.09, 4.4]	1.22 [-0.96, 3.4]	1.76 [-0.33, 3.85]	2.25 [0.58, 3.91]	2 [-0.14, 4.15]	2.36 [0.5, 4.22]	1.76 [-0.66, 4.18]
BMI ≥30 [kg/m²]	-1.27 [-3.94, 1.41]	-2.02 [-4.89, 0.84]	-1.99 [-4.48, 0.51]	-0.94 [-3.18, 1.29]	-1.09 [-3.62, 1.43]	-1.1 [-3.56, 1.36]	-0.58 [-3.63, 2.48]
**Maximum diameter infrarenal**							
BMI <30 [kg/m²]	-0.58 [-2.6, 1.45]	-1.16 [-3.19, 0.87]	0.56 [-1.39, 2.52]	-0.67 [-2.24, 0.91]	-1.05 [-3.06, 0.96]	-0.31 [-2.07, 1.44]	-1.72 [-3.97, 0.53]
BMI ≥30 [kg/m²]	-0.14 [-3.34, 3.06]	-1.2 [-4.64, 2.23]	-1.94 [-4.92, 1.05]	-0.11 [-2.8, 2.57]	-0.43 [-3.45, 2.59]	-0.94 [-3.88, 2.01]	-0.91 [-4.55, 2.73]
**Diameter pulmonary trunk**							
BMI <30 [kg/m²]	2.06 [-0.29, 4.41]	2.02 [-0.35, 4.38]	0.28 [-2.01, 2.57]	2.13 [0.31, 3.96]**	1.3 [-1.04, 3.65]	1.81 [-0.22, 3.85]	0.3 [-2.35, 2.94]
BMI ≥30 [kg/m²]	-2.22 [-5.37, 0.93]	-3.93 [-7.27, -0.58]**	-2.75 [-5.69, 0.19]	-1.93 [-4.56, 0.71]	-1.9 [-4.88, 1.08]	-2.17 [-5.06, 0.73]	-1.42 [-5.02, 2.19]
**Diameter right pulmonary artery**							
BMI <30 [kg/m²]	0.98 [-2.00, 3.96]	0.56 [-2.44, 3.57]	0.2 [-2.68, 3.09]	0.78 [-1.55, 3.10]	-0.04 [-3.01, 2.93]	0.39 [-2.20, 2.98]	-0.34 [-3.67, 3.00]
BMI ≥30 [kg/m²]	-0.85 [-4.14, 2.44]	0.08 [-3.47, 3.63]	-0.33 [-3.43, 2.76]	-0.3 [-3.06, 2.46]	1.12 [-1.98, 4.23]	0.23 [-2.81, 3.26]	1.41 [-2.34, 5.15]
**Diameter left pulmonary artery**							
BMI <30 [kg/m²]	-0.8 [-3.17, 1.56]	0.0 [-2.38, 2.39]	-2.0 [-4.27, 0.28]	-0.34 [-2.19, 1.51]	-0.56 [-2.91, 1.8]	-1.06 [-3.11, 0.99]	-1.75 [-4.38, 0.89]
BMI ≥30 [kg/m²]	-0.51 [-3.38, 2.35]	-0.54 [-3.63, 2.54]	-0.65 [-3.34, 2.04]	0.01 [-2.39, 2.42]	0.58 [-2.12, 3.28]	0.37 [-2.27, 3.01]	0.28 [-2.98, 3.55]

Table S9: Cardiovascular outcomes and long-term exposure to air pollution, stratified by BMI – percent change and confidence intervals of the outcome mean per interquartile range increase in the air pollutant of interest. Main model: adjusted for age, sex, height, income, marital status, education years and smoking. Significant results with p-value <0.05 are marked with **.

Supplement Table S10 – Air pollution and cardiovascular outcomes, stratified by hs-CRP

Outcome	PM_{10}	PM_{coarse}	$PM_{2.5}$	PNC	NO_2	NO_x	$PM_{25}abs$
	%-change [95%-CI]	*%-change [95%-CI]*	*%-change [95%-CI]*	*%-change [95%-CI]*	*%-change [95%-CI]*	*%-change [95%-CI]*	*%-change [95%-CI]*
**LEFT VENTRICLE**							
**End-diastolic volume**							
hsCRP < 1 mg/dl	0.52 [-5.15, 6.20]	4.40 [-0.56, 9.35]	3.73 [-1.30, 8.75]	5.26 [0.78, 9.73]**	3.34 [-1.85, 8.53]	4.89 [-0.16, 9.94]	4.76 [0.74, 8.79]
hsCRP ≥ 1 mg/dl	-0.44 [-5.99, 5.11]	-0.90 [-6.04, 4.24]	0.91 [-3.73, 5.55]	0.36 [-4.07, 4.78]	-0.17 [-5.12, 4.78]	0.34 [-4.38, 5.07]	-0.22 [-4.20, 3.76]
**End-systolic volume**							
hsCRP < 1 mg/dl	2.57 [-8.29, 13.44]	3.70 [-5.88, 13.27]	8.41 [-1.19, 18.00]	10.46 [1.90, 19.02]**	5.50 [-4.45, 15.45]	6.67 [-3.07, 16.40]	7.82 [0.07, 15.57]**
hsCRP ≥ 1 mg/dl	0.65 [-9.78, 11.08]	0.04 [-9.63, 9.71]	1.24 [-7.48, 9.96]	0.98 [-7.35, 9.31]	0.02 [-9.28, 9.33]	1.95 [-6.93, 10.83]	1.63 [-5.84, 9.11]
**Stroke volume**							
hsCRP < 1 mg/dl	-0.49 [-6.19, 5.20]	4.67 [-0.29, 9.63]	1.54 [-3.52, 6.61]	2.83 [-1.71, 7.38]	2.34 [-2.88, 7.55]	3.94 [-1.15, 9.03]	3.34 [-0.73, 7.41]
hsCRP ≥ 1 mg/dl	-0.96 [-6.02, 4.10]	-1.34 [-6.03, 3.36]	0.76 [-3.47, 5.00]	0.06 [-3.99, 4.10]	-0.34 [-4.86, 4.17]	-0.37 [-4.69, 3.94]	-1.09 [-4.72, 2.54]
**Ejection fraction**							
hsCRP < 1 mg/dl	-0.61 [-4.03, 2.81]	0.59 [-2.43, 3.61]	-1.65 [-4.69, 1.39]	-1.84 [-4.57, 0.89]	-0.90 [-4.04, 2.24]	-0.40 [-3.49, 2.68]	-0.97 [-3.44, 1.49]
hsCRP ≥ 1 mg/dl	-0.10 [-2.80, 2.60]	0.27 [-2.24, 2.77]	0.33 [-1.93, 2.59]	0.37 [-1.79, 2.52]	0.73 [-1.67, 3.14]	-0.24 [-2.55, 2.06]	0.03 [-1.90, 1.97]
**Diastolic myocardial mass**							
hsCRP < 1 mg/dl	-1.66 [-5.52, 2.19]	0.28 [-3.14, 3.69]	-1.50 [-4.94, 1.94]	-0.46 [-3.57, 2.64]	0.68 [-2.87, 4.23]	-0.79 [-4.27, 2.69]	0.91 [-1.88, 3.70]
hsCRP ≥ 1 mg/dl	-3.78 [-8.15, 0.59]	-0.04 [-4.13, 4.04]	-1.09 [-4.77, 2.59]	0.12 [-3.40, 3.63]	0.15 [-3.78, 4.08]	0.99 [-2.76, 4.74]	-0.65 [-3.80, 2.51]
**Left ventricular remodeling**							
hsCRP < 1 mg/dl	-2.34 [-7.71, 3.04]	-3.85 [-8.56, 0.87]	-4.95 [-9.69, -0.22]**	-5.19 [-9.44, -0.95]**	-2.88 [-7.81, 2.05]	-5.13 [-9.92, -0.35]**	-3.43 [-7.28, 0.43]
hsCRP ≥ 1 mg/dl	-4.03 [-9.93, 1.86]	1.04 [-4.45, 6.54]	-2.66 [-7.61, 2.28]	-0.20 [-4.93, 4.53]	0.39 [-4.90, 5.68]	1.27 [-3.78, 6.32]	-0.24 [-4.49, 4.01]
**Global segments**							
hsCRP < 1 mg/dl	-1.19 [-4.19, 1.80]	-1.31 [-3.95, 1.33]	-2.31 [-4.95, 0.34]	-2.40 [-4.77, -0.02]**	-0.67 [-3.42, 2.08]	-2.21 [-4.89, 0.47]	-1.18 [-3.34, 0.98]
hsCRP ≥ 1 mg/dl	-3.06 [-5.96, -0.17]**	0.06 [-2.66, 2.77]	-1.99 [-4.42, 0.45]	-0.84 [-3.18, 1.49]	-0.72 [-3.33, 1.89]	-0.01 [-2.51, 2.49]	-1.01 [-3.11, 1.08]
**Basal segments**							
hsCRP < 1 mg/dl	-2.24 [-5.74, 1.26]	-2.53 [-5.61, 0.55]	-3.10 [-6.19, -0.00]**	-3.65 [-6.41, -0.89]**	-2.22 [-5.43, 1.00]	-2.83 [-5.97, 0.31]	-2.38 [-4.89, 0.13]

hsCRP ≥ 1 mg/dl	-3.31 [-6.63, 0.01]	-0.82 [-3.93, 2.29]	-3.16 [-5.93, -0.39]**	-1.79 [-4.46, 0.88]	-1.54 [-4.53, 1.44]	-1.00 [-3.86, 1.86]	-1.61 [-4.01, 0.78]
**Mid segments**							
hsCRP < 1 mg/dl	-1.29 [-4.80, 2.22]	-1.19 [-4.28, 1.91]	-2.71 [-5.81, 0.40]	-2.47 [-5.26, 0.33]	-0.33 [-3.56, 2.90]	-2.78 [-5.91, 0.36]	-1.10 [-3.64, 1.43]
hsCRP ≥ 1 mg/dl	-3.44 [-6.87, 0.00]	0.55 [-2.67, 3.78]	-1.54 [-4.44, 1.36]	-0.22 [-3.00, 2.55]	-0.12 [-3.22, 2.98]	1.02 [-1.94, 3.98]	-0.59 [-3.08, 1.91]
**Apical segments**							
hsCRP < 1 mg/dl	0.82 [-3.23, 4.87]	0.63 [-2.95, 4.21]	-0.26 [-3.88, 3.35]	-0.10 [-3.35, 3.16]	1.50 [-2.22, 5.21]	-0.19 [-3.84, 3.46]	0.81 [-2.12, 3.73]
hsCRP ≥ 1 mg/dl	-2.05 [-5.93, 1.83]	0.78 [-2.83, 4.38]	-0.70 [-3.95, 2.56]	-0.23 [-3.34, 2.87]	-0.31 [-3.78, 3.17]	-0.00 [-3.32, 3.31]	-0.70 [-3.49, 2.08]
**Lateral segments**							
hsCRP < 1 mg/dl	-1.28 [-4.33, 1.77]	-1.73 [-4.41, 0.96]	-2.20 [-4.90, 0.51]	-1.97 [-4.40, 0.47]	-0.84 [-3.65, 1.97]	-1.88 [-4.62, 0.86]	-0.85 [-3.05, 1.36]
hsCRP ≥ 1 mg/dl	-3.46 [-6.62, -0.30]**	-0.53 [-3.50, 2.44]	-2.04 [-4.70, 0.63]	-0.70 [-3.25, 1.86]	-0.86 [-3.71, 1.99]	0.44 [-2.28, 3.17]	-0.94 [-3.23, 1.35]
**Inferior segments**							
hsCRP < 1 mg/dl	-2.55 [-5.87, 0.76]	-1.29 [-4.23, 1.65]	-3.23 [-6.16, -0.30]**	-2.45 [-5.10, 0.20]	-1.22 [-4.28, 1.84]	-2.14 [-5.13, 0.84]	-1.03 [-3.44, 1.38]
hsCRP ≥ 1 mg/dl	-4.18 [-7.39, -0.97]**	-0.12 [-3.15, 2.91]	-2.54 [-5.25, 0.17]	-1.23 [-3.83, 1.37]	-1.57 [-4.47, 1.34]	-0.19 [-2.98, 2.59]	-1.29 [-3.63, 1.05]
**Anterior segments**							
hsCRP < 1 mg/dl	-0.75 [-4.93, 3.43]	-1.78 [-5.46, 1.89]	-2.73 [-6.43, 0.97]	-3.00 [-6.32, 0.32]	-0.24 [-4.08, 3.60]	-1.80 [-5.55, 1.96]	-1.52 [-4.53, 1.49]
hsCRP ≥ 1 mg/dl	-1.52 [-5.39, 2.36]	1.15 [-2.44, 4.74]	-1.17 [-4.41, 2.07]	-0.01 [-3.10, 3.09]	0.73 [-2.72, 4.19]	0.92 [-2.38, 4.23]	-0.18 [-2.96, 2.60]
**Septal segments**							
hsCRP < 1 mg/dl	-1.04 [-4.53, 2.45]	-0.84 [-3.92, 2.25]	-2.19 [-5.29, 0.90]	-3.08 [-5.84, -0.32]	-0.97 [-4.18, 2.24]	-3.08 [-6.19, 0.03]	-1.93 [-4.43, 0.58]
hsCRP ≥ 1 mg/dl	-3.30 [-6.45, -0.16]**	-0.24 [-3.19, 2.71]	-2.72 [-5.36, -0.09]**	-1.75 [-4.27, 0.78]	-1.19 [-4.03, 1.64]	-1.40 [-4.11, 1.30]	-1.80 [-4.06, 0.47]
**RIGHT VENTRICLE**							
**End-diastolic volume**							
hsCRP < 1 mg/dl	-2.28 [-8.13, 3.56]	2.75 [-2.52, 8.02]	1.44 [-3.93, 6.80]	2.80 [-2.14, 7.74]	0.68 [-4.72, 6.07]	3.67 [-1.58, 8.92]	1.89 [-2.57, 6.35]
hsCRP ≥ 1 mg/dl	-2.34 [-7.15, 2.47]	-3.10 [-7.52, 1.32]	-0.29 [-4.24, 3.66]	-0.71 [-4.49, 3.08]	-1.70 [-5.73, 2.33]	0.08 [-3.97, 4.12]	-1.30 [-4.69, 2.08]
**End-systolic volume**							
hsCRP < 1 mg/dl	-2.85 [-11.37, 5.67]	3.23 [-4.45, 10.91]	1.80 [-6.01, 9.61]	3.56 [-3.64, 10.75]	1.14 [-6.71, 9.00]	2.23 [-5.46, 9.93]	2.41 [-4.09, 8.91]
hsCRP ≥ 1 mg/dl	-2.93 [-9.35, 3.49]	-4.85 [-10.73, 1.04]	-1.72 [-6.98, 3.55]	-1.16 [-6.21, 3.89]	-2.85 [-8.22, 2.51]	0.23 [-5.16, 5.63]	-1.30 [-5.82, 3.21]
**Stroke volume**							
hsCRP < 1 mg/dl	-1.89 [-7.55, 3.76]	2.23 [-2.87, 7.32]	1.03 [-4.16, 6.22]	2.01 [-2.78, 6.79]	0.10 [-5.11, 5.32]	4.94 [-0.10, 9.98]	1.29 [-3.03, 5.61]

hsCRP ≥ 1 mg/dl	-1.76 [-6.99, 3.47]	-1.58 [-6.40, 3.23]	1.05 [-3.24, 5.33]	-0.31 [-4.42, 3.81]	-0.65 [-5.02, 3.73]	-0.08 [-4.47, 4.31]	-1.32 [-4.99, 2.35]
**Ejection fraction**							
hsCRP < 1 mg/dl	0.62 [-3.05, 4.29]	-0.38 [-3.69, 2.93]	-0.15 [-3.51, 3.22]	-0.74 [-3.85, 2.37]	-0.80 [-4.17, 2.58]	1.59 [-1.71, 4.90]	-0.59 [-3.39, 2.21]
hsCRP ≥ 1 mg/dl	0.68 [-2.50, 3.86]	1.86 [-1.06, 4.78]	1.45 [-1.15, 4.05]	0.58 [-1.92, 3.08]	1.43 [-1.22, 4.09]	0.11 [-2.56, 2.78]	0.21 [-2.03, 2.45]
**VESSLES**							
**Diameter ascending Aorta**							
hsCRP < 1 mg/dl	-0.71 [-3.98, 2.56]	-0.77 [-3.63, 2.08]	-0.96 [-3.92, 1.99]	-0.58 [-3.17, 2.01]	0.06 [-2.83, 2.96]	-1.00 [-3.90, 1.91]	0.06 [-2.23, 2.35]
hsCRP ≥ 1 mg/dl	1.86 [-0.53, 4.25]	1.09 [-1.18, 3.36]	1.64 [-0.39, 3.66]	2.56 [0.68, 4.43]	1.40 [-0.72, 3.51]	1.24 [-0.77, 3.25]	2.19 [0.49, 3.89]
**Maximum diameter infrarenal**							
hsCRP < 1 mg/dl	-4.30 [-7.38, -1.21]	-2.70 [-5.43, 0.03]	-3.43 [-6.23, -0.62]	-2.09 [-4.57, 0.40]	-2.75 [-5.52, 0.01]	-2.23 [-5.02, 0.56]	-1.80 [-4.00, 0.40]
hsCRP ≥ 1 mg/dl	-0.07 [-2.64, 2.50]	-0.49 [-2.93, 1.94]	0.38 [-1.80, 2.56]	0.18 [-1.87, 2.23]	0.35 [-1.93, 2.62]	-0.33 [-2.49, 1.83]	0.20 [-1.65, 2.05]
**Diameter pulmonary trunk**							
hsCRP < 1 mg/dl	0.02 [-3.56, 3.60]	2.06 [-1.04, 5.17]	0.76 [-2.47, 3.99]	1.48 [-1.34, 4.30]	1.56 [-1.59, 4.72]	0.17 [-3.01, 3.36]	2.01 [-0.47, 4.49]
hsCRP ≥ 1 mg/dl	-0.54 [-3.43, 2.34]	-1.88 [-4.60, 0.84]	-0.84 [-3.29, 1.60]	-0.70 [-3.00, 1.60]	-0.73 [-3.28, 1.83]	-2.18 [-4.58, 0.22]	-0.32 [-2.40, 1.75]
**Diameter right pulmonary artery**							
hsCRP < 1 mg/dl	0.14 [-4.00, 4.27]	2.08 [-1.51, 5.67]	0.03 [-3.70, 3.76]	0.57 [-2.70, 3.83]	1.69 [-1.95, 5.34]	-0.44 [-4.11, 3.24]	0.97 [-1.91, 3.86]
hsCRP ≥ 1 mg/dl	0.29 [-2.98, 3.55]	-1.35 [-4.43, 1.74]	0.42 [-2.35, 3.19]	-0.11 [-2.71, 2.49]	-0.40 [-3.29, 2.49]	-0.21 [-2.95, 2.53]	-0.11 [-2.46, 2.23]
**Diameter left pulmonary artery**							
hsCRP < 1 mg/dl	-3.27 [-6.66, 0.13]	-0.79 [-3.79, 2.20]	-1.91 [-4.99, 1.18]	-1.23 [-3.94, 1.48]	-2.06 [-5.09, 0.96]	-1.76 [-4.80, 1.28]	-0.74 [-3.15, 1.66]
hsCRP ≥ 1 mg/dl	1.50 [-1.16, 4.16]	0.93 [-1.58, 3.45]	1.69 [-0.56, 3.93]	1.18 [-0.93, 3.30]	0.72 [-1.64, 3.08]	0.06 [-2.17, 2.30]	1.02 [-0.89, 2.93]

Table S10: Cardiovascular outcomes and long-term exposure to air pollution, stratified by hs-CRP – percent change and confidence intervals of the outcome mean per interquartile range increase in the air pollutant of interest. Main model: adjusted for age, sex, height, body mass index, income, marital status, education years and smoking. Significant results with p-value <0.05 are marked with **.

Supplement Table S11 – Air pollution and cardiovascular outcomes, stratified by hypertension

Outcome	PM_{10}	PM_{coarse}	$PM_{2.5}$	PNC	NO_2	NO_x	$PM_{25}abs$
	%-change [95%-CI]	*%-change [95%-CI]*	*%-change [95%-CI]*	*%-change [95%-CI]*	*%-change [95%-CI]*	*%-change [95%-CI]*	*%-change [95%-CI]*
**LEFT VENTRICLE**							
**End-diastolic volume**							
Hypertension = no	2.11 [-2.73, 6.95]	1.73 [-2.54, 6.01]	2.55 [-1.64, 6.74]	2.11 [-1.76, 5.99]	1.2 [-3.1, 5.5]	3.76 [-0.46, 7.98]	1.61 [-1.9, 5.11]
Hypertension = yes	-2.24 [-9.08, 4.6]	-1.28 [-7.29, 4.73]	0.6 [-4.89, 6.09]	0.07 [-4.99, 5.13]	0.81 [-5.29, 6.91]	-0.47 [-5.91, 4.97]	-0.29 [-4.86, 4.29]
**End-systolic volume**							
Hypertension = no	3.53 [-5.32, 12.38]	0.98 [-6.84, 8.8]	3.59 [-4.09, 11.26]	2.78 [-4.31, 9.87]	1.2 [-6.66, 9.05]	5.54 [-2.18, 13.27]	2.34 [-4.07, 8.75]
Hypertension = yes	-2.2 [-15.6, 11.19]	-1.34 [-13.1, 10.42]	1.83 [-8.9, 12.56]	3.04 [-6.84, 12.91]	2.36 [-9.56, 14.28]	0.23 [-10.4, 10.87]	2.46 [-6.48, 11.4]
**Stroke volume**							
Hypertension = no	1.55 [-2.96, 6.05]	2.14 [-1.83, 6.11]	2.17 [-1.73, 6.07]	1.85 [-1.76, 5.45]	1.24 [-2.76, 5.23]	3 [-0.93, 6.93]	1.29 [-1.97, 4.56]
Hypertension = yes	-2.47 [-9.07, 4.12]	-1.39 [-7.19, 4.41]	-0.11 [-5.4, 5.19]	-1.4 [-6.27, 3.48]	-0.08 [-5.96, 5.81]	-0.91 [-6.15, 4.34]	-1.63 [-6.04, 2.77]
**Ejection fraction**							
Hypertension = no	-0.48 [-3.08, 2.12]	0.66 [-1.63, 2.95]	-0.31 [-2.56, 1.95]	-0.17 [-2.25, 1.91]	0.06 [-2.24, 2.37]	-0.65 [-2.93, 1.62]	-0.14 [-2.02, 1.74]
Hypertension = yes	0.97 [-2.65, 4.59]	0.71 [-2.46, 3.89]	0.41 [-2.49, 3.31]	-0.29 [-2.96, 2.39]	0.45 [-2.77, 3.68]	0.58 [-2.29, 3.45]	-0.1 [-2.52, 2.32]
**Diastolic myocardial mass**							
Hypertension = no	-1.97 [-5.35, 1.41]	0.47 [-2.52, 3.46]	-0.42 [-3.36, 2.52]	0.07 [-2.64, 2.79]	0.33 [-2.68, 3.34]	0.09 [-2.88, 3.06]	0.09 [-2.37, 2.54]
Hypertension = yes	-4.04 [-9.58, 1.51]	-0.69 [-5.59, 4.22]	-2.24 [-6.7, 2.22]	-1.93 [-6.04, 2.18]	-0.11 [-5.08, 4.87]	-0.29 [-4.73, 4.15]	-1.45 [-5.17, 2.28]
**Left ventricular remodeling**							
Hypertension = no	-0.13 [-0.63, 0.37]	-0.13 [-0.63, 0.37]	-0.37 [-0.86, 0.12]	-0.19 [-0.59, 0.22]	-0.38 [-0.87, 0.1]	-0.23 [-0.68, 0.22]	-0.55 [-1.11, 0.01]
Hypertension = yes	-0.01 [-0.85, 0.82]	0.16 [-0.67, 0.98]	0.16 [-0.59, 0.9]	-0.04 [-0.67, 0.59]	-0.32 [-1.07, 0.43]	-0.17 [-0.86, 0.52]	-0.15 [-1.09, 0.79]
**Global segments**							
Hypertension = no	-2.77 [-5.35, -0.19]**	-0.47 [-2.77, 1.83]	-1.89 [-4.13, 0.36]	-1.17 [-3.25, 0.92]	-0.8 [-3.11, 1.51]	-1.5 [-3.77, 0.78]	-0.99 [-2.87, 0.89]
Hypertension = yes	-1.57 [-5.26, 2.11]	0.35 [-2.9, 3.59]	-1.77 [-4.72, 1.17]	-1.67 [-4.39, 1.04]	-0.16 [-3.45, 3.13]	-0.25 [-3.19, 2.68]	-0.9 [-3.37, 1.56]
**Basal segments**							
Hypertension = no	-2.44 [-5.38, 0.51]	-0.42 [-3.03, 2.2]	-1.6 [-4.16, 0.97]	-0.96 [-3.33, 1.42]	-0.79 [-3.41, 1.84]	-0.99 [-3.59, 1.6]	-0.95 [-3.09, 1.19]
Hypertension = yes	-3.02 [-6.92, 0.89]	-1.33 [-4.77, 2.12]	-3.47 [-6.57, -0.37]**	-3.42 [-6.26, -0.57]**	-2.26 [-5.74, 1.22]	-1.71 [-4.82, 1.4]	-2.2 [-4.8, 0.4]
**Mid segments**							

Hypertension = no	-3.2 [-6.24, -0.16]**	-0.28 [-3, 2.43]	-2.11 [-4.76, 0.55]	-1.22 [-3.68, 1.24]	-0.5 [-3.23, 2.22]	-2.05 [-4.73, 0.62]	-0.81 [-3.04, 1.41]
Hypertension = yes	-2.16 [-6.67, 2.36]	0.24 [-3.74, 4.21]	-1.96 [-5.57, 1.66]	-1.79 [-5.12, 1.54]	-0.04 [-4.07, 3.99]	0.3 [-3.3, 3.89]	-1.17 [-4.19, 1.85]
**Apical segments**							
Hypertension = no	-2.7 [-6.25, 0.85]	-0.9 [-4.04, 2.24]	-2.07 [-5.15, 1.01]	-1.48 [-4.33, 1.37]	-1.35 [-4.51, 1.81]	-1.49 [-4.61, 1.63]	-1.39 [-3.97, 1.18]
Hypertension = yes	1.96 [-2.94, 6.86]	3.53 [-0.74, 7.8]	1.53 [-2.39, 5.46]	1.61 [-2, 5.23]	3.39 [-0.94, 7.72]	1.4 [-2.49, 5.29]	1.86 [-1.4, 5.12]
**Lateral segments**							
Hypertension = no	-2.71 [-5.57, 0.16]	-0.51 [-3.06, 2.03]	-1.66 [-4.15, 0.84]	-0.74 [-3.05, 1.57]	-0.68 [-3.24, 1.88]	-0.98 [-3.5, 1.55]	-0.64 [-2.73, 1.45]
Hypertension = yes	-2.66 [-6.41, 1.08]	-1.1 [-4.4, 2.21]	-2.51 [-5.5, 0.48]	-2.33 [-5.08, 0.43]	-1.41 [-4.75, 1.94]	-0.82 [-3.81, 2.17]	-1.49 [-3.99, 1.02]
**Inferior segments**							
Hypertension = no	-3.12 [-6.02, -0.21]**	0.08 [-2.51, 2.67]	-1.57 [-4.11, 0.97]	-0.9 [-3.25, 1.45]	-0.73 [-3.33, 1.88]	-1.06 [-3.63, 1.51]	-0.64 [-2.76, 1.48]
Hypertension = yes	-3.57 [-7.23, 0.08]	-0.66 [-3.92, 2.59]	-3.18 [-6.1, -0.26]**	-2.17 [-4.88, 0.54]	-1.45 [-4.75, 1.84]	-0.61 [-3.55, 2.34]	-1.31 [-3.78, 1.15]
**Anterior segments**							
Hypertension = no	-3.45 [-6.87, -0.03]**	-1.2 [-4.24, 1.84]	-2.96 [-5.92, 0.01]	-2.06 [-4.81, 0.69]	-1.18 [-4.24, 1.87]	-2.26 [-5.27, 0.74]	-1.7 [-4.18, 0.79]
Hypertension = yes	1.1 [-4.04, 6.24]	2.46 [-2.04, 6.95]	-0.06 [-4.18, 4.06]	-0.01 [-3.8, 3.79]	2.57 [-1.99, 7.13]	1.53 [-2.55, 5.6]	0.74 [-2.69, 4.17]
**Septal segments**							
Hypertension = no	-2.39 [-5.17, 0.38]	-0.17 [-2.64, 2.29]	-1.8 [-4.22, 0.61]	-1.16 [-3.4, 1.07]	-0.69 [-3.17, 1.79]	-1.89 [-4.33, 0.54]	-1.06 [-3.09, 0.96]
Hypertension = yes	-1.64 [-5.87, 2.59]	0.43 [-3.3, 4.15]	-2.25 [-5.63, 1.13]	-2.75 [-5.84, 0.35]	-0.64 [-4.41, 3.14]	-1.09 [-4.46, 2.27]	-1.92 [-4.73, 0.89]
**RIGHT VENTRICLE**							
**End-diastolic volume**							
Hypertension = no	0.45 [-4.24, 5.13]	0.34 [-3.72, 4.41]	1.79 [-2.26, 5.84]	1.85 [-1.91, 5.62]	0.58 [-3.31, 4.48]	3.74 [-0.35, 7.83]	1.01 [-2.32, 4.34]
Hypertension = yes	-7.43 [-13.82, -1.05]**	-4.97 [-10.58, 0.63]	-2.5 [-7.63, 2.63]	-3.55 [-8.25, 1.15]	-3.95 [-9.61, 1.71]	-2.74 [-7.7, 2.22]	-4.08 [-8.39, 0.22]
**End-systolic volume**							
Hypertension = no	0.78 [-5.99, 7.55]	0.09 [-5.79, 5.97]	2.12 [-3.74, 7.97]	2.36 [-3.09, 7.81]	0.74 [-4.89, 6.37]	4.78 [-1.14, 10.69]	1.45 [-3.36, 6.27]
Hypertension = yes	-12.56 [-20.77, -4.35]**	-8.16 [-15.42, -0.9]**	-6.7 [-13.31, -0.09]**	-6.19 [-12.28, -0.1]**	-7.24 [-14.57, 0.09]	-7.13 [-13.5, -0.75]**	-6.1 [-11.69, -0.5]**
**Stroke volume**							
Hypertension = no	0.07 [-4.58, 4.73]	0.5 [-3.55, 4.54]	1.44 [-2.59, 5.47]	1.31 [-2.44, 5.06]	0.35 [-3.52, 4.22]	2.72 [-1.36, 6.81]	0.52 [-2.8, 3.83]
Hypertension = yes	-2.73 [-9.66, 4.2]	-2.11 [-8.14, 3.92]	1.36 [-4.11, 6.84]	-1.14 [-6.19, 3.9]	-0.95 [-7.02, 5.12]	1.3 [-3.99, 6.6]	-2.26 [-6.9, 2.37]
**Ejection fraction**							
Hypertension = no	-0.18 [-3.3, 2.93]	0.45 [-2.26, 3.15]	-0.05 [-2.75, 2.65]	-0.43 [-2.94, 2.08]	-0.12 [-2.71, 2.47]	-0.39 [-3.14, 2.35]	-0.38 [-2.6, 1.83]

Hypertension = yes	4.69 [0.92, 8.46]**	2.83 [-0.49, 6.15]	3.84 [0.88, 6.81]**	2.42 [-0.36, 5.19]	3.32 [-0.01, 6.64]	3.71 [0.84, 6.58]**	1.93 [-0.64, 4.49]
**VESSLES**							
**Diameter ascending Aorta**							
Hypertension = no	2.68 [0.15, 5.21]**	1.83 [-0.45, 4.11]	2.43 [0.22, 4.63]**	2.8 [0.8, 4.8]**	2.57 [0.38, 4.76]**	1.48 [-0.72, 3.67]	2.83 [1.02, 4.64]
Hypertension = yes	-1.68 [-4.75, 1.39]	-2.31 [-4.99, 0.36]	-1.16 [-3.67, 1.35]	-0.95 [-3.2, 1.31]	-1.61 [-4.31, 1.08]	-1.06 [-3.4, 1.29]	-1.01 [-3.05, 1.02]
**Maximum diameter infrarenal**							
Hypertension = no	-2.09 [-4.64, 0.45]	-1.66 [-3.95, 0.62]	-0.81 [-3.04, 1.42]	-0.61 [-2.65, 1.43]	-1.45 [-3.66, 0.76]	-0.33 [-2.54, 1.88]	-0.97 [-2.82, 0.88]
Hypertension = yes	-0.48 [-3.78, 2.82]	-0.66 [-3.55, 2.23]	-0.35 [-3.04, 2.35]	-0.74 [-3.15, 1.67]	1.16 [-1.73, 4.06]	-0.84 [-3.34, 1.67]	-0.33 [-2.52, 1.86]
**Diameter pulmonary trunk**							
Hypertension = no	1.02 [-1.88, 3.93]	1.88 [-0.72, 4.47]	1.37 [-1.16, 3.91]	2.47 [0.18, 4.76]**	2.64 [0.15, 5.14]**	1.59 [-0.91, 4.09]	2.65 [0.58, 4.72]**
Hypertension = yes	-2.91 [-6.4, 0.57]	-3.29 [-6.32, -0.25]**	-2.04 [-4.9, 0.82]	-2.81 [-5.35, -0.28]**	-3.25 [-6.29, -0.2]**	-5 [-7.54, -2.46]**	-2.15 [-4.46, 0.16]
**Diameter right pulmonary artery**							
Hypertension = no	0.24 [-3.25, 3.73]	0.51 [-2.61, 3.64]	-0.2 [-3.25, 2.84]	0.32 [-2.46, 3.09]	1.45 [-1.57, 4.47]	-0.28 [-3.29, 2.73]	0.87 [-1.65, 3.38]
Hypertension = yes	0.03 [-3.82, 3.87]	-0.29 [-3.67, 3.08]	1.24 [-1.9, 4.37]	0.06 [-2.76, 2.88]	-1.91 [-5.28, 1.45]	-0.3 [-3.23, 2.63]	-0.68 [-3.24, 1.88]
**Diameter left pulmonary artery**							
Hypertension = no	0.2 [-2.51, 2.92]	0.84 [-1.59, 3.27]	0.71 [-1.65, 3.08]	0.4 [-1.76, 2.56]	0.31 [-2.05, 2.66]	-0.14 [-2.48, 2.2]	0.75 [-1.22, 2.71]
Hypertension = yes	-3.3 [-6.68, 0.07]	-1.88 [-4.86, 1.1]	-1.26 [-4.05, 1.52]	-2.28 [-4.75, 0.2]	-2.98 [-5.94, -0.01]**	-3.28 [-5.83, -0.74]**	-2.3 [-4.54, -0.06]**

Table S11: Cardiovascular outcomes and long-term exposure to air pollution, stratified by hypertension – percent change and confidence intervals of the outcome mean per interquartile range increase in the air pollutant of interest. Main model: adjusted for age, sex, height, body mass index, income, marital status, education years and smoking. Significant results with p-value <0.05 are marked with **.

Supplement Table S12 – Air pollution and adipose tissue outcomes

Outcome	PM_{10}	PM_{coarse}	$PM_{2.5}$	PNC	NO_2	NO_x	$PM_{25}abs$
	%-change [95%-CI]	*%-change [95%-CI]*	*%-change [95%-CI]*	*%-change [95%-CI]*	*%-change [95%-CI]*	*%-change [95%-CI]*	*%-change [95%-CI]*
**Total adipose tissue**							
Basic model	1.15 [-5.19, 7.49]	1.56 [-4.80, 7.92]	0.96 [-5.11, 7.03]	1.25 [-3.85, 6.36]	-0.81 [-6.92, 5.29]	1.02 [-4.62, 6.66]	2.37 [-4.76, 9.50]
Main model	2.02 [-4.23, 8.28]	2.63 [-3.73, 8.98]	0.76 [-5.28, 6.79]	1.64 [-3.40, 6.68]	-0.25 [-6.33, 5.82]	1.07 [-4.53, 6.67]	3.76 [-3.28, 10.80]
**Visceral adipose tissue**							
Basic model	1.64 [-5.64, 8.92]	2.83 [-4.47, 10.13]	2.53 [-4.43, 9.50]	2.27 [-3.59, 8.13]	1.90 [-5.11, 8.91]	2.32 [-4.15, 8.80]	4.19 [-3.99, 12.37]
Main model	2.76 [-4.50, 10.02]	4.34 [-3.04, 11.71]	2.47 [-4.54, 9.47]	3.33 [-2.52, 9.17]	2.75 [-4.30, 9.79]	3.00 [-3.50, 9.50]	5.61 [-2.56, 13.78]
**Subcutaneous adipose tissue**							
Basic model	0.88 [-5.82, 7.58]	0.85 [-5.87, 7.58]	0.09 [-6.33, 6.50]	0.69 [-4.70, 6.08]	-2.32 [-8.78, 4.13]	0.30 [-5.67, 6.26]	1.36 [-6.17, 8.90]
Main model	1.62 [-5.03, 8.27]	1.67 [-5.09, 8.44]	-0.20 [-6.61, 6.22]	0.71 [-4.66, 6.07]	-1.92 [-8.37, 4.53]	0.00 [-5.95, 5.96]	2.74 [-4.76, 10.23]
**Epi – and pericardial adipose tissue**							
Basic model	7.19 [-0.46, 14.84]*	5.94 [-1.74, 13.63]	-0.04 [-7.48, 7.40]	2.97 [-3.11, 9.04]	3.20 [-4.24, 10.65]	2.60 [-4.23, 9.42]	7.44 [-1.13, 16.01]*
Main model	8.84 [1.19, 16.49]**	7.13 [-0.56, 14.83]*	0.06 [-7.41, 7.53]	4.33 [-1.72, 10.39]	4.45 [-3.03, 11.92]	4.11 [-2.72, 10.93]	8.76 [0.18, 17.34]**
**Systolic epicardial adipose tissue**							
Basic model	5.68 [-1.79, 13.14]	3.07 [-4.44, 10.57]	-2.13 [-9.37, 5.11]	3.65 [-2.26, 9.56]	0.92 [-6.34, 8.18]	2.91 [-3.73, 9.55]	3.87 [-4.50, 12.24]
Main model	5.15 [-2.55, 12.85]	2.87 [-4.87, 10.61]	-3.23 [-10.70, 4.25]	2.85 [-3.22, 8.92]	1.05 [-6.44, 8.55]	2.47 [-4.37, 9.31]	4.21 [-4.42, 12.84]
**Diastolic epicardial adipose tissue**							
Basic model	4.96 [-2.57, 12.49]	2.68 [-4.89, 10.24]	-2.20 [-9.49, 5.10]	2.62 [-3.35, 8.58]	0.71 [-6.60, 8.03]	1.90 [-4.81, 8.60]	3.84 [-4.59, 12.27]
Main model	4.72 [-2.94, 12.37]	2.55 [-5.13, 10.24]	-3.33 [-10.75, 4.09]	2.01 [-4.02, 8.05]	0.97 [-6.48, 8.41]	1.61 [-5.19, 8.40]	4.74 [-3.83, 13.31]
**Systolic pericardial adipose tissue**							
Basic model	9.25 [1.83, 16.66]**	5.00 [-2.49, 12.49]	0.43 [-6.81, 7.67]	5.40 [-0.50, 11.29]*	2.14 [-5.12, 9.39]	4.67 [-1.96, 11.30]	5.98 [-2.37, 14.33]
Main model	8.57 [0.98, 16.17]**	4.37 [-3.29, 12.03]	0.20 [-7.22, 7.61]	5.25 [-0.75, 11.25]*	2.04 [-5.39, 9.47]	4.65 [-2.12, 11.41]	6.24 [-2.29, 14.78]
**Diastolic pericardial adipose tissue**							
Basic model	9.22 [1.68, 16.75]**	5.35 [-2.25, 12.95]	0.14 [-7.21, 7.49]	5.19 [-0.80, 11.17]*	1.99 [-5.38, 9.35]	4.41 [-2.33, 11.14]	6.17 [-2.31, 14.65]
Main model	8.53 [0.84, 16.23]**	4.85 [-2.91, 12.60]	-0.38 [-7.88, 7.13]	5.04 [-1.04, 11.12]	1.84 [-5.68, 9.37]	4.34 [-2.51, 11.20]	6.41 [-2.24, 15.05]
**Mean adipose content of the liver**							
Basic model	5.53 [-6.49, 18.27]	4.42 [-7.61, 17.47]	7.01 [-4.78, 19.82]	1.75 [-8.05, 11.55]	3.50 [-8.22, 15.44]	1.90 [-8.96, 12.88]	12.60 [-2.94, 37.07]
Main model	7.47 [-4.61, 20.25]	8.25 [-4.18, 21.74]	8.64 [-3.31, 21.63]	4.10 [-5.70, 13.90]	6.25 [-5.56, 18.29]	4.59 [-6.29, 15.60]	14.96 [-1.45, 40.80]*

**Mean adipose content of the pancreas**							
Basic model	5.53 [-6.49, 18.27]	4.42 [-7.61, 17.47]	7.01 [-4.78, 19.82]	1.75 [-8.05, 11.55]	3.50 [-8.22, 15.44]	1.90 [-8.96, 12.88]	12.60 [-2.94, 37.07]
Main model	7.47 [-4.61, 20.25]	8.25 [-4.18, 21.74]	8.64 [-3.31, 21.63]	4.10 [-5.70, 13.90]	6.25 [-5.56, 18.29]	4.59 [-6.29, 15.60]	14.96 [-1.45, 40.80]
**Renal hilus adipose tissue**							
Basic model	1.41 [-4.42, 7.25]	2.53 [-3.39, 8.45]	4.48 [-0.98, 9.95]	3.92 [-0.70, 8.55]*	1.01 [-4.59, 6.61]	3.64 [-1.48, 8.76]	1.40 [-5.01, 7.81]
Main model	2.25 [-3.61, 8.11]	4.40 [-1.64, 10.44]	4.51 [-1.03, 10.05]	4.60 [-0.06, 9.26]*	2.13 [-3.56, 7.82]	4.21 [-0.96, 9.39]	3.45 [-3.04, 9.95]

Table S12: Adipose tissue outcomes and long-term exposure to air pollution – percent change and confidence intervals of the outcome mean per interquartile range increase in the air pollutant of interest. Main model: adjusted for age, sex, height, income, and physical activity. Significant association ($p > 0.05$): **. Trend ($p < 0.1$): *

Supplement Table S13 – Air pollution and adipose tissue outcomes, stratified by age

Outcome	PM_{10}	PM_{Coarse}	$PM_{2.5}$	PNC	NO_2	NO_X	$PM_{25}abs$
Stratification	*%-change [95%-CI]*	*%-change [95%-CI]*	*%-change [95%-CI]*	*%-change [95%-CI]*	*%-change [95%-CI]*	*%-change [95%-CI]*	*%-change [95%-CI]*
**Total adipose tissue**							
Age <65 years	1.50 [-5.44; 8.45]	2.59 [-4.49; 9.66]	0.81 [-6.27; 7.88]	1.58 [-4.08; 7.24]	-0.59 [-7.54; 6.35]	0.80 [-5.52; 7.12]	3.86 [-4.17; 11.90]
Age ≥65 years	4.85 [-11.46; 21.16]	2.05 [-13.67; 17.77]	-2.04 [-15.52; 11.44]	-0.45 [-13.94; 13.04]	-1.23 [-15.24; 12.77]	-1.59 [-16.09; 12.90]	-0.15 [-16.68; 16.38]
**Visceral adipose tissue**							
Age <65 years	2.91 [-4.97; 10.78]	5.44 [-2.56; 13.44]	3.27 [-4.74; 11.28]	3.46 [-2.94; 9.87]	3.88 [-3.98; 11.75]	3.56 [-3.59; 10.72]	6.18 [-2.91; 15.28]
Age ≥65 years	-1.22 [-21.70; 19.27]	-3.30 [-22.99; 16.39]	-4.74 [-21.60; 12.12]	-2.95 [-19.83; 13.93]	-6.91 [-24.37; 10.56]	-6.67 [-24.76; 11.41]	-4.37 [-25.04; 16.31]
**Subcutaneous adipose tissue**							
Age <65 years	0.73 [-6.74; 8.19]	1.00 [-6.60; 8.60]	-0.56 [-8.15; 7.04]	0.54 [-5.54; 6.61]	-3.08 [-10.53; 4.37]	-0.74 [-7.52; 6.05]	2.57 [-6.06; 11.21]
Age ≥65 years	8.22 [-8.44; 24.89]	5.02 [-11.08; 21.13]	-0.54 [-14.39; 13.31]	0.94 [-12.91; 14.79]	1.92 [-12.45; 16.29]	1.23 [-13.65; 16.11]	2.19 [-14.77; 19.15]
**Epi – and pericardial adipose tissue**							
Age <65 years	9.57 [1.41; 17.72]**	8.98 [0.76; 17.21]**	1.03 [-7.27; 9.34]	4.88 [-1.69; 11.44]	7.77 [-0.46; 16.01]	5.53 [-1.85; 12.91]	11.91 [2.55; 21.27]**
Age ≥65 years	-0.26 [-22.81; 22.29]	-7.06 [-28.65; 14.53]	-7.09 [-26.90; 12.72]	-5.11 [-22.20; 11.98]	-13.56 [-32.46; 5.35]	-10.05 [-29.29; 9.18]	-11.23 [-34.41; 11.94]
**Systolic epicardial adipose tissue**							
Age <65 years	8.35 [0.00; 16.70]**	4.76 [-3.69; 13.22]	-1.23 [-9.70; 7.25]	6.14 [-0.55; 12.82]	3.62 [-4.84; 12.07]	6.10 [-1.42; 13.63]	7.68 [-1.95; 17.30]
Age ≥65 years	-13.16 [-33.36; 7.03]	-8.42 [-27.97; 11.13]	-14.50 [-32.17; 3.17]	-15.38 [-30.43; -0.33]	-11.88 [-29.07; 5.30]	-17.52 [-34.55; -0.48]**	-15.65 [-36.47; 5.17]
**Diastolic epicardial adipose tissue**							
Age <65 years	7.42 [-0.77; 15.61]	3.82 [-4.47; 12.11]	-1.72 [-10.02; 6.58]	4.68 [-1.89; 11.25]	3.55 [-4.74; 11.83]	4.89 [-2.49; 12.28]	7.57 [-1.86; 17.00]
Age ≥65 years	-11.78 [-32.96; 9.41]	-6.66 [-27.15; 13.83]	-13.97 [-32.50; 4.56]	-14.31 [-30.15; 1.54]	-12.43 [-30.40; 5.53]	-17.73 [-35.58; 0.12]	-13.28 [-35.18; 8.62]
**Systolic pericardial adipose tissue**							
Age <65 years	8.25 [-0.17; 16.66]	5.12 [-3.40; 13.63]	-0.44 [-8.98; 8.10]	5.57 [-1.17; 12.32]	3.57 [-4.95; 12.09]	5.65 [-1.94; 13.24]	8.68 [-1.00; 18.37]
Age ≥65 years	6.70 [-13.02; 26.41]	-1.77 [-20.78; 17.23]	-3.12 [-20.55; 14.32]	-0.63 [-15.67; 14.41]	-7.56 [-24.31; 9.20]	-5.07 [-22.05; 11.92]	-9.67 [-30.02; 10.67]

Outcome	PM_{10}	PM_{Coarse}	$PM_{2.5}$	PNC	NO_2	NO_X	$PM_{25}abs$
Stratification	*%-change [95%-CI]*	*%-change [95%-CI]*	*%-change [95%-CI]*	*%-change [95%-CI]*	*%-change [95%-CI]*	*%-change [95%-CI]*	*%-change [95%-CI]*
**Diastolic pericardial adipose tissue**							
Age <65 years	8.30 [-0.14; 16.75]	5.59 [-2.95; 14.13]	-0.70 [-9.27; 7.87]	5.56 [-1.21; 12.33]	3.65 [-4.89; 12.20]	5.68 [-1.94; 13.29]	8.97 [-0.75; 18.69]
Age ≥65 years	5.73 [-14.85; 26.31]	-1.84 [-21.66; 17.97]	-4.96 [-23.11; 13.20]	-1.54 [-17.22; 14.13]	-8.74 [-26.18; 8.70]	-6.57 [-24.25; 11.10]	-10.22 [-31.42; 10.99]
**Mean adipose content of the liver**							
Age <65 years	6.73 [-6.40; 20.70]	7.37 [-6.16; 22.18]	8.15 [-5.31; 22.97]	3.81 [-7.00; 14.62]	4.84 [-8.45; 18.43]	4.60 [-7.38; 16.73]	9.08 [-6.49; 34.95]
Age ≥65 years	18.27 [-14.04; 55.90]	15.16 [-15.73; 53.17]	13.29 [-14.39; 47.06]	8.94 [-17.42; 35.31]	13.17 [-14.44; 42.08]	6.92 [-22.41; 37.15]	53.94 [-2.05; 222.14]
**Mean adipose content of the pancreas**							
Age <65 years	-4.25 [-15.57; 7.74]	-5.76 [-17.04; 6.47]	-5.84 [-16.94; 6.25]	-6.21 [-15.96; 3.54]	-6.44 [-18.25; 5.60]	-3.86 [-14.62; 7.02]	-5.55 [-14.57; 8.74]
Age ≥65 years	-10.62 [-34.32; 16.29]	-13.89 [-35.23; 11.41]	-15.24 [-34.01; 6.84]	-6.93 [-28.72; 14.87]	-10.02 [-32.07; 12.88]	-11.64 [-35.33; 12.66]	-4.06 [-19.79; 35.91]
**Renal hilus adipose tissue**							
Age <65 years	1.70 [-4.59; 7.99]	4.24 [-2.26; 10.74]	6.31 [0.09; 12.52]	5.25 [0.23; 10.26]	3.55 [-2.70; 9.81]	5.65 [0.07; 11.23]**	3.41 [-3.65; 10.48]
Age ≥65 years	2.14 [-15.15; 19.42]	0.78 [-16.55; 18.12]	-2.86 [-17.18; 11.45]	-0.91 [-15.69; 13.88]	-8.82 [-23.93; 6.29]	-7.95 [-23.79; 7.89]	0.32 [-18.52; 19.16]

Table S13: Adipose tissue outcomes and long-term exposure to air pollution, stratified by age – percent change and confidence intervals of the outcome mean per interquartile range increase in the air pollutant of interest. Main model: adjusted for sex, height, income, and physical activity. Significant results with p-value <0.05 are marked with **.

Supplement Table S14 – Air pollution and adipose tissue outcomes, stratified by sex

**Outcome**	**PM_{10}**	**PM_{Coarse}**	**$PM_{2.5}$**	**PNC**	**NO_2**	**NO_X**	**$PM_{25}abs$**
Stratification	*%-change [95%-CI]*	*%-change [95%-CI]*	*%-change [95%-CI]*	*%-change [95%-CI]*	*%-change [95%-CI]*	*%-change [95%-CI]*	*%-change [95%-CI]*
**Total adipose tissue**							
Female	1.76 [-7.96; 11.48]	3.57 [-7.07; 14.22]	-0.35 [-11.22; 10.52]	3.34 [-4.29; 10.97]	2.21 [-8.43; 12.84]	3.35 [-5.58; 12.28]	6.68 [-5.21; 18.56]
Male	7.06 [-1.91; 16.04]	4.05 [-4.29; 12.39]	4.65 [-3.10; 12.40]	4.32 [-2.89; 11.54]	2.54 [-5.48; 10.57]	3.80 [-4.03; 11.63]	6.71 [-2.73; 16.14]
**Visceral adipose tissue**							
Female	3.87 [-5.55; 13.29]	9.58 [-0.71; 19.87]	3.77 [-6.82; 14.36]	5.34 [-1.94; 12.62]	6.72 [-3.47; 16.90]	5.40 [-3.17; 13.97]	9.78 [-1.57; 21.14]
Male	6.01 [-6.02; 18.04]	3.04 [-8.10; 14.18]	3.88 [-6.48; 14.25]	4.57 [-5.09; 14.23]	2.35 [-8.38; 13.09]	3.81 [-6.67; 14.30]	5.36 [-7.29; 18.02]
**Subcutaneous adipose tissue**							
Female	-0.04 [-11.24; 11.15]	-0.24 [-12.52; 12.04]	-3.53 [-16.04; 8.97]	1.21 [-7.60; 10.02]	-1.60 [-13.86; 10.65]	0.85 [-9.46; 11.16]	3.66 [-10.08; 17.40]
Male	7.97 [-0.62; 16.56]	4.87 [-3.12; 12.86]	5.02 [-2.40; 12.45]	4.45 [-2.46; 11.37]	2.67 [-5.03; 10.36]	3.94 [-3.56; 11.45]	7.71 [-1.31; 16.74]
**Epi – and pericardial adipose tissue**							
Female	7.52 [-1.15; 16.19]	8.18 [-1.40; 17.77]	4.69 [-5.13; 14.51]	5.93 [-0.81; 12.68]	9.91 [0.54; 19.27]**	6.59 [-1.34; 14.51]	12.84 [2.42; 23.27]**
Male	9.79 [-2.34; 21.93]	6.57 [-4.71; 17.86]	-2.49 [-13.08; 8.10]	1.79 [-8.00; 11.59]	1.37 [-9.55; 12.30]	0.69 [-9.94; 11.32]	6.21 [-6.56; 18.97]
**Systolic epicardial adipose tissue**							
Female	4.86 [-4.18; 13.89]	5.89 [-4.08; 15.87]	0.06 [-10.14; 10.25]	2.52 [-4.53; 9.58]	8.20 [-1.55; 17.95]	3.44 [-4.83; 11.71]	8.41 [-2.53; 19.36]
Male	5.23 [-6.71; 17.16]	1.79 [-9.29; 12.88]	-4.27 [-14.63; 6.09]	2.66 [-6.93; 12.24]	-2.50 [-13.20; 8.19]	0.76 [-9.65; 11.17]	2.24 [-10.28; 14.76]
**Diastolic epicardial adipose tissue**							
Female	3.63 [-5.54; 12.80]	6.83 [-3.25; 16.92]	1.63 [-8.69; 11.95]	1.08 [-6.07; 8.23]	7.13 [-2.77; 17.04]	2.04 [-6.35; 10.43]	6.65 [-4.47; 17.77]
Male	5.84 [-5.97; 17.64]	0.64 [-10.33; 11.61]	-5.60 [-15.84; 4.64]	2.60 [-6.88; 12.09]	-1.83 [-12.41; 8.76]	0.47 [-9.83; 10.76]	4.49 [-7.89; 16.87]
**Systolic pericardial adipose tissue**							
Female	4.14 [-2.79; 11.06]	5.27 [-2.36; 12.91]	3.27 [-4.53; 11.07]	2.74 [-2.66; 8.14]	5.50 [-2.00; 13.00]	3.88 [-2.44; 10.20]	6.16 [-2.24; 14.56]

Outcome	PM_{10}	PM_{Coarse}	$PM_{2.5}$	PNC	NO_2	NO_X	PM_{25}abs
Stratification	*%-change [95%-CI]*	*%-change [95%-CI]*	*%-change [95%-CI]*	*%-change [95%-CI]*	*%-change [95%-CI]*	*%-change [95%-CI]*	*%-change [95%-CI]*
Male	10.87 [-1.77; 23.52]	2.96 [-8.84; 14.77]	-1.37 [-12.42; 9.69]	6.05 [-4.14; 16.23]	-0.78 [-12.18; 10.62]	3.35 [-7.73; 14.43]	5.38 [-7.95; 18.70]
**Diastolic pericardial adipose tissue**							
Female	3.66 [-3.42; 10.73]	5.30 [-2.49; 13.10]	4.09 [-3.86; 12.03]	2.00 [-3.51; 7.52]	5.29 [-2.37; 12.95]	3.30 [-3.16; 9.77]	5.74 [-2.84; 14.33]
Male	10.86 [-1.90; 23.61]	3.42 [-8.49; 15.32]	-2.92 [-14.06; 8.22]	6.03 [-4.25; 16.30]	-1.20 [-12.70; 10.30]	2.91 [-8.26; 14.09]	5.64 [-7.79; 19.08]
**Mean adipose content of the liver**							
Female	5.68 [-11.46; 24.31]	20.16 [-0.19; 43.26]	6.61 [-12.25; 28.26]	6.66 [-7.23; 20.54]	5.16 [-12.98; 23.85]	5.51 [-10.53; 21.81]	11.65 [-9.24; 53.57]
Male	7.37 [-9.53; 25.70]	0.55 [-14.88; 17.75]	11.34 [-4.08; 28.50]	1.15 [-13.00; 15.29]	7.96 [-7.97; 24.32]	3.43 [-11.75; 18.85]	17.72 [-4.11; 57.95]
**Mean adipose content of the pancreas**							
Female	-8.88 [-22.17; 5.34]	-3.53 [-18.38; 12.99]	-7.36 [-21.65; 8.64]	-6.97 [-18.44; 4.50]	-5.71 [-20.50; 9.45]	-2.87 [-16.08; 10.52]	-6.45 [-16.78; 11.95]
Male	-4.01 [-19.09; 12.27]	-9.94 [-23.40; 4.96]	-11.74 [-24.02; 1.84]	-8.38 [-21.61; 4.84]	-9.43 [-23.95; 5.44]	-10.33 [-24.32; 3.86]	-6.49 [-16.78; 11.81]
**Renal hilus adipose tissue**							
Female	-1.63 [-9.26; 6.00]	-2.22 [-10.71; 6.27]	-0.11 [-8.01; 7.80]	1.48 [-4.53; 7.50]	-2.63 [-10.47; 5.22]	0.50 [-6.35; 7.35]	-0.16 [-8.94; 8.61]
Male	5.20 [-3.68; 14.08]	8.83 [0.28; 17.38]	7.57 [-0.23; 15.37]	7.41 [0.24; 14.58]	5.55 [-2.71; 13.80]	7.26 [-0.53; 15.06]	6.21 [-3.30; 15.72]

Table S14: Adipose tissue outcomes and long-term exposure to air pollution, stratified by sex – percent change and confidence intervals of the outcome mean per interquartile range increase in the air pollutant of interest. Main model: adjusted for age, height, income, and physical activity. Significant results with p-value <0.05 are marked with **.

Supplement Table S15 – Air pollution and adipose tissue outcomes, stratified by diabetes status

Outcome	PM_{10}	PM_{Coarse}	$PM_{2.5}$	PNC	NO_2	NO_X	$PM_{25}abs$
Stratification	*%-change [95%-CI]*	*%-change [95%-CI]*	*%-change [95%-CI]*	*%-change [95%-CI]*	*%-change [95%-CI]*	*%-change [95%-CI]*	*%-change [95%-CI]*
**Total adipose tissue**							
Diabetes	20.20 [7.11; 33.29]**	15.33 [1.91; 28.75]**	8.65 [-8.70; 26.00]	15.81 [4.84; 26.79]**	13.04 [-1.05; 27.12]	16.04 [3.12; 28.96]**	18.16 [2.26; 34.06]**
Prediabetes	2.75 [-11.66; 17.15]	1.57 [-11.05; 14.19]	-0.89 [-12.37; 10.59]	4.87 [-7.06; 16.79]	0.16 [-12.45; 12.77]	2.14 [-10.31; 14.59]	2.72 [-11.68; 17.12]
Normoglycemia	-0.43 [-8.02; 7.17]	-0.06 [-8.02; 7.90]	-2.39 [-10.26; 5.48]	-1.10 [-7.06; 4.85]	-2.50 [-10.12; 5.12]	-2.07 [-8.91; 4.77]	-0.82 [-9.57; 7.93]
**Visceral adipose tissue**							
Diabetes	22.67 [5.89; 39.46]**	13.03 [-4.91; 30.96]	11.25 [-8.83; 31.33]	17.78 [4.25; 31.30]**	16.16 [-1.35; 33.66]	18.78 [3.58; 33.98]**	18.46 [-1.49; 38.40]
Prediabetes	2.57 [-12.75; 17.89]	6.90 [-7.10; 20.91]	-3.81 [-17.32; 9.70]	5.67 [-6.80; 18.14]	4.91 [-9.22; 19.04]	5.04 [-8.77; 18.85]	4.86 [-11.20; 20.92]
Normoglycemia	-2.90 [-12.68; 6.88]	0.10 [-10.16; 10.36]	-2.09 [-12.23; 8.06]	-1.27 [-8.94; 6.41]	-2.71 [-12.53; 7.11]	-1.69 [-10.51; 7.12]	-5.64 [-16.89; 5.61]
**Subcutaneous adipose tissue**							
Diabetes	18.31 [3.68; 32.93]**	16.61 [2.18; 31.03]**	6.66 [-12.14; 25.46]	13.90 [1.63; 26.16]**	11.00 [-4.44; 26.44]	13.08 [-1.33; 27.49]	17.48 [0.12; 34.84]**
Prediabetes	0.72 [-15.44; 16.87]	-3.11 [-17.23; 11.01]	0.16 [-12.71; 13.02]	1.91 [-11.50; 15.32]	-4.37 [-18.46; 9.71]	-1.13 [-15.09; 12.82]	-0.09 [-16.23; 16.06]
Normoglycemia	0.94 [-7.30; 9.19]	-0.15 [-8.80; 8.50]	-2.55 [-11.10; 5.99]	-1.01 [-7.48; 5.46]	-2.38 [-10.66; 5.90]	-2.28 [-9.71; 5.15]	1.86 [-7.65; 11.37]
**Epi – and pericardial adipose tissue**							
Diabetes	18.48 [-3.73; 40.70]	7.89 [-15.09; 30.88]	-2.28 [-27.89; 23.32]	13.27 [-4.68; 31.23]	8.94 [-13.81; 31.70]	13.37 [-6.73; 33.48]	10.84 [-15.07; 36.75]
Prediabetes	13.69 [-2.96; 30.34]	16.28 [0.81; 31.75]**	-0.23 [-15.64; 15.17]	9.06 [-4.79; 22.90]	13.18 [-2.63; 28.98]	11.57 [-3.67; 26.80]	14.86 [-2.82; 32.55]
Normoglycemia	0.68 [-8.48; 9.85]	1.59 [-7.93; 11.12]	-5.07 [-14.50; 4.37]	-2.56 [-9.72; 4.60]	-1.91 [-11.05; 7.22]	-3.92 [-12.13; 4.30]	-2.28 [-12.75; 8.20]
**Systolic epicardial adipose tissue**							
Diabetes	15.80 [-11.49; 43.09]	5.34 [-22.51; 33.19]	-14.93 [-45.44; 15.58]	11.29 [-10.68; 33.27]	0.81 [-26.87; 28.48]	9.56 [-15.06; 34.18]	14.36 [-16.84; 45.55]
Prediabetes	12.04 [-2.33; 26.41]	11.52 [-1.96; 25.00]	-2.21 [-15.50; 11.09]	5.22 [-6.81; 17.25]	11.07 [-2.59; 24.73]	4.16 [-9.16; 17.47]	15.00 [-0.17; 30.18]

Outcome	PM_{10}	PM_{Coarse}	$PM_{2.5}$	PNC	NO_2	NO_X	$PM_{25}abs$
Stratification	*%-change [95%-CI]*	*%-change [95%-CI]*	*%-change [95%-CI]*	*%-change [95%-CI]*	*%-change [95%-CI]*	*%-change [95%-CI]*	*%-change [95%-CI]*
Normoglycemia	-2.58 [-11.78; 6.63]	-1.16 [-10.74; 8.42]	-2.48 [-11.98; 7.02]	-1.86 [-9.06; 5.34]	-2.52 [-11.70; 6.65]	-1.37 [-9.64; 6.91]	-6.63 [-17.12; 3.86]
**Diastolic epicardial adipose tissue**							
Diabetes	18.95 [-9.45; 47.35]	5.28 [-23.87; 34.43]	-5.73 [-38.01; 26.56]	15.69 [-7.06; 38.45]	4.16 [-24.77; 33.09]	16.44 [-8.97; 41.85]	11.63 [-21.16; 44.42]
Prediabetes	6.61 [-6.58; 19.79]	7.37 [-4.98; 19.71]	-4.92 [-16.94; 7.10]	5.64 [-5.25; 16.53]	5.28 [-7.26; 17.82]	3.46 [-8.63; 15.54]	7.70 [-6.30; 21.69]
Normoglycemia	-0.71 [-10.11; 8.69]	1.29 [-8.48; 11.06]	-3.36 [-13.05; 6.33]	-1.89 [-9.24; 5.46]	-1.04 [-10.41; 8.33]	-1.59 [-10.03; 6.85]	-3.78 [-14.52; 6.95]
**Systolic pericardial adipose tissue**							
Diabetes	33.46 [11.09; 55.84]**	19.69 [-4.49; 43.87]	-0.34 [-28.05; 27.37]	20.32 [1.47; 39.17]**	18.56 [-5.50; 42.63]	19.48 [-1.84; 40.81]	21.15 [-6.28; 48.58]
Prediabetes	14.52 [0.51; 28.52]**	8.25 [-5.13; 21.62]	1.47 [-11.62; 14.55]	10.12 [-1.55; 21.78]	8.12 [-5.41; 21.66]	8.66 [-4.32; 21.65]	16.07 [1.21; 30.93]**
Normoglycemia	-2.15 [-12.37; 8.06]	0.25 [-10.38; 10.87]	-2.09 [-12.63; 8.46]	-0.04 [-8.03; 7.96]	-3.23 [-13.40; 6.95]	0.53 [-8.65; 9.71]	-6.87 [-18.51; 4.77]
**Diastolic pericardial adipose tissue**							
Diabetes	35.63 [13.65; 57.61]**	22.67 [-1.19; 46.54]	-0.57 [-28.24; 27.11]	21.64 [2.97; 40.30]**	20.51 [-3.36; 44.37]	20.48 [-0.71; 41.68]	24.93 [-2.13; 51.98]
Prediabetes	16.26 [1.50; 31.03]**	9.75 [-4.36; 23.86]	0.88 [-12.96; 14.72]	11.18 [-1.14; 23.50]	8.30 [-6.02; 22.63]	9.66 [-4.05; 23.38]	16.62 [0.89; 32.35]**
Normoglycemia	-3.69 [-13.94; 6.56]	-0.44 [-11.12; 10.23]	-2.78 [-13.37; 7.81]	-1.05 [-9.08; 6.98]	-4.05 [-14.27; 6.16]	-0.61 [-9.83; 8.61]	-7.73 [-19.41; 3.96]
**Mean adipose content of the liver**							
Diabetes	19.75 [-13.65; 58.84]	-4.45 [-32.64; 30.51]	9.58 [-21.90; 49.44]	13.32 [-13.13; 39.77]	-0.85 [-32.84; 32.94]	7.16 [-21.68; 36.87]	24.03 [-15.02; 164.94]
Prediabetes	9.64 [-13.42; 35.41]	14.07 [-8.36; 40.05]	-1.48 [-21.79; 22.33]	4.60 [-15.26; 24.46]	9.29 [-12.75; 32.15]	3.97 [-17.97; 26.43]	25.37 [-5.95; 97.44]
Normoglycemia	-1.29 [-15.17; 13.58]	4.40 [-10.89; 21.38]	0.23 [-13.90; 15.93]	-4.73 [-16.46; 7.00]	-0.38 [-14.89; 14.48]	-1.57 [-14.72; 11.77]	-7.01 [-16.87; 10.26]
**Mean adipose content of the pancreas**							
Diabetes	1.51 [-31.29; 40.34]	-11.42 [-39.47; 23.76]	-32.95 [-55.47; -4.42]**	-0.66 [-28.91; 27.60]	-9.62 [-42.74; 25.46]	-7.53 [-37.51; 23.41]	-0.09 [-22.39; 87.57]
Prediabetes	-22.59 [-41.48; -1.59]**	-17.46 [-35.09; 2.88]	-24.43 [-40.48; -5.78]**	-21.86 [-40.75; -2.96]**	-25.03 [-44.82; -4.53]**	-22.65 [-43.02; -1.83]**	-16.97 [-24.19; -0.81]**

Outcome	PM_{10}	PM_{Coarse}	$PM_{2.5}$	PNC	NO_2	NO_X	$PM_{25}abs$
Stratification	*%-change [95%-CI]*	*%-change [95%-CI]*	*%-change [95%-CI]*	*%-change [95%-CI]*	*%-change [95%-CI]*	*%-change [95%-CI]*	*%-change [95%-CI]*
Normoglycemia	-5.60 [-18.28; 7.92]	-4.07 [-17.59; 10.83]	-3.19 [-16.07; 11.03]	-8.41 [-19.30; 2.49]	-3.92 [-17.36; 9.82]	-4.12 [-16.33; 8.26]	-7.91 [-16.88; 7.21]
**Renal hilus adipose tissue**							
Diabetes	22.45 [3.83; 41.07]**	18.41 [-1.33; 38.15]	12.85 [-5.94; 31.63]	24.15 [9.62; 38.68]**	20.88 [1.51; 40.24]**	23.13 [6.85; 39.40]**	23.21 [0.75; 45.66]
Prediabetes	2.40 [-9.36; 14.15]	3.98 [-7.84; 15.81]	0.29 [-10.38; 10.95]	4.21 [-5.94; 14.35]	1.53 [-9.47; 12.54]	2.82 [-8.23; 13.87]	2.71 [-9.56; 14.97]
Normoglycemia	-1.95 [-9.25; 5.35]	3.07 [-4.60; 10.75]	0.65 [-6.63; 7.92]	1.29 [-4.43; 7.01]	-0.65 [-7.84; 6.53]	1.55 [-4.88; 7.99]	-2.56 [-10.70; 5.57]

Table S15: Adipose tissue outcomes and long-term exposure to air pollution, stratified by diabetes status – percent change and confidence intervals of the outcome mean per interquartile range increase in the air pollutant of interest. Main model: adjusted for age, sex, height, income, and physical activity. Significant results with p-value <0.05 are marked with **.

Supplement Table S16 – Air pollution and adipose tissue outcomes, stratified by BMI

Outcome	PM_{10}	PM_{Coarse}	$PM_{2.5}$	PNC	NO_2	NO_X	PM_{25}abs
Stratification	*%-change [95%-CI]*	*%-change [95%-CI]*	*%-change [95%-CI]*	*%-change [95%-CI]*	*%-change [95%-CI]*	*%-change [95%-CI]*	*%-change [95%-CI]*
**Total adipose tissue**							
BMI <30 [kg/m²]	-1.30 [-6.31; 3.72]	0.67 [-4.15; 5.48]	-1.35 [-6.11; 3.41]	-0.83 [-4.68; 3.02]	-1.07 [-5.92; 3.78]	-0.25 [-4.52; 4.03]	-1.91 [-7.34; 3.51]
BMI ≥30 [kg/m²]	1.85 [-6.47; 10.17]	-0.08 [-9.05; 8.90]	3.21 [-4.92; 11.35]	2.10 [-4.81; 9.01]	-0.52 [-8.57; 7.54]	3.43 [-4.45; 11.31]	3.19 [-6.64; 13.02]
**Visceral adipose tissue**							
BMI <30 [kg/m²]	-4.52 [-12.84; 3.80]	1.00 [-7.00; 9.01]	-1.05 [-8.97; 6.86]	-0.42 [-6.82; 5.99]	1.91 [-6.15; 9.98]	1.03 [-6.08; 8.13]	-2.36 [-11.39; 6.66]
BMI ≥30 [kg/m²]	-0.20 [-11.35; 10.96]	0.95 [-11.06; 12.96]	3.94 [-7.02; 14.91]	1.12 [-7.94; 10.18]	-0.56 [-11.34; 10.22]	3.14 [-7.24; 13.52]	0.05 [-13.09; 13.19]
**Subcutaneous adipose tissue**							
BMI <30 [kg/m²]	0.49 [-4.58; 5.56]	0.48 [-4.38; 5.34]	-1.52 [-6.33; 3.29]	-1.06 [-4.95; 2.83]	-2.72 [-7.61; 2.17]	-0.96 [-5.27; 3.36]	-1.66 [-7.15; 3.82]
BMI ≥30 [kg/m²]	1.68 [-8.59; 11.95]	-2.03 [-13.11; 9.04]	1.85 [-8.21; 11.92]	1.32 [-7.22; 9.86]	-1.67 [-11.61; 8.26]	2.14 [-7.61; 11.89]	3.38 [-8.77; 15.52]
**Epi – and pericardial adipose tissue**							
BMI <30 [kg/m²]	1.86 [-7.02; 10.73]	1.31 [-7.15; 9.76]	-2.04 [-10.44; 6.36]	-0.58 [-7.38; 6.21]	3.92 [-4.59; 12.44]	1.34 [-6.20; 8.88]	0.41 [-9.14; 9.96]
BMI ≥30 [kg/m²]	4.86 [-8.06; 17.79]	7.46 [-6.51; 21.44]	-2.40 [-15.28; 10.49]	1.21 [-9.33; 11.75]	0.35 [-12.29; 12.99]	2.18 [-9.89; 14.24]	5.09 [-10.19; 20.38]
**Systolic epicardial adipose tissue**							
BMI <30 [kg/m²]	2.64 [-6.01; 11.28]	2.22 [-6.02; 10.46]	-4.72 [-12.89; 3.44]	-0.07 [-6.69; 6.56]	0.63 [-7.68; 8.94]	-0.10 [-7.45; 7.25]	-0.12 [-9.43; 9.18]
BMI ≥30 [kg/m²]	-0.56 [-14.14; 13.02]	-2.31 [-17.02; 12.40]	-5.95 [-19.42; 7.51]	-1.85 [-12.89; 9.20]	-1.80 [-15.05; 11.44]	-0.38 [-13.04; 12.27]	1.00 [-15.05; 17.05]
**Diastolic epicardial adipose tissue**							
BMI <30 [kg/m²]	2.88 [-5.61; 11.37]	1.32 [-6.78; 9.42]	-2.12 [-10.16; 5.93]	1.26 [-5.24; 7.77]	1.27 [-6.89; 9.44]	2.00 [-5.22; 9.21]	-0.82 [-9.96; 8.32]
BMI ≥30 [kg/m²]	-0.06 [-14.05; 13.93]	-1.05 [-16.21; 14.11]	-8.99 [-22.81; 4.83]	-1.39 [-12.77; 9.99]	-2.61 [-16.25; 11.03]	-1.14 [-14.17; 11.89]	0.59 [-15.95; 17.12]
**Systolic pericardial adipose tissue**							
BMI <30 [kg/m²]	-1.82 [-10.55; 6.92]	-2.75 [-11.07; 5.57]	-5.37 [-13.60; 2.87]	-2.14 [-8.83; 4.54]	-2.92 [-11.30; 5.47]	-1.43 [-8.85; 6.00]	-6.10 [-15.46; 3.26]
BMI ≥30 [kg/m²]	8.14 [-5.33; 21.61]	6.44 [-8.20; 21.09]	1.91 [-11.57; 15.40]	5.70 [-5.28; 16.68]	3.27 [-9.94; 16.48]	7.33 [-5.23; 19.89]	7.96 [-7.99; 23.92]

Outcome	PM_{10}	PM_{Coarse}	$PM_{2.5}$	PNC	NO_2	NO_X	$PM_{25}abs$
Stratification	*%-change [95%-CI]*	*%-change [95%-CI]*	*%-change [95%-CI]*	*%-change [95%-CI]*	*%-change [95%-CI]*	*%-change [95%-CI]*	*%-change [95%-CI]*
**Diastolic pericardial adipose tissue**							
BMI <30 [kg/m²]	-1.59 [-10.36; 7.18]	-1.74 [-10.10; 6.61]	-5.31 [-13.59; 2.96]	-2.46 [-9.17; 4.24]	-2.09 [-10.51; 6.33]	-1.57 [-9.02; 5.88]	-4.93 [-14.34; 4.48]
BMI ≥30 [kg/m²]	7.66 [-6.14; 21.46]	6.39 [-8.61; 21.38]	0.45 [-13.35; 14.26]	5.40 [-5.85; 16.64]	2.01 [-11.52; 15.55]	6.88 [-5.99; 19.74]	6.51 [-9.85; 22.86]
**Mean adipose content of the liver**							
BMI <30 [kg/m²]	2.09 [-13.19; 18.56]	9.05 [-6.50; 26.28]	8.07 [-6.94; 24.77]	-0.80 [-13.28; 11.68]	11.14 [-4.27; 26.94]	4.73 [-8.96; 18.62]	8.05 [-8.55; 37.49]
BMI ≥30 [kg/m²]	-5.20 [-19.94; 10.68]	-6.93 [-22.30; 10.28]	1.40 [-13.77; 18.40]	-3.21 [-16.34; 9.93]	-7.89 [-22.85; 7.47]	-3.68 [-18.34; 11.20]	-2.21 [-15.11; 21.86]
**Mean adipose content of the pancreas**							
BMI <30 [kg/m²]	-7.99 [-20.56; 5.40]	-11.10 [-22.84; 1.71]	-8.45 [-20.00; 4.21]	-9.86 [-20.54; 0.81]	-7.52 [-20.42; 5.68]	-4.48 [-16.16; 7.36]	-11.67 [-18.77; -0.09]
BMI ≥30 [kg/m²]	-15.37 [-29.45; -0.18]**	-13.32 [-28.18; 3.34]	-18.04 [-31.10; -3.42]**	-13.43 [-26.61; -0.25]**	-14.99 [-29.91; 0.33]	-15.21 [-29.76; -0.43]**	-10.79 [-19.79; 6.15]
**Renal hilus adipose tissue**							
BMI <30 [kg/m²]	-0.61 [-7.72; 6.51]	5.17 [-1.97; 12.32]	4.87 [-1.89; 11.64]	3.31 [-2.20; 8.83]	5.54 [-1.42; 12.49]	5.72 [-0.34; 11.79]	3.99 [-3.77; 11.74]
BMI ≥30 [kg/m²]	-1.70 [-11.26; 7.86]	-0.63 [-10.99; 9.72]	1.40 [-7.60; 10.40]	1.41 [-6.59; 9.40]	-3.16 [-12.24; 5.91]	0.09 [-8.71; 8.90]	-4.28 [-15.08; 6.51]

Table S16: Adipose tissue outcomes and long-term exposure to air pollution, stratified by BMI – percent change and confidence intervals of the outcome mean per interquartile range increase in the air pollutant of interest. Main model: adjusted for age, sex, height, income, and physical activity. Significant results with p-value <0.05 are marked with **.

Supplement Table S17 – Air pollution and adipose tissue outcomes, stratified by hs-CRP

Outcome	PM_{10}	PM_{Coarse}	$PM_{2.5}$	PNC	NO_2	NO_X	PM_{25}abs
Stratification	*%-change [95%-CI]*	*%-change [95%-CI]*	*%-change [95%-CI]*	*%-change [95%-CI]*	*%-change [95%-CI]*	*%-change [95%-CI]*	*%-change [95%-CI]*
**Total adipose tissue**							
hsCRP < 1 mg/dl	0.48 [-0.31, 1.27]	0.43 [-0.27, 1.12]	0.50 [-0.20, 1.21]	0.45 [-0.17, 1.08]	0.48 [-0.22, 1.18]	0.29 [-0.43, 1.00]	0.40 [-0.17, 0.96]
hsCRP ≥ 1 mg/dl	0.29 [-0.46, 1.03]	0.08 [-0.60, 0.77]	-0.09 [-0.72, 0.53]	-0.09 [-0.67, 0.49]	0.08 [-0.57, 0.73]	0.12 [-0.49, 0.74]	-0.12 [-0.65, 0.41]
**Visceral adipose tissue**							
hsCRP < 1 mg/dl	1.29 [-2.45, 5.03]	0.97 [-2.30, 4.23]	1.53 [-1.80, 4.85]	1.20 [-1.78, 4.17]	0.40 [-2.92, 3.73]	0.26 [-3.13, 3.65]	0.86 [-1.82, 3.54]
hsCRP ≥ 1 mg/dl	0.52 [-2.41, 3.45]	0.41 [-2.29, 3.12]	-0.31 [-2.77, 2.15]	-0.03 [-2.33, 2.26]	-0.72 [-3.27, 1.83]	0.34 [-2.09, 2.77]	-0.44 [-2.52, 1.63]
**Subcutaneous adipose tissue**							
hsCRP < 1 mg/dl	0.93 [-0.24, 2.09]	0.84 [-0.18, 1.86]	0.87 [-0.17, 1.91]	0.80 [-0.12, 1.73]	1.07 [0.05, 2.10]**	0.60 [-0.46, 1.66]	0.73 [-0.11, 1.56]
hsCRP ≥ 1 mg/dl	0.40 [-0.76, 1.57]	-0.03 [-1.10, 1.05]	-0.28 [-1.26, 0.70]	-0.29 [-1.20, 0.62]	0.32 [-0.69, 1.33]	0.11 [-0.86, 1.07]	-0.26 [-1.08, 0.56]
**Epi – and pericardial adipose tissue**							
hsCRP < 1 mg/dl	2.33 [-11.80, 16.45]	-0.44 [-12.62, 11.74]	2.47 [-10.30, 15.24]	-0.72 [-12.38, 10.95]	3.26 [-9.29, 15.81]	-1.77 [-14.69, 11.15]	0.10 [-10.14, 10.34]
hsCRP ≥ 1 mg/dl	13.83 [1.46, 26.19]**	14.45 [3.05, 25.86]**	7.77 [-2.87, 18.42]	8.39 [-1.35, 18.13]	13.21 [2.22, 24.21]**	2.85 [-7.61, 13.31]	7.06 [-1.70, 15.82]
**Systolic epicardial adipose tissue**							
hsCRP < 1 mg/dl	-0.82 [-15.33, 13.68]	-0.93 [-13.43, 11.57]	-4.59 [-17.68, 8.50]	-1.39 [-13.37, 10.58]	1.62 [-11.28, 14.51]	-4.22 [-17.46, 9.03]	0.46 [-10.05, 10.98]
hsCRP ≥ 1 mg/dl	4.71 [-7.34, 16.76]	4.20 [-6.96, 15.37]	2.25 [-8.05, 12.55]	1.90 [-7.54, 11.35]	5.67 [-5.05, 16.40]	-5.29 [-15.33, 4.75]	1.74 [-6.75, 10.23]
**Diastolic epicardial adipose tissue**							
hsCRP < 1 mg/dl	-1.08 [-15.01, 12.85]	-2.32 [-14.32, 9.69]	-4.61 [-17.18, 7.96]	-4.20 [-15.68, 7.29]	1.67 [-10.71, 14.06]	-5.44 [-18.15, 7.27]	-2.68 [-12.76, 7.41]
hsCRP ≥ 1 mg/dl	6.01 [-6.26, 18.28]	5.50 [-5.86, 16.86]	2.79 [-7.71, 13.28]	2.11 [-7.51, 11.74]	5.61 [-5.32, 16.54]	-4.70 [-14.94, 5.54]	2.22 [-6.43, 10.86]
**Systolic pericardial adipose tissue**							
hsCRP < 1 mg/dl	-4.49 [-19.03, 10.06]	-3.42 [-15.96, 9.12]	-5.25 [-18.39, 7.88]	-2.61 [-14.62, 9.41]	-2.57 [-15.51, 10.37]	-8.69 [-21.92, 4.55]	-1.47 [-12.02, 9.09]

Outcome	PM_{10}	PM_{Coarse}	$PM_{2.5}$	PNC	NO_2	NO_X	$PM_{25}abs$
Stratification	*%-change [95%-CI]*	*%-change [95%-CI]*	*%-change [95%-CI]*	*%-change [95%-CI]*	*%-change [95%-CI]*	*%-change [95%-CI]*	*%-change [95%-CI]*
hsCRP ≥ 1 mg/dl	12.20 [-0.30, 24.71]	9.75 [-1.86, 21.37]	6.92 [-3.82, 17.66]	8.59 [-1.22, 18.40]	14.62 [3.58, 25.66]**	4.93 [-5.59, 15.45]	8.34 [-0.47, 17.14]
**Diastolic pericardial adipose tissue**							
hsCRP < 1 mg/dl	-3.28 [-17.53, 10.97]	-3.44 [-15.72, 8.84]	-4.77 [-17.64, 8.10]	-3.19 [-14.96, 8.57]	-2.22 [-14.90, 10.45]	-9.41 [-22.35, 3.53]	-2.09 [-12.42, 8.25]
hsCRP ≥ 1 mg/dl	12.01 [-0.99, 25.00]	10.64 [-1.41, 22.68]	6.77 [-4.39, 17.93]	8.22 [-1.98, 18.42]	14.44 [2.96, 25.93]**	4.28 [-6.65, 15.21]	8.11 [-1.04, 17.26]
**Mean adipose content of the liver**							
hsCRP < 1 mg/dl	0.91 [-1.52, 3.35]	0.21 [-1.92, 2.33]	1.11 [-1.08, 3.30]	0.69 [-1.27, 2.65]	0.65 [-1.50, 2.80]	0.97 [-1.22, 3.15]	0.28 [-1.49, 2.04]
hsCRP ≥ 1 mg/dl	1.18 [-0.95, 3.32]	0.84 [-1.15, 2.84]	0.21 [-1.61, 2.03]	0.28 [-1.44, 2.00]	0.46 [-1.41, 2.32]	1.03 [-0.76, 2.82]	0.20 [-1.35, 1.75]
**Mean adipose content of the pancreas**							
hsCRP < 1 mg/dl	-1.59 [-4.26, 1.07]	-1.83 [-4.15, 0.49]	-0.99 [-3.39, 1.42]	-0.91 [-3.06, 1.24]	-0.61 [-2.97, 1.75]	-1.02 [-3.42, 1.38]	-1.27 [-3.19, 0.66]
hsCRP ≥ 1 mg/dl	0.55 [-1.51, 2.61]	0.24 [-1.68, 2.17]	-0.10 [-1.86, 1.65]	-0.08 [-1.74, 1.57]	-0.18 [-1.97, 1.62]	-0.50 [-2.23, 1.23]	-0.40 [-1.90, 1.09]
**Renal hilus adipose tissue**							
hsCRP < 1 mg/dl	5.12 [-5.62, 15.86]	3.53 [-5.96, 13.01]	4.47 [-5.29, 14.23]	5.97 [-2.46, 14.39]	0.14 [-9.27, 9.56]	7.73 [-1.75, 17.21]	5.11 [-2.41, 12.63]
hsCRP ≥ 1 mg/dl	3.02 [-6.57, 12.62]	6.06 [-3.23, 15.35]	2.37 [-5.92, 10.66]	4.99 [-2.75, 12.74]	2.82 [-5.85, 11.49]	4.79 [-3.30, 12.87]	5.11 [-1.87, 12.09]

Table S17: Adipose tissue outcomes and long-term exposure to air pollution, stratified by hs-CRP – percent change and confidence intervals of the outcome mean per interquartile range increase in the air pollutant of interest. Main model: adjusted for age, sex, height, income, and physical activity. Significant results with p-value <0.05 are marked with **.

Supplement Table S18 – Air pollution and adipose tissue outcomes, stratified by hypertension

Outcome	PM_{10}	PM_{Coarse}	$PM_{2.5}$	PNC	NO_2	NO_X	$PM_{25}abs$
Stratification	*%-change [95%-CI]*	*%-change [95%-CI]*	*%-change [95%-CI]*	*%-change [95%-CI]*	*%-change [95%-CI]*	*%-change [95%-CI]*	*%-change [95%-CI]*
**Total adipose tissue**							
Hypertension = no	0.08 [-7.51; 7.67]	0.96 [-6.89; 8.81]	-0.89 [-8.73; 6.94]	-0.91 [-7.34; 5.52]	-2.86 [-10.85; 5.13]	-1.76 [-8.99; 5.47]	0.59 [-8.33; 9.50]
Hypertension = yes	12.21 [1.17; 23.25]**	6.13 [-4.51; 16.78]	6.06 [-3.68; 15.80]	10.71 [2.54; 18.89]**	7.50 [-2.18; 17.18]	11.26 [2.06; 20.46]**	13.10 [1.62; 24.57]**
**Visceral adipose tissue**							
Hypertension = no	-3.87 [-13.68; 5.94]	3.15 [-7.06; 13.35]	-0.37 [-10.76; 10.02]	-0.43 [-8.69; 7.82]	-3.05 [-13.49; 7.40]	-0.70 [-10.13; 8.73]	-3.10 [-14.66; 8.47]
Hypertension = yes	16.43 [3.48; 29.38]**	8.91 [-3.67; 21.49]	8.98 [-2.29; 20.25]	13.30 [3.78; 22.82]**	12.77 [1.54; 24.00]**	14.70 [4.12; 25.28]**	17.30 [3.89; 30.70]**
**Subcutaneous adipose tissue**							
Hypertension = no	2.90 [-4.94; 10.75]	0.20 [-7.93; 8.32]	-0.91 [-9.02; 7.20]	-0.75 [-7.40; 5.91]	-2.49 [-10.76; 5.78]	-1.98 [-9.46; 5.50]	3.27 [-5.95; 12.48]
Hypertension = yes	9.42 [-3.18; 22.02]	4.36 [-7.71; 16.43]	3.69 [-7.37; 14.74]	8.71 [-0.64; 18.07]	3.98 [-7.04; 15.00]	8.44 [-2.09; 18.96]	10.11 [-2.99; 23.21]
**Epi – and pericardial adipose tissue**							
Hypertension = no	1.48 [-7.51; 10.47]	2.79 [-6.54; 12.12]	-2.15 [-11.69; 7.39]	0.75 [-6.78; 8.29]	-0.73 [-10.30; 8.83]	1.03 [-7.55; 9.62]	0.16 [-10.36; 10.69]
Hypertension = yes	17.17 [2.95; 31.39]**	13.45 [-0.23; 27.13]	1.68 [-10.78; 14.14]	8.32 [-2.31; 18.95]	9.78 [-2.65; 22.21]	8.22 [-3.62; 20.06]	16.79 [2.03; 31.56]**
**Systolic epicardial adipose tissue**							
Hypertension = no	0.33 [-8.34; 9.00]	1.83 [-7.17; 10.83]	-2.12 [-11.32; 7.07]	-0.90 [-8.16; 6.37]	-2.03 [-11.24; 7.19]	-1.73 [-10.00; 6.55]	-3.34 [-13.48; 6.79]
Hypertension = yes	8.35 [-6.06; 22.76]	2.53 [-11.30; 16.35]	-6.76 [-19.13; 5.61]	4.29 [-6.38; 14.95]	2.32 [-10.18; 14.81]	4.11 [-7.75; 15.97]	11.76 [-3.10; 26.62]
**Diastolic epicardial adipose tissue**							
Hypertension = no	2.19 [-6.60; 10.98]	3.48 [-5.64; 12.61]	-3.39 [-12.72; 5.93]	0.01 [-7.36; 7.38]	-0.53 [-9.88; 8.83]	-1.04 [-9.43; 7.36]	-1.31 [-11.60; 8.99]
Hypertension = yes	6.53 [-8.01; 21.06]	1.41 [-12.51; 15.33]	-5.73 [-18.20; 6.73]	4.19 [-6.55; 14.93]	0.91 [-11.67; 13.50]	4.35 [-7.59; 16.29]	7.91 [-7.13; 22.95]
**Systolic pericardial adipose tissue**							
Hypertension = no	0.99 [-8.60; 10.58]	2.42 [-7.54; 12.38]	0.11 [-10.08; 10.29]	1.86 [-6.17; 9.89]	-0.81 [-11.01; 9.39]	2.97 [-6.18; 12.12]	-1.35 [-12.58; 9.88]

**Outcome**	**PM_{10}**	**PM_{Coarse}**	**$PM_{2.5}$**	**PNC**	**NO_2**	**NO_X**	**PM_{25}abs**
Stratification	*%-change [95%-CI]*	*%-change [95%-CI]*	*%-change [95%-CI]*	*%-change [95%-CI]*	*%-change [95%-CI]*	*%-change [95%-CI]*	*%-change [95%-CI]*
Hypertension = yes	15.86 [2.20; 29.53]**	5.80 [-7.48; 19.09]	-0.28 [-12.25; 11.68]	8.78 [-1.40; 18.97]	3.82 [-8.20; 15.84]	6.94 [-4.45; 18.32]	12.29 [-2.00; 26.58]
**Diastolic pericardial adipose tissue**							
Hypertension = no	0.26 [-9.48; 10.01]	2.10 [-8.02; 12.22]	-0.23 [-10.58; 10.11]	1.23 [-6.93; 9.40]	-1.34 [-11.70; 9.03]	2.53 [-6.77; 11.83]	-1.68 [-13.08; 9.73]
Hypertension = yes	17.05 [3.13; 30.97]**	7.59 [-5.94; 21.13]	-0.94 [-13.15; 11.27]	9.36 [-1.03; 19.74]	4.15 [-8.12; 16.41]	7.19 [-4.43; 18.80]	13.14 [-1.43; 27.71]
**Mean adipose content of the liver**							
Hypertension = no	4.13 [-11.17; 20.61]	11.26 [-5.29; 29.71]	10.57 [-6.26; 29.52]	4.91 [-8.40; 18.22]	4.92 [-11.31; 21.60]	8.23 [-6.87; 23.57]	7.01 [-9.89; 38.11]
Hypertension = yes	12.04 [-8.20; 34.32]	6.40 [-12.77; 28.24]	10.24 [-7.11; 29.85]	4.55 [-10.74; 19.84]	13.28 [-4.75; 31.85]	4.83 [-11.89; 21.84]	31.20 [0.32; 93.51]**
**Mean adipose content of the pancreas**							
Hypertension = no	-12.67 [-24.55; -0.04]**	-12.04 [-24.05; 1.12]	-4.03 [-17.01; 10.32]	-10.69 [-21.85; 0.47]	-12.64 [-25.87; 0.90]	-6.62 [-19.19; 6.13]	-11.78 [-19.07; 0.38]
Hypertension = yes	-0.76 [-19.15; 19.41]	-4.88 [-21.97; 14.49]	-18.30 [-31.60; -3.36]**	-6.77 [-21.40; 7.86]	-4.82 [-21.70; 12.54]	-9.09 [-24.83; 6.92]	0.06 [-14.80; 29.47]
**Renal hilus adipose tissue**							
Hypertension = no	-2.70 [-10.17; 4.76]	1.93 [-5.77; 9.64]	3.49 [-4.04; 11.02]	2.05 [-4.21; 8.30]	1.73 [-5.76; 9.22]	4.65 [-2.25; 11.55]	-1.09 [-9.45; 7.27]
Hypertension = yes	12.04 [1.42; 22.65]**	11.99 [1.12; 22.86]**	7.99 [-1.27; 17.25]	10.63 [2.68; 18.58]**	7.04 [-2.91; 17.00]	7.79 [-1.21; 16.79]	13.00 [1.59; 24.42]**

Table S18: Adipose tissue outcomes and long-term exposure to air pollution, stratified by hypertension – percent change and confidence intervals of the outcome mean per interquartile range increase in the air pollutant of interest. Main model: adjusted for age, sex, height, income, and physical activity. Significant results with p-value <0.05 are marked with **.

Supplement Table S19 – Two-pollutant models and cardiovascular outcomes

Outcome	Second pollutant	PM_{10}	$PM_{2.5}$	PM_{Coarse}	PNC	NO_2	NO_X	$PM_{25}abs$
		%-change [95%-CI]	*%-change [95%-CI]*	*%-change [95%-CI]*	*%-change [95%-CI]*	*%-change [95%-CI]*	*%-change [95%-CI]*	*%-change [95%-CI]*
**LEFT VENTRICLE**								
**End-diastolic volume**	**+PM_{10}**	-	1.78 [-2.08, 5.65]	-0.27 [-5.83, 5.29]	1.45 [-3.24, 6.14]	1.93 [-3.14, 7]	1.97 [-2.59, 6.54]	-2.45 [-8.26, 3.36]
	**+$PM_{2.5}$**	0.02 [-3.92, 3.96]	-	-0.44 [-4.44, 3.55]	0.33 [-3.13, 3.8]	0.5 [-4.21, 5.21]	0.62 [-3.95, 5.2]	-2.03 [-6.49, 2.43]
	**+PM_{Coarse}**	1.18 [-4.38, 6.74]	2.03 [-1.89, 5.95]	-	1.67 [-2.48, 5.82]	2.78 [-2.67, 8.23]	2.26 [-2.1, 6.63]	-1.47 [-6.87, 3.93]
	**+PNC**	-0.51 [-6.33, 5.32]	1.54 [-2.68, 5.76]	-0.9 [-6.05, 4.25]	-	1.18 [-3.99, 6.35]	2.71 [-5.75, 11.17]	-2.71 [-8.04, 2.61]
	**+NO_2**	-0.55 [-5.77, 4.67]	1.43 [-3.32, 6.18]	-1.62 [-7.22, 3.99]	0.36 [-3.92, 4.64]	-	1.13 [-4.51, 6.76]	-6.04 [-13.05, 0.98]
	**+NO_X**	-0.71 [-5.84, 4.42]	1.27 [-3.77, 6.32]	-1.18 [-6.09, 3.72]	-1.17 [-8.83, 6.48]	0.48 [-5.68, 6.63]	-	-3.12 [-8.36, 2.13]
	**+$PM_{25}abs$**	2.81 [-2.71, 8.34]	2.95 [-1.21, 7.11]	1.72 [-3.41, 6.85]	2.67 [-1.4, 6.75]	6.34 [-0.14, 12.83]	3.45 [-1, 7.89]	-
**End-systolic volume**	**+PM_{10}**	-	3.55 [-3.68, 10.78]	-2.34 [-12.74, 8.05]	5.39 [-3.37, 14.15]	4.07 [-5.42, 13.56]	5.12 [-3.41, 13.64]	-0.98 [-11.86, 9.9]
	**+$PM_{2.5}$**	0.24 [-7.13, 7.61]	-	-1.67 [-9.14, 5.79]	2.15 [-4.32, 8.63]	1.35 [-7.46, 10.16]	2.59 [-5.97, 11.14]	-1.59 [-9.94, 6.77]
	**+PM_{Coarse}**	3.98 [-6.42, 14.38]	4.55 [-2.77, 11.88]	-	6.3 [-1.44, 14.04]	7.28 [-2.9, 17.46]	6.57 [-1.58, 14.73]	1.73 [-8.37, 11.84]
	**+PNC**	-3.35 [-14.22, 7.53]	2.04 [-5.85, 9.93]	-5.09 [-14.7, 4.52]	-	0.71 [-8.95, 10.36]	2.58 [-13.24, 18.4]	-3.89 [-13.84, 6.07]
	**+NO_2**	-1.08 [-10.84, 8.68]	2.69 [-6.2, 11.58]	-5.16 [-15.63, 5.31]	2.74 [-5.26, 10.74]	-	3.86 [-6.67, 14.39]	-6.53 [-19.69, 6.62]
	**+NO_X**	-2.22 [-11.81, 7.37]	1.52 [-7.92, 10.95]	-4.55 [-13.71, 4.61]	1.01 [-13.3, 15.32]	-0.31 [-11.81, 11.2]	-	-3.89 [-13.71, 5.93]
	**+$PM_{25}abs$**	2.85 [-7.5, 13.2]	4.57 [-3.22, 12.36]	-0.42 [-10.03, 9.18]	5.42 [-2.21, 13.04]	8.49 [-3.67, 20.65]	6.06 [-2.25, 14.37]	-
**Stroke volume**	**+PM_{10}**	-	0.97 [-2.68, 4.62]	0.72 [-4.53, 5.97]	-0.33 [-4.76, 4.11]	1.02 [-3.77, 5.81]	0.56 [-3.75, 4.88]	-3.12 [-8.61, 2.36]
	**+$PM_{2.5}$**	-0.13 [-3.85, 3.6]	-	0.11 [-3.66, 3.88]	-0.52 [-3.79, 2.76]	0.14 [-4.32, 4.59]	-0.28 [-4.61, 4.05]	-2.27 [-6.49, 1.94]
	**+PM_{Coarse}**	-0.19 [-5.44, 5.06]	0.85 [-2.85, 4.55]	-	-0.48 [-4.4, 3.44]	0.74 [-4.41, 5.89]	0.28 [-3.85, 4.41]	-3 [-8.09, 2.09]
	**+PNC**	0.72 [-4.78, 6.22]	1.3 [-2.69, 5.29]	1.02 [-3.84, 5.89]	-	1.43 [-3.45, 6.32]	2.81 [-5.19, 10.8]	-2.22 [-7.25, 2.82]
	**+NO_2**	-0.41 [-5.34, 4.52]	0.81 [-3.68, 5.3]	-0.03 [-5.33, 5.26]	-0.77 [-4.81, 3.27]	-	-0.17 [-5.5, 5.15]	-5.94 [-12.56, 0.69]
	**+NO_X**	-0.09 [-4.94, 4.76]	1.14 [-3.63, 5.91]	0.35 [-4.29, 4.99]	-2.23 [-9.46, 5.01]	0.88 [-4.94, 6.69]	-	-2.81 [-7.77, 2.15]

	**+PM25abs**	2.74 [-2.47, 7.96]	2.2 [-1.73, 6.13]	2.72 [-2.11, 7.56]	1.41 [-2.44, 5.27]	5.46 [-0.67, 11.58]	2.26 [-1.94, 6.46]	-
**Ejection fraction**	**+PM_{10}**	-	-0.52 [-2.55, 1.51]	1.22 [-1.69, 4.14]	-0.88 [-3.34, 1.58]	-0.65 [-3.32, 2.01]	-0.89 [-3.29, 1.5]	-0.74 [-3.79, 2.31]
	**+$PM_{2.5}$**	0.19 [-1.88, 2.26]	-	0.86 [-1.23, 2.96]	-0.22 [-2.04, 1.6]	-0.07 [-2.54, 2.41]	-0.35 [-2.76, 2.05]	-0.11 [-2.46, 2.24]
	**+PM_{Coarse}**	-1.06 [-3.98, 1.86]	-0.88 [-2.94, 1.18]	-	-1.36 [-3.54, 0.81]	-1.75 [-4.61, 1.11]	-1.46 [-3.76, 0.83]	-1.5 [-4.34, 1.33]
	**+PNC**	0.81 [-2.25, 3.86]	-0.26 [-2.48, 1.96]	1.66 [-1.04, 4.36]	-	-0.02 [-2.73, 2.7]	-0.63 [-5.08, 3.81]	-0.01 [-2.81, 2.79]
	**+NO_2**	0.43 [-2.32, 3.17]	-0.38 [-2.88, 2.12]	1.82 [-1.12, 4.76]	-0.34 [-2.59, 1.91]	-	-0.65 [-3.6, 2.31]	-0.13 [-3.83, 3.57]
	**+NO_X**	0.67 [-2.02, 3.36]	-0.13 [-2.79, 2.52]	1.58 [-1, 4.15]	0.18 [-3.84, 4.2]	0.26 [-2.97, 3.49]	-	0.09 [-2.67, 2.85]
	**+PM25abs**	0.47 [-2.43, 3.37]	-0.36 [-2.55, 1.82]	1.46 [-1.23, 4.15]	-0.35 [-2.49, 1.8]	-0.23 [-3.65, 3.19]	-0.5 [-2.84, 1.84]	-
**Diastolic myocardial mass**	**+PM_{10}**	-	-0.47 [-3.44, 2.49]	0.26 [-4.01, 4.52]	-1.84 [-5.43, 1.75]	-3.79 [-7.66, 0.08]	-2.27 [-5.76, 1.23]	-7.29 [-11.68, -2.89]**
	**+$PM_{2.5}$**	0.58 [-2.45, 3.6]	-	0.64 [-2.43, 3.7]	-0.5 [-3.16, 2.16]	-2.5 [-6.11, 1.11]	-1.48 [-4.99, 2.03]	-3.68 [-7.09, -0.28]**
	**+PM_{Coarse}**	0.12 [-4.14, 4.39]	-0.51 [-3.52, 2.49]	-	-1.44 [-4.62, 1.74]	-4.47 [-8.62, -0.31]**	-2.1 [-5.44, 1.25]	-6.3 [-10.39, -2.21]**
	**+PNC**	2.19 [-2.27, 6.65]	0.2 [-3.04, 3.44]	1.71 [-2.24, 5.66]	-	-2.4 [-6.35, 1.56]	-2.73 [-9.22, 3.76]	-4.62 [-8.69, -0.56]**
	**+NO_2**	3.3 [-0.68, 7.29]	1.64 [-2, 5.28]	4.03 [-0.25, 8.3]	1.13 [-2.14, 4.41]	-	0.99 [-3.33, 5.3]	-4.68 [-10.05, 0.7]
	**+NO_X**	2.25 [-1.68, 6.18]	1.05 [-2.82, 4.93]	2.07 [-1.69, 5.83]	1.91 [-3.96, 7.79]	-2.25 [-6.97, 2.47]	-	-3.91 [-7.92, 0.11]
	**+PM25abs**	5.82 [1.64, 10]**	1.91 [-1.27, 5.09]	4.87 [0.98, 8.75]**	2.24 [-0.87, 5.35]	2.4 [-2.57, 7.37]	1.68 [-1.71, 5.08]	-
**Global segments**	**+PM_{10}**	-	-1 [-3.16, 1.16]	1.03 [-2.07, 4.14]	-2.14 [-4.75, 0.47]	-3.73 [-6.54, -0.92]**	-2.79 [-5.33, -0.26]**	-3.9 [-7.13, -0.68]**
	**+$PM_{2.5}$**	0.05 [-2.15, 2.25]	-	0.75 [-1.48, 2.98]	-0.87 [-2.8, 1.06]	-2.52 [-5.14, 0.1]	-2 [-4.55, 0.55]	-1.93 [-4.42, 0.56]
	**+PM_{Coarse}**	-1.3 [-4.41, 1.81]	-1.37 [-3.56, 0.82]	-	-2.41 [-4.71, -0.1]**	-5.29 [-8.29, -2.29]**	-3.24 [-5.66, -0.82]**	-4.29 [-7.27, -1.3]**
	**+PNC**	1.69 [-1.56, 4.93]	-0.32 [-2.67, 2.04]	2.26 [-0.61, 5.12]	-	-2.36 [-5.23, 0.51]	-3.67 [-8.38, 1.04]	-1.91 [-4.88, 1.06]
	**+NO_2**	2.45 [-0.44, 5.34]	0.85 [-1.79, 3.49]	4.34 [1.26, 7.43]**	0.48 [-1.9, 2.86]	-	-0.16 [-3.29, 2.97]	-0.25 [-4.17, 3.67]
	**+NO_X**	1.89 [-0.96, 4.74]	0.69 [-2.12, 3.5]	2.64 [-0.08, 5.36]	2.07 [-2.19, 6.33]	-1.77 [-5.19, 1.66]	-	-1.14 [-4.07, 1.79]
	**+PM25abs**	2.46 [-0.61, 5.53]	0.12 [-2.2, 2.44]	3.07 [0.23, 5.91]**	0.06 [-2.21, 2.33]	-1.71 [-5.33, 1.91]	-0.82 [-3.29, 1.66]	-
**Basal segments**	**+PM_{10}**	-	-1.08 [-3.49, 1.34]	0.93 [-2.55, 4.4]	-2.46 [-5.38, 0.46]	-3.74 [-6.89, -0.59]**	-3.27 [-6.1, -0.43]**	-3.03 [-6.65, 0.6]
	**+$PM_{2.5}$**	-0.72 [-3.19, 1.74]	-	0.15 [-2.35, 2.65]	-1.52 [-3.68, 0.64]	-3.08 [-6.01, -0.15]**	-3 [-5.85, -0.15]**	-2 [-4.79, 0.79]

	**+PM$_{Coarse}$**	-2.03 [-5.51, 1.45]	-1.52 [-3.97, 0.93]	-	-2.93 [-5.51, -0.35]**	-5.5 [-8.87, -2.13]**	-3.89 [-6.6, -1.18]**	-3.84 [-7.2, -0.49]**
	**+PNC**	1.2 [-2.43, 4.83]	-0.29 [-2.92, 2.34]	2.07 [-1.14, 5.27]	-	-2.38 [-5.6, 0.83]	-4.37 [-9.63, 0.89]	-1.29 [-4.61, 2.03]
	**+NO$_2$**	1.65 [-1.59, 4.88]	0.79 [-2.16, 3.75]	3.83 [0.37, 7.29]**	-0.15 [-2.81, 2.51]	-	-1.14 [-4.64, 2.36]	0.61 [-3.77, 4.98]
	**+NO$_X$**	1.48 [-1.71, 4.66]	1.05 [-2.08, 4.19]	2.49 [-0.55, 5.54]	2.02 [-2.74, 6.78]	-1.46 [-5.29, 2.36]	-	-0.47 [-3.74, 2.8]
	**+PM$_{25}$abs**	0.99 [-2.46, 4.43]	-0.31 [-2.91, 2.29]	2.07 [-1.12, 5.26]	-0.93 [-3.48, 1.61]	-3 [-7.05, 1.04]	-1.98 [-4.75, 0.79]	-
**Mid segments**	**+PM$_{10}$**	-	-1.25 [-3.8, 1.29]	0.91 [-2.75, 4.56]	-2.37 [-5.45, 0.7]	-4.68 [-7.98, -1.38]**	-3.16 [-6.15, -0.18]**	-5.72 [-9.5, -1.94]**
	**+PM$_{2.5}$**	0.47 [-2.12, 3.06]	-	1.03 [-1.59, 3.66]	-0.66 [-2.93, 1.62]	-2.84 [-5.93, 0.24]	-1.86 [-4.87, 1.14]	-2.58 [-5.51, 0.34]
	**+PM$_{Coarse}$**	-0.92 [-4.57, 2.74]	-1.56 [-4.13, 1.01]	-	-2.41 [-5.13, 0.31]	-6.19 [-9.72, -2.66]**	-3.42 [-6.27, -0.57]**	-5.63 [-9.13, -2.13]**
	**+PNC**	2.21 [-1.61, 6.03]	-0.52 [-3.29, 2.26]	2.44 [-0.93, 5.82]	-	-3.07 [-6.45, 0.31]	-4.32 [-9.86, 1.23]	-3.14 [-6.63, 0.35]
	**+NO$_2$**	3.48 [0.09, 6.88]**	1.05 [-2.07, 4.16]	5.27 [1.64, 8.89]**	1.04 [-1.75, 3.84]	-	0.46 [-3.23, 4.15]	-1.29 [-5.9, 3.31]
	**+NO$_X$**	2.49 [-0.87, 5.85]	0.54 [-2.77, 3.85]	2.98 [-0.23, 6.18]	2.73 [-2.29, 7.75]	-2.52 [-6.55, 1.51]	-	-2.16 [-5.6, 1.28]
	**+PM$_{25}$abs**	4.12 [0.53, 7.71]**	0.45 [-2.28, 3.18]	4.22 [0.89, 7.54]**	0.87 [-1.8, 3.54]	-1.06 [-5.32, 3.2]	-0.14 [-3.06, 2.77]	-
**Apical segments**	**+PM$_{10}$**	-	-0.44 [-3.24, 2.36]	1.42 [-2.59, 5.44]	-1.17 [-4.56, 2.22]	-2.08 [-5.74, 1.59]	-1.32 [-4.62, 1.99]	-2.33 [-6.54, 1.87]
	**+PM$_{2.5}$**	0.7 [-2.16, 3.55]	-	1.32 [-1.57, 4.21]	-0.07 [-2.58, 2.44]	-0.96 [-4.37, 2.46]	-0.46 [-3.78, 2.85]	-0.68 [-3.91, 2.56]
	**+PM$_{Coarse}$**	-0.67 [-4.69, 3.35]	-0.78 [-3.61, 2.06]	-	-1.47 [-4.47, 1.52]	-3.36 [-7.29, 0.56]	-1.77 [-4.93, 1.39]	-2.75 [-6.65, 1.14]
	**+PNC**	1.65 [-2.56, 5.87]	-0.03 [-3.08, 3.03]	2.28 [-1.44, 6]	-	-1.09 [-4.83, 2.65]	-1.31 [-7.44, 4.81]	-0.89 [-4.75, 2.97]
	**+NO$_2$**	2.1 [-1.67, 5.87]	0.61 [-2.83, 4.05]	3.66 [-0.38, 7.7]	0.61 [-2.48, 3.71]	-	0.52 [-3.55, 4.6]	0.03 [-5.07, 5.13]
	**+NO$_X$**	1.58 [-2.13, 5.29]	0.3 [-3.35, 3.96]	2.34 [-1.21, 5.89]	1.03 [-4.52, 6.57]	-1.01 [-5.46, 3.45]	-	-0.57 [-4.37, 3.24]
	**+PM$_{25}$abs**	2.23 [-1.77, 6.22]	0.3 [-2.72, 3.32]	2.87 [-0.83, 6.57]	0.42 [-2.53, 3.38]	-0.54 [-5.26, 4.17]	0.1 [-3.12, 3.32]	-
**Lateral segments**	**+PM$_{10}$**	-	-0.66 [-2.96, 1.63]	0.31 [-2.99, 3.6]	-1.39 [-4.17, 1.39]	-3.52 [-6.51, -0.53]**	-2.19 [-4.89, 0.51]	-4.02 [-7.44, -0.59]**
	**+PM$_{2.5}$**	-0.38 [-2.72, 1.96]	-	0 [-2.37, 2.37]	-0.82 [-2.88, 1.23]	-2.89 [-5.67, -0.11]**	-1.97 [-4.68, 0.74]	-2.45 [-5.09, 0.19]
	**+PM$_{Coarse}$**	-0.97 [-4.27, 2.33]	-0.85 [-3.18, 1.47]	-	-1.54 [-4, 0.92]	-4.55 [-7.76, -1.35]**	-2.4 [-4.98, 0.18]	-4.01 [-7.19, -0.83]**
	**+PNC**	0.68 [-2.78, 4.13]	-0.23 [-2.73, 2.27]	0.98 [-2.07, 4.03]	-	-2.8 [-5.85, 0.25]	-3.65 [-8.66, 1.35]	-2.67 [-5.82, 0.48]
	**+NO$_2$**	2.03 [-1.04, 5.11]	1.24 [-1.56, 4.05]	3.28 [-0.02, 6.57]	0.85 [-1.67, 3.38]	-	0.44 [-2.89, 3.76]	-0.88 [-5.04, 3.28]
	**+NO$_X$**	1.13 [-1.91, 4.16]	0.79 [-2.2, 3.78]	1.5 [-1.41, 4.4]	2.15 [-2.38, 6.68]	-2.41 [-6.05, 1.23]	-	-1.87 [-4.98, 1.24]
	**+PM$_{25}$abs**	2.3 [-0.96, 5.56]	0.54 [-1.93, 3]	2.41 [-0.61, 5.43]	0.58 [-1.83, 3]	-1.3 [-5.15, 2.54]	-0.26 [-2.89, 2.37]	-

**Inferior segments**	**+PM_{10}**	-	-0.61 [-2.92, 1.71]	1.93 [-1.39, 5.25]	-1.21 [-4.02, 1.6]	-4.02 [-7.03, -1.01]**	-2.27 [-5, 0.45]	-5.35 [-8.79, -1.91]**
	**+$PM_{2.5}$**	-0.63 [-3, 1.73]	-	0.64 [-1.75, 3.04]	-0.91 [-2.98, 1.16]	-3.56 [-6.36, -0.76]**	-2.29 [-5.03, 0.44]	-3.46 [-6.12, -0.81]**
	**+PM_{Coarse}**	-2.49 [-5.81, 0.84]	-1.27 [-3.62, 1.08]	-	-2.34 [-4.82, 0.14]	-6.49 [-9.69, -3.29]**	-3.37 [-5.97, -0.78]**	-6.36 [-9.53, -3.2]**
	**+PNC**	0.27 [-3.22, 3.75]	-0.24 [-2.77, 2.29]	2.15 [-0.93, 5.22]	-	-3.53 [-6.6, -0.46]**	-4.41 [-9.46, 0.63]	-4.02 [-7.19, -0.86]**
	**+NO_2**	2.2 [-0.9, 5.29]	1.66 [-1.17, 4.48]	5.28 [2, 8.57]**	1.23 [-1.31, 3.77]	-	0.8 [-2.55, 4.15]	-2.2 [-6.38, 1.99]
	**+NO_X**	0.97 [-2.09, 4.03]	0.98 [-2.03, 4]	2.71 [-0.21, 5.62]	2.7 [-1.86, 7.27]	-3.13 [-6.79, 0.54]	-	-3.02 [-6.14, 0.11]
	**+PM_{25}abs**	3.08 [-0.19, 6.35]	1.04 [-1.44, 3.51]	4.51 [1.5, 7.52]**	1.27 [-1.15, 3.69]	-0.63 [-4.5, 3.24]	0.27 [-2.38, 2.91]	-
**Anterior segments**	**+PM_{10}**	-	-0.89 [-3.75, 1.97]	0.21 [-3.89, 4.32]	-2.92 [-6.37, 0.54]	-4.76 [-8.48, -1.04]**	-3.66 [-7.01, -0.3]**	-3.71 [-7.99, 0.57]
	**+$PM_{2.5}$**	0.88 [-2.04, 3.79]	-	0.9 [-2.05, 3.86]	-0.79 [-3.35, 1.77]	-2.83 [-6.3, 0.64]	-2.34 [-5.71, 1.04]	-1.19 [-4.49, 2.11]
	**+PM_{Coarse}**	0.24 [-3.87, 4.34]	-0.92 [-3.82, 1.98]	-	-2.23 [-5.29, 0.83]	-5.53 [-9.51, -1.54]**	-3.32 [-6.53, -0.11]**	-3.15 [-7.12, 0.83]
	**+PNC**	3.36 [-0.93, 7.65]	0.15 [-2.96, 3.27]	2.5 [-1.3, 6.29]	-	-2.54 [-6.35, 1.27]	-4.55 [-10.78, 1.69]	-0.78 [-4.72, 3.15]
	**+NO_2**	4.14 [0.32, 7.96]**	1.61 [-1.89, 5.11]	4.94 [0.84, 9.04]**	0.91 [-2.24, 4.07]	-	0 [-4.15, 4.16]	2.11 [-3.08, 7.3]
	**+NO_X**	3.5 [-0.26, 7.27]	1.5 [-2.22, 5.23]	3.12 [-0.49, 6.72]	3.14 [-2.51, 8.78]	-1.68 [-6.23, 2.86]	-	0.22 [-3.66, 4.1]
	**+PM_{25}abs**	3.2 [-0.87, 7.27]	0.23 [-2.84, 3.31]	2.66 [-1.12, 6.44]	-0.26 [-3.28, 2.75]	-3.37 [-8.17, 1.43]	-1.44 [-4.73, 1.84]	-
**Septal segments**	**+PM_{10}**	-	-1.84 [-4.21, 0.54]	1.8 [-1.61, 5.22]	-3.46 [-6.33, -0.6]**	-3.84 [-6.94, -0.74]**	-3.74 [-6.52, -0.95]**	-3.25 [-6.82, 0.31]
	**+$PM_{2.5}$**	0.15 [-2.27, 2.58]	-	1.37 [-1.08, 3.82]	-1.3 [-3.42, 0.82]	-1.98 [-4.86, 0.91]	-2.23 [-5.03, 0.58]	-1.18 [-3.92, 1.56]
	**+PM_{Coarse}**	-2.25 [-5.67, 1.17]	-2.48 [-4.88, -0.08]**	-	-3.98 [-6.5, -1.46]**	-6.14 [-9.44, -2.84]**	-4.61 [-7.26, -1.96]**	-4.4 [-7.7, -1.11]**
	**+PNC**	2.69 [-0.87, 6.25]	-0.77 [-3.36, 1.81]	3.75 [0.62, 6.88]**	-	-1.55 [-4.72, 1.61]	-3.25 [-8.43, 1.93]	-0.53 [-3.8, 2.74]
	**+NO_2**	2.2 [-0.99, 5.38]	-0.33 [-3.24, 2.59]	5.06 [1.67, 8.45]**	-0.7 [-3.32, 1.92]	-	-1.59 [-5.04, 1.86]	0.69 [-3.62, 5]
	**+NO_X**	2.35 [-0.78, 5.48]	0.1 [-2.99, 3.19]	3.78 [0.8, 6.76]**	1.06 [-3.63, 5.74]	-0.74 [-4.5, 3.03]	-	-0.01 [-3.22, 3.21]
	**+PM_{25}abs**	1.63 [-1.76, 5.02]	-1.09 [-3.65, 1.47]	3.17 [0.04, 6.3]**	-1.39 [-3.89, 1.11]	-2.76 [-6.74, 1.23]	-2.16 [-4.88, 0.57]	-
**RIGHT VENTRICLE**								
	**+PM_{10}**	-	1.63 [-1.91, 5.18]	-2.11 [-7.29, 3.07]	-0.24 [-4.68, 4.2]	1.31 [-3.45, 6.08]	1.02 [-3.27, 5.31]	-4.47 [-9.93, 1]
	**+$PM_{2.5}$**	-2.08 [-5.61, 1.45]	-	-3.01 [-6.75, 0.73]	-2.01 [-5.26, 1.23]	-1.73 [-6.12, 2.67]	-1.97 [-6.3, 2.37]	-5.05 [-9.25, -0.85]**

**End-diastolic volume**	**+PM$_{Coarse}$**	0.35 [-4.64, 5.35]	2.15 [-1.48, 5.78]	-	0.4 [-3.51, 4.31]	2.74 [-2.36, 7.83]	1.6 [-2.52, 5.73]	-3 [-8.06, 2.06]
	**+PNC**	-1.02 [-6.34, 4.29]	2.08 [-1.82, 5.98]	-2.2 [-7.04, 2.64]	-	1.11 [-3.72, 5.95]	4.2 [-3.67, 12.08]	-4.23 [-9.3, 0.85]
	**+NO$_2$**	-2.26 [-7, 2.48]	1.82 [-2.58, 6.21]	-4.08 [-9.34, 1.18]	-1.66 [-5.68, 2.36]	-	-0.35 [-5.66, 4.96]	-9.67 [-16.37, -2.97]**
	**+NO$_X$**	-2.09 [-6.71, 2.54]	2.2 [-2.5, 6.89]	-3.13 [-7.73, 1.48]	-4.48 [-11.58, 2.62]	-0.1 [-5.85, 5.64]	-	-5.35 [-10.33, -0.36]**
	**+PM$_{25}$abs**	2 [-3.02, 7.01]	3.46 [-0.41, 7.32]	0.33 [-4.48, 5.14]	1.51 [-2.38, 5.4]	7.34 [1.17, 13.51]**	2.97 [-1.27, 7.21]	-
**End-systolic volume**	**+PM$_{10}$**	-	0.81 [-4.07, 5.7]	-3.55 [-10.68, 3.58]	0.34 [-5.78, 6.46]	-0.31 [-6.88, 6.25]	0.83 [-5.07, 6.74]	-5.72 [-13.25, 1.81]
	**+PM$_{2.5}$**	-2.56 [-7.42, 2.31]	-	-3.97 [-9.12, 1.19]	-1.8 [-6.28, 2.68]	-2.95 [-9, 3.1]	-1.87 [-7.85, 4.1]	-5.89 [-11.69, -0.09]**
	**+PM$_{Coarse}$**	0.57 [-6.31, 7.44]	1.59 [-3.41, 6.59]	-	1.24 [-4.14, 6.62]	1.69 [-5.33, 8.72]	1.87 [-3.81, 7.54]	-3.52 [-10.49, 3.45]
	**+PNC**	-2.48 [-9.8, 4.83]	0.86 [-4.52, 6.24]	-4.25 [-10.92, 2.41]	-	-1.3 [-7.95, 5.36]	2.11 [-8.75, 12.97]	-6.15 [-13.13, 0.84]
	**+NO$_2$**	-1.91 [-8.44, 4.62]	1.63 [-4.42, 7.68]	-4.49 [-11.73, 2.76]	-0.53 [-6.07, 5.02]	-	0.9 [-6.4, 8.21]	-9.45 [-18.73, -0.18]**
	**+NO$_X$**	-2.82 [-9.19, 3.54]	1.06 [-5.42, 7.53]	-4.61 [-10.95, 1.73]	-3.13 [-12.92, 6.66]	-2.61 [-10.52, 5.3]	-	-6.74 [-13.61, 0.13]
	**+PM$_{25}$abs**	2.02 [-4.89, 8.93]	2.87 [-2.47, 8.22]	-0.56 [-7.19, 6.06]	2.21 [-3.15, 7.57]	5.81 [-2.74, 14.36]	3.16 [-2.69, 9.01]	-
**Stroke volume**	**+PM$_{10}$**	-	2.38 [-1.26, 6.01]	-0.87 [-6.19, 4.45]	-0.84 [-5.39, 3.72]	2.86 [-2.02, 7.74]	1.17 [-3.23, 5.57]	-3.25 [-8.87, 2.37]
	**+PM$_{2.5}$**	-1.67 [-5.29, 1.94]	-	-2.2 [-6.04, 1.63]	-2.26 [-5.58, 1.06]	-0.57 [-5.07, 3.93]	-2.1 [-6.54, 2.34]	-4.26 [-8.57, 0.05]
	**+PM$_{Coarse}$**	0.18 [-4.95, 5.31]	2.68 [-1.04, 6.4]	-	-0.39 [-4.4, 3.62]	3.81 [-1.42, 9.03]	1.37 [-2.87, 5.6]	-2.42 [-7.62, 2.78]
	**+PNC**	0.34 [-5.11, 5.79]	3.21 [-0.78, 7.2]	-0.35 [-5.32, 4.62]	-	3.42 [-1.52, 8.36]	6.21 [-1.84, 14.27]	-2.38 [-7.59, 2.84]
	**+NO$_2$**	-2.66 [-7.52, 2.19]	1.94 [-2.56, 6.44]	-3.86 [-9.25, 1.53]	-2.81 [-6.92, 1.31]	-	-1.62 [-7.05, 3.82]	-9.88 [-16.74, -3.02]**
	**+NO$_X$**	-1.43 [-6.17, 3.31]	3.25 [-1.55, 8.06]	-1.83 [-6.56, 2.9]	-5.83 [-13.1, 1.43]	2.3 [-3.59, 8.18]	-	-4.01 [-9.14, 1.12]
	**+PM$_{25}$abs**	1.89 [-3.27, 7.05]	3.96 [-0.02, 7.93]	1.02 [-3.92, 5.97]	0.77 [-3.23, 4.78]	8.75 [2.42, 15.07]**	2.73 [-1.64, 7.09]	-
**Ejection fraction**	**+PM$_{10}$**	-	0.91 [-1.32, 3.15]	1.36 [-1.91, 4.62]	-0.7 [-3.5, 2.1]	1.71 [-1.29, 4.71]	0.01 [-2.69, 2.72]	1.19 [-2.27, 4.64]
	**+PM$_{2.5}$**	0.5 [-1.73, 2.72]	-	0.91 [-1.46, 3.27]	-0.29 [-2.33, 1.76]	1.25 [-1.52, 4.01]	-0.33 [-3.07, 2.4]	0.78 [-1.88, 3.45]
	**+PM$_{Coarse}$**	-0.08 [-3.23, 3.07]	0.69 [-1.6, 2.98]	-	-0.86 [-3.32, 1.61]	1.23 [-1.99, 4.44]	-0.36 [-2.96, 2.24]	0.56 [-2.64, 3.75]
	**+PNC**	1.64 [-1.71, 4.98]	1.38 [-1.08, 3.84]	2.09 [-0.96, 5.14]	-	2.6 [-0.43, 5.64]	1.81 [-3.16, 6.78]	1.98 [-1.22, 5.19]
	**+NO$_2$**	-0.34 [-3.33, 2.64]	0.27 [-2.49, 3.04]	0.28 [-3.04, 3.6]	-1.25 [-3.78, 1.28]	-	-1.59 [-4.92, 1.74]	-0.41 [-4.67, 3.86]

	+NO_X	0.94 [-1.97, 3.86]	1.44 [-1.52, 4.4]	1.58 [-1.32, 4.49]	-1.1 [-5.58, 3.39]	2.9 [-0.71, 6.5]	-	1.53 [-1.63, 4.69]
	+PM_{25}abs	0.09 [-3.08, 3.26]	0.72 [-1.74, 3.18]	0.89 [-2.15, 3.93]	-0.73 [-3.19, 1.73]	1.77 [-2.16, 5.7]	-0.3 [-2.99, 2.39]	-
**VESSELS**								
**Diameter ascending Aorta**	+PM_{10}	-	-0.31 [-2.14, 1.53]	-1.35 [-4.08, 1.37]	1.78 [-0.5, 4.05]	0.03 [-2.49, 2.55]	1.26 [-0.97, 3.49]	-0.28 [-3.14, 2.58]
	+$PM_{2.5}$	1.23 [-0.66, 3.11]	-	0.27 [-1.7, 2.23]	1.85 [0.17, 3.52]**	1.21 [-1.08, 3.5]	2.45 [0.23, 4.67]**	0.94 [-1.22, 3.11]
	+PM_{Coarse}	2.11 [-0.54, 4.76]	0.17 [-1.69, 2.02]	-	2.52 [0.52, 4.52]**	1.48 [-1.19, 4.14]	2.16 [0.06, 4.27]**	1.2 [-1.43, 3.83]
	+PNC	-0.7 [-3.49, 2.09]	-1.05 [-3.04, 0.94]	-2.02 [-4.54, 0.5]	-	-0.97 [-3.49, 1.56]	-0.56 [-4.56, 3.44]	-1.08 [-3.68, 1.52]
	+NO_2	1.04 [-1.48, 3.56]	-0.53 [-2.76, 1.7]	-0.84 [-3.57, 1.89]	1.91 [-0.15, 3.97]	-	2.19 [-0.51, 4.9]	0.17 [-3.2, 3.54]
	+NO_X	0.01 [-2.47, 2.48]	-1.7 [-4.09, 0.7]	-1.42 [-3.82, 0.98]	1.78 [-1.84, 5.41]	-1.22 [-4.22, 1.78]	-	-0.66 [-3.21, 1.9]
	+PM_{25}abs	1.27 [-1.41, 3.96]	-0.22 [-2.2, 1.76]	-0.51 [-3.05, 2.03]	1.93 [-0.07, 3.92]	0.69 [-2.47, 3.86]	1.68 [-0.48, 3.84]	-
**Maximum diameter infrarenal Aorta**	+PM_{10}	-	-0.6 [-2.49, 1.29]	-2.34 [-5.15, 0.46]	-0.73 [-3.08, 1.63]	-1.09 [-3.68, 1.51]	-0.65 [-2.95, 1.65]	-3.02 [-5.95, -0.09]**
	+$PM_{2.5}$	-0.11 [-2.05, 1.84]	-	-1.18 [-3.19, 0.84]	-0.3 [-2.03, 1.43]	-0.6 [-2.96, 1.76]	-0.26 [-2.55, 2.04]	-1.61 [-3.83, 0.61]
	+PM_{Coarse}	1.38 [-1.34, 4.11]	-0.07 [-1.98, 1.84]	-	0.47 [-1.6, 2.54]	0.44 [-2.29, 3.18]	0.42 [-1.75, 2.59]	-1.18 [-3.88, 1.52]
	+PNC	0.31 [-2.57, 3.19]	-0.43 [-2.49, 1.63]	-1.66 [-4.27, 0.95]	-	-0.68 [-3.29, 1.93]	-0.2 [-4.33, 3.94]	-2.16 [-4.84, 0.52]
	+NO_2	0.41 [-2.18, 3]	-0.24 [-2.54, 2.05]	-1.57 [-4.38, 1.23]	-0.1 [-2.23, 2.03]	-	0.07 [-2.72, 2.86]	-2.86 [-6.31, 0.6]
	+NO_X	0.13 [-2.43, 2.68]	-0.44 [-2.92, 2.04]	-1.57 [-4.04, 0.91]	-0.36 [-4.11, 3.39]	-0.84 [-3.94, 2.26]	-	-2.12 [-4.74, 0.51]
	+PM_{25}abs	1.84 [-0.91, 4.59]	0.23 [-1.8, 2.26]	-0.36 [-2.97, 2.24]	0.72 [-1.34, 2.77]	1.53 [-1.72, 4.77]	0.75 [-1.47, 2.97]	-
**Diameter pulmonary trunk**	+PM_{10}	-	-1.89 [-4.01, 0.23]	-1.16 [-4.32, 2.01]	1.23 [-1.42, 3.87]	-0.89 [-3.81, 2.03]	-0.03 [-2.61, 2.56]	-1.83 [-5.14, 1.48]
	+$PM_{2.5}$	1.48 [-0.7, 3.65]	-	0.83 [-1.44, 3.09]	2.14 [0.21, 4.07]**	1.66 [-0.98, 4.31]	2.61 [0.05, 5.18]**	0.78 [-1.72, 3.28]
	+PM_{Coarse}	1.38 [-1.69, 4.45]	-1.57 [-3.71, 0.58]	-	1.75 [-0.58, 4.08]	0.08 [-3, 3.16]	0.71 [-1.73, 3.16]	-0.53 [-3.57, 2.52]
	+PNC	-0.73 [-3.97, 2.5]	-2.72 [-5.01, -0.42]**	-1.69 [-4.62, 1.24]	-	-1.68 [-4.61, 1.25]	-3.29 [-7.92, 1.34]	-2.22 [-5.24, 0.79]
	+NO_2	1.17 [-1.74, 4.08]	-2.3 [-4.86, 0.27]	-0.1 [-3.26, 3.07]	1.79 [-0.6, 4.18]	-	1.09 [-2.04, 4.23]	-0.98 [-4.87, 2.92]
	+NO_X	0.51 [-2.36, 3.38]	-3.28 [-6.05, -0.52]**	-0.62 [-3.41, 2.17]	3.52 [-0.68, 7.71]	-1.02 [-4.51, 2.47]	-	-1.17 [-4.13, 1.79]
	+PM_{25}abs	1.86 [-1.25, 4.96]	-1.59 [-3.87, 0.7]	0.35 [-2.59, 3.29]	2.02 [-0.29, 4.33]	0.79 [-2.87, 4.45]	1.05 [-1.45, 3.55]	-
**Diameter right**	+PM_{10}	-	-0.32 [-2.69, 2.06]	0.19 [-3.34, 3.72]	0.53 [-2.43, 3.48]	0.71 [-2.55, 3.96]	0.39 [-2.5, 3.27]	0.27 [-3.43, 3.97]
	+$PM_{2.5}$	0.44 [-2, 2.88]	-	0.48 [-2.05, 3.01]	0.66 [-1.51, 2.83]	1.14 [-1.82, 4.1]	0.98 [-1.9, 3.86]	0.62 [-2.18, 3.41]
	+PM_{Coarse}	0.13 [-3.3, 3.56]	-0.33 [-2.73, 2.06]	-	0.43 [-2.18, 3.03]	0.75 [-2.69, 4.18]	0.35 [-2.38, 3.08]	0.24 [-3.16, 3.63]
	+PNC	-0.25 [-3.87, 3.36]	-0.58 [-3.17, 2.01]	-0.11 [-3.39, 3.17]	-	0.4 [-2.87, 3.68]	-0.13 [-5.32, 5.06]	-0.05 [-3.42, 3.33]

**pulmonary artery**	**+NO_2**	-0.27 [-3.52, 2.99]	-0.88 [-3.76, 1.99]	-0.31 [-3.84, 3.22]	0.11 [-2.56, 2.78]	-	-0.1 [-3.6, 3.4]	-0.49 [-4.84, 3.86]
	**+NO_X**	-0.05 [-3.25, 3.15]	-0.89 [-4, 2.21]	0.01 [-3.11, 3.12]	0.47 [-4.23, 5.17]	0.6 [-3.29, 4.49]	-	0.05 [-3.25, 3.36]
	**+$PM_{25}abs$**	0.07 [-3.4, 3.54]	-0.44 [-2.99, 2.12]	0.12 [-3.16, 3.4]	0.39 [-2.2, 2.98]	0.9 [-3.19, 4.98]	0.32 [-2.48, 3.12]	-
**Diameter left pulmonary artery**	**+PM_{10}**	-	-1.13 [-3.1, 0.84]	1.44 [-1.49, 4.37]	0.67 [-1.78, 3.12]	1.38 [-1.32, 4.08]	0.05 [-2.35, 2.45]	-0.36 [-3.43, 2.71]
	**+$PM_{2.5}$**	0.05 [-1.97, 2.08]	-	0.98 [-1.12, 3.07]	0.7 [-1.1, 2.5]	1.91 [-0.53, 4.36]	0.99 [-1.4, 3.37]	0.22 [-2.1, 2.53]
	**+PM_{Coarse}**	-1.64 [-4.49, 1.2]	-1.59 [-3.57, 0.4]	-	-0.44 [-2.61, 1.72]	0.23 [-2.62, 3.09]	-0.85 [-3.12, 1.42]	-1.53 [-4.35, 1.28]
	**+PNC**	-1.2 [-4.21, 1.8]	-1.61 [-3.76, 0.53]	0.52 [-2.2, 3.25]	-	0.7 [-2.02, 3.42]	-1.57 [-5.88, 2.73]	-1.04 [-3.84, 1.76]
	**+NO_2**	-1.59 [-4.29, 1.11]	-2.41 [-4.79, -0.04]**	-0.08 [-3.02, 2.85]	-0.57 [-2.79, 1.65]	-	-1.61 [-4.52, 1.3]	-2.78 [-6.39, 0.82]
	**+NO_X**	-0.58 [-3.24, 2.08]	-1.91 [-4.48, 0.67]	0.8 [-1.78, 3.39]	1.2 [-2.71, 5.1]	1.67 [-1.56, 4.9]	-	-0.62 [-3.37, 2.12]
	**+$PM_{25}abs$**	-0.27 [-3.15, 2.61]	-1.22 [-3.34, 0.89]	1.22 [-1.5, 3.93]	0.47 [-1.68, 2.62]	2.41 [-0.97, 5.79]	0.05 [-2.28, 2.37]	-

Table S19: Cardiovascular outcomes and long-term exposure to air pollution, two-pollutant models – percent change and confidence intervals of the outcome mean per interquartile range increase in the air pollutant of interest. Main model: adjusted for sex, height, body mass index, income, marital status, education years and smoking. Significant results with p-value <0.05 are marked with **.

Supplement Table S20 – Two-pollutant models and adipose tissue outcomes

Outcome	Second pollutant	PM_{10}	$PM_{2.5}$	PM_{Coarse}	PNC	NO_2	NO_X	$PM_{25}abs$
		%-change [95%-CI]	*%-change [95%-CI]*	*%-change [95%-CI]*	*%-change [95%-CI]*	*%-change [95%-CI]*	*%-change [95%-CI]*	*%-change [95%-CI]*
**Total AT**	**+PM_{10}**	-	0.94 [-2.16, 4.04]	4.04 [-0.47, 8.55]	1.82 [-2.01, 5.64]	-1.37 [-5.58, 2.85]	0.73 [-2.99, 4.45]	-0.41 [-5.24, 4.42]
	**+$PM_{2.5}$**	-0.61 [-3.76, 2.55]	-	1.48 [-1.77, 4.73]	0.34 [-2.49, 3.17]	-2.17 [-5.95, 1.61]	-0.47 [-4.2, 3.26]	-1 [-4.58, 2.59]
	**+PM_{Coarse}**	-3.25 [-7.67, 1.16]	-0.11 [-3.24, 3.03]	-	-0.72 [-4.1, 2.65]	-4.68 [-9.07, -0.29]**	-1.38 [-4.91, 2.15]	-3.09 [-7.45, 1.27]
	**+PNC**	-1.92 [-6.56, 2.73]	0.39 [-2.99, 3.77]	2.09 [-2.09, 6.28]	-	-2.83 [-7.06, 1.39]	-2.37 [-9.12, 4.38]	-1.73 [-6.08, 2.62]
	**+NO_2**	0.93 [-3.3, 5.16]	2.13 [-1.6, 5.87]	5.21 [0.71, 9.71]**	2.34 [-1.15, 5.83]	-	2.51 [-2.02, 7.04]	1.31 [-4.31, 6.93]
	**+NO_X**	-0.73 [-4.83, 3.37]	1.01 [-3.04, 5.07]	2.54 [-1.45, 6.52]	2.54 [-3.59, 8.67]	-2.98 [-7.96, 2]	-	-0.96 [-5.18, 3.26]
	**+$PM_{25}abs$**	0.18 [-4.33, 4.69]	1.17 [-2.12, 4.46]	3.62 [-0.54, 7.78]	1.53 [-1.81, 4.87]	-1.7 [-6.93, 3.52]	0.83 [-2.75, 4.4]	-
**Visceral AT**	**+PM_{10}**	-	2.25 [-3.72, 8.22]	6.16 [-2.54, 14.86]	4.74 [-2.61, 12.1]	3.34 [-4.77, 11.46]	3.44 [-3.71, 10.59]	2.74 [-6.55, 12.04]
	**+$PM_{2.5}$**	-0.86 [-6.93, 5.21]	-	2.16 [-4.1, 8.42]	1.42 [-4.02, 6.86]	0.63 [-6.66, 7.91]	1.04 [-6.14, 8.22]	0.15 [-6.75, 7.05]
	**+PM_{Coarse}**	-4.47 [-12.99, 4.06]	0.74 [-5.3, 6.77]	-	0.64 [-5.86, 7.13]	-0.97 [-9.48, 7.54]	0.18 [-6.64, 6.99]	-1.69 [-10.12, 6.74]
	**+PNC**	-4.38 [-13.32, 4.56]	0.78 [-5.72, 7.29]	1.96 [-6.09, 10.02]	-	-0.21 [-8.36, 7.94]	-1.16 [-14.15, 11.84]	-1.14 [-9.52, 7.24]
	**+NO_2**	-2.27 [-10.42, 5.87]	1.39 [-5.81, 8.59]	3.34 [-5.38, 12.06]	1.96 [-4.77, 8.68]	-	1.65 [-7.09, 10.38]	-0.84 [-11.65, 9.98]
	**+NO_X**	-2.54 [-10.43, 5.35]	0.97 [-6.83, 8.77]	2.41 [-5.26, 10.09]	2.8 [-9, 14.6]	0.09 [-9.51, 9.69]	-	-0.45 [-8.58, 7.68]
	**+$PM_{25}abs$**	-1.74 [-10.42, 6.94]	1.74 [-4.6, 8.08]	3.76 [-4.27, 11.79]	2.47 [-3.97, 8.91]	2.27 [-7.79, 12.33]	1.99 [-4.89, 8.86]	-
**Subcutaneous AT**	**+PM_{10}**	-	0.2 [-2.99, 3.39]	2.84 [-1.81, 7.49]	0.17 [-3.76, 4.1]	-4 [-8.31, 0.32]	-0.79 [-4.61, 3.03]	-2.17 [-7.13, 2.79]
	**+$PM_{2.5}$**	-0.46 [-3.71, 2.78]	-	1.09 [-2.25, 4.43]	-0.27 [-3.17, 2.64]	-3.72 [-7.59, 0.15]	-1.31 [-5.14, 2.53]	-1.63 [-5.31, 2.05]
	**+PM_{Coarse}**	-2.56 [-7.11, 1.99]	-0.58 [-3.8, 2.64]	-	-1.48 [-4.95, 1.98]	-6.73 [-11.22, -2.24]**	-2.24 [-5.87, 1.39]	-3.86 [-8.34, 0.63]
	**+PNC**	-0.53 [-5.31, 4.25]	0.16 [-3.31, 3.64]	2.15 [-2.15, 6.45]	-	-4.29 [-8.62, 0.04]	-3.03 [-9.97, 3.9]	-2.06 [-6.53, 2.41]
	**+NO_2**	2.72 [-1.62, 7.05]	2.54 [-1.29, 6.36]	6.23 [1.63, 10.84]**	2.54 [-1.04, 6.12]	-	2.98 [-1.66, 7.63]	2.5 [-3.25, 8.26]
	**+NO_X**	0.29 [-3.93, 4.5]	1.03 [-3.13, 5.2]	2.59 [-1.5, 6.68]	2.38 [-3.92, 8.68]	-4.68 [-9.78, 0.42]	-	-1.24 [-5.58, 3.09]
	**+$PM_{25}abs$**	1.25 [-3.38, 5.88]	0.84 [-2.54, 4.22]	3.52 [-0.75, 7.79]	0.99 [-2.44, 4.43]	-3.92 [-9.27, 1.44]	0.17 [-3.5, 3.84]	-

**Epi- and pericardial AT**	**+PM_{10}**	-	-7.11 [-15.15, 0.92]	0.44 [-11.17, 12.05]	-6.18 [-15.83, 3.47]	-4.08 [-14.83, 6.68]	-4.59 [-14.16, 4.98]	-2.43 [-14.74, 9.88]
	**+$PM_{2.5}$**	7.61 [-0.54, 15.76]	-	6.74 [-1.42, 14.91]	3.33 [-3.79, 10.46]	6.93 [-2.81, 16.68]	6.56 [-2.93, 16.04]	6.99 [-2.29, 16.26]
	**+PM_{Coarse}**	3.49 [-8.1, 15.07]	-6.65 [-14.68, 1.39]	-	-3.43 [-11.91, 5.04]	-3.66 [-15.16, 7.84]	-2.98 [-12, 6.04]	-0.51 [-11.88, 10.85]
	**+PNC**	10.12 [-1.92, 22.17]	-5.73 [-14.51, 3.04]	6.45 [-4.14, 17.05]	-	1.99 [-8.95, 12.93]	1.5 [-16.12, 19.12]	4.4 [-6.81, 15.61]
	**+NO_2**	6.98 [-3.85, 17.82]	-8.05 [-17.73, 1.64]	6.18 [-5.43, 17.79]	-0.8 [-9.63, 8.04]	-	-0.97 [-12.64, 10.7]	4.65 [-9.67, 18.97]
	**+NO_X**	7.73 [-2.96, 18.42]	-8.64 [-19.08, 1.81]	5.63 [-4.47, 15.73]	-0.81 [-16.58, 14.96]	2.13 [-10.81, 15.08]	-	3.91 [-7.03, 14.86]
	**+PM_{25}abs**	5.66 [-5.92, 17.24]	-7.09 [-15.7, 1.51]	3.6 [-7.11, 14.3]	-2.04 [-10.48, 6.41]	-2.49 [-15.86, 10.88]	-1.77 [-10.98, 7.45]	-
**Systolic epicardial AT**	**+PM_{10}**	-	-8.91 [-17.01, -0.8]**	-3.45 [-15.17, 8.28]	-2.54 [-12.32, 7.23]	-6.63 [-17.48, 4.23]	-2.3 [-11.99, 7.38]	-4.46 [-16.9, 7.97]
	**+$PM_{2.5}$**	6.2 [-2.02, 14.42]	-	4.05 [-4.19, 12.29]	4.87 [-2.3, 12.03]	4.82 [-5, 14.65]	9.09 [-0.43, 18.61]	5.25 [-4.1, 14.6]
	**+PM_{Coarse}**	4.22 [-7.48, 15.92]	-7.79 [-15.89, 0.32]	-	0.36 [-8.21, 8.93]	-4.33 [-15.95, 7.29]	0.14 [-8.98, 9.26]	-0.73 [-12.22, 10.75]
	**+PNC**	4.06 [-8.13, 16.26]	-9.43 [-18.24, -0.61]**	-0.41 [-11.12, 10.3]	-	-4.31 [-15.35, 6.72]	-0.77 [-18.55, 17.01]	-1.1 [-12.42, 10.22]
	**+NO_2**	6.58 [-4.35, 17.52]	-9.09 [-18.85, 0.67]	3.41 [-8.31, 15.14]	2.81 [-6.1, 11.71]	-	4.38 [-7.39, 16.14]	4.15 [-10.3, 18.61]
	**+NO_X**	3.43 [-7.39, 14.25]	-13.26 [-23.75, -2.77]**	-0.19 [-10.4, 10.03]	0.76 [-15.16, 16.68]	-5.73 [-18.77, 7.32]	-	-0.85 [-11.9, 10.21]
	**+PM_{25}abs**	4.83 [-6.87, 16.52]	-8.65 [-17.32, 0.03]	0.45 [-10.37, 11.27]	0.74 [-7.8, 9.27]	-4.94 [-18.43, 8.55]	0.54 [-8.76, 9.85]	-
**Diastolic epicardial AT**	**+PM_{10}**	-	-9.02 [-17.27, -0.77]**	-2.19 [-14.13, 9.75]	-4.17 [-14.11, 5.77]	-5.66 [-16.72, 5.39]	-4.15 [-14, 5.69]	-1.93 [-14.59, 10.74]
	**+$PM_{2.5}$**	6.02 [-2.34, 14.39]	-	4.56 [-3.82, 12.95]	3.9 [-3.4, 11.19]	5.55 [-4.45, 15.54]	7.22 [-2.49, 16.93]	6.59 [-2.91, 16.1]
	**+PM_{Coarse}**	2.98 [-8.93, 14.9]	-8.25 [-16.5, 0]	-	-1.61 [-10.33, 7.11]	-4.3 [-16.13, 7.52]	-2.19 [-11.47, 7.09]	0.45 [-11.23, 12.14]
	**+PNC**	5.49 [-6.92, 17.89]	-8.89 [-17.86, 0.09]	1.71 [-9.19, 12.62]	-	-2.1 [-13.34, 9.14]	-2.49 [-20.58, 15.6]	2.12 [-9.4, 13.64]
	**+NO_2**	5.61 [-5.53, 16.75]	-9.8 [-19.73, 0.13]	3.66 [-8.27, 15.6]	0.73 [-8.34, 9.8]	-	0.5 [-11.48, 12.49]	6.09 [-8.61, 20.79]
	**+NO_X**	4.76 [-6.24, 15.76]	-11.91 [-22.6, -1.21]**	1.97 [-8.42, 12.36]	1.5 [-14.7, 17.7]	-1.88 [-15.17, 11.41]	-	2.51 [-8.73, 13.76]

	+PM_{25}abs	2.69 [-9.23, 14.6]	-9.6 [-18.41, -0.78]**	-0.12 [-11.14, 10.89]	-1.78 [-10.46, 6.9]	-6.26 [-19.99, 7.46]	-2.48 [-11.95, 6.99]	-
**Pericardial systolic AT**	+PM_{10}	-	-5.58 [-13.44, 2.27]	-6.07 [-17.37, 5.24]	-1.84 [-11.28, 7.59]	-8.63 [-19.09, 1.83]	-2.34 [-11.69, 7.01]	-7.59 [-19.58, 4.4]
	+$PM_{2.5}$	7.55 [-0.42, 15.52]	-	3.24 [-4.77, 11.25]	5.26 [-1.69, 12.21]	2.2 [-7.35, 11.76]	7.59 [-1.67, 16.85]	3.51 [-5.58, 12.6]
	+PM_{Coarse}	9.42 [-1.86, 20.71]	-3.35 [-11.23, 4.53]	-	3.45 [-4.83, 11.73]	-3.45 [-14.69, 7.8]	2.45 [-6.37, 11.27]	-0.1 [-11.21, 11.01]
	+PNC	6.47 [-5.31, 18.24]	-5.71 [-14.26, 2.85]	-1.77 [-12.13, 8.59]	-	-5.9 [-16.55, 4.76]	-2.91 [-20.1, 14.27]	-2.81 [-13.75, 8.13]
	+NO_2	11.24 [0.71, 21.78]**	-3.23 [-12.73, 6.26]	4.25 [-7.11, 15.6]	6.06 [-2.54, 14.67]	-	7.62 [-3.75, 18.98]	4.53 [-9.46, 18.51]
	+NO_X	6.57 [-3.87, 17.01]	-7.99 [-18.19, 2.21]	-0.51 [-10.39, 9.36]	4.82 [-10.56, 20.21]	-7.22 [-19.83, 5.39]	-	-1.55 [-12.24, 9.14]
	+PM_{25}abs	10.29 [-0.99, 21.56]	-3.65 [-12.09, 4.78]	1.54 [-8.93, 12.01]	3.97 [-4.28, 12.21]	-3.7 [-16.76, 9.36]	3.08 [-5.92, 12.08]	-
**Pericardial diastolic AT**	+PM_{10}	-	-6.42 [-14.34, 1.5]	-5.04 [-16.46, 6.37]	-2.73 [-12.24, 6.79]	-9.25 [-19.79, 1.3]	-3.22 [-12.65, 6.2]	-7.51 [-19.6, 4.58]
	+$PM_{2.5}$	7.93 [-0.1, 15.96]	-	4.15 [-3.92, 12.22]	5.24 [-1.77, 12.25]	2.5 [-7.13, 12.14]	7.63 [-1.71, 16.97]	4.13 [-5.04, 13.29]
	+PM_{Coarse}	8.55 [-2.84, 19.94]	-4.45 [-12.39, 3.49]	-	2.23 [-6.12, 10.59]	-4.98 [-16.32, 6.35]	1.15 [-7.76, 10.05]	-0.76 [-11.97, 10.44]
	+PNC	7.31 [-4.57, 19.18]	-6.33 [-14.95, 2.3]	-0.31 [-10.76, 10.15]	-	-5.85 [-16.61, 4.9]	-3.46 [-20.79, 13.88]	-2.1 [-13.14, 8.94]
	+NO_2	11.66 [1.04, 22.28]**	-4.08 [-13.65, 5.49]	5.81 [-5.63, 17.25]	5.7 [-2.98, 14.38]	-	6.96 [-4.51, 18.43]	5.47 [-8.64, 19.58]
	+NO_X	7.26 [-3.27, 17.79]	-8.66 [-18.95, 1.63]	0.87 [-9.1, 10.83]	4.94 [-10.58, 20.46]	-6.91 [-19.63, 5.82]	-	-0.81 [-11.6, 9.98]
	+PM_{25}abs	10.17 [-1.21, 21.54]	-4.63 [-13.13, 3.87]	2.34 [-8.22, 12.89]	3.24 [-5.09, 11.56]	-4.76 [-17.93, 8.41]	2.2 [-6.88, 11.28]	-
**Mean AT content of the liver**	+PM_{10}	-	1.07 [-0.28, 2.43]	0.66 [-1.34, 2.67]	-0.13 [-1.86, 1.6]	0.73 [-1.12, 2.58]	0.23 [-1.42, 1.88]	0.81 [-1.3, 2.92]
	+$PM_{2.5}$	-0.42 [-1.78, 0.95]	-	-0.18 [-1.6, 1.25]	-0.63 [-1.86, 0.6]	-0.44 [-2.11, 1.23]	-0.95 [-2.58, 0.68]	-0.23 [-1.82, 1.35]
	+PM_{Coarse}	-0.39 [-2.35, 1.56]	0.96 [-0.42, 2.34]	-	-0.39 [-1.89, 1.11]	0.35 [-1.62, 2.32]	-0.08 [-1.65, 1.49]	0.31 [-1.65, 2.26]
	+PNC	0.25 [-1.84, 2.34]	1.33 [-0.15, 2.8]	0.71 [-1.15, 2.56]	-	0.87 [-0.99, 2.74]	1.07 [-1.91, 4.06]	0.82 [-1.1, 2.74]
	+NO_2	-0.43 [-2.28, 1.41]	1.17 [-0.48, 2.83]	0.06 [-1.95, 2.06]	-0.51 [-2.05, 1.02]	-	-0.41 [-2.41, 1.59]	0.15 [-2.34, 2.64]
	+NO_X	-0.07 [-1.87, 1.74]	1.64 [-0.13, 3.41]	0.41 [-1.36, 2.17]	-0.87 [-3.57, 1.84]	0.77 [-1.43, 2.97]	-	0.53 [-1.35, 2.4]
	+PM_{25}abs	-0.47 [-2.42, 1.47]	0.99 [-0.46, 2.45]	0.12 [-1.72, 1.97]	-0.43 [-1.89, 1.04]	0.27 [-2.04, 2.59]	-0.14 [-1.72, 1.44]	-
	+PM_{10}	-	1.22 [-0.32, 2.76]	0.75 [-1.53, 3.03]	-0.15 [-2.11, 1.82]	0.83 [-1.28, 2.94]	0.26 [-1.62, 2.14]	0.92 [-1.48, 3.33]

**Mean AT content of the pancreas**	**+$PM_{2.5}$**	-0.48 [-2.03, 1.08]	-	-0.2 [-1.82, 1.42]	-0.72 [-2.12, 0.68]	-0.5 [-2.41, 1.4]	-1.08 [-2.93, 0.78]	-0.27 [-2.07, 1.54]
	**+PM_{Coarse}**	-0.45 [-2.67, 1.78]	1.09 [-0.48, 2.66]	-	-0.45 [-2.15, 1.26]	0.4 [-1.84, 2.64]	-0.09 [-1.88, 1.7]	0.35 [-1.87, 2.57]
	**+PNC**	0.29 [-2.09, 2.66]	1.51 [-0.17, 3.19]	0.81 [-1.3, 2.91]	-	0.99 [-1.13, 3.11]	1.22 [-2.18, 4.62]	0.93 [-1.26, 3.12]
	**+NO_2**	-0.49 [-2.59, 1.6]	1.34 [-0.54, 3.22]	0.07 [-2.22, 2.35]	-0.58 [-2.33, 1.16]	-	-0.46 [-2.74, 1.81]	0.17 [-2.66, 3.01]
	**+NO_X**	-0.08 [-2.13, 1.98]	1.87 [-0.15, 3.88]	0.46 [-1.55, 2.47]	-0.99 [-4.07, 2.09]	0.88 [-1.63, 3.39]	-	0.6 [-1.53, 2.73]
	**+PM_{25}abs**	-0.54 [-2.76, 1.68]	1.13 [-0.52, 2.79]	0.14 [-1.96, 2.24]	-0.48 [-2.16, 1.19]	0.31 [-2.32, 2.94]	-0.16 [-1.96, 1.64]	-
**Renal hilus AT**	**+PM_{10}**	-	5.76 [-0.39, 11.91]	6.32 [-2.92, 15.57]	9.89 [2.3, 17.49]**	2.8 [-5.62, 11.21]	7.44 [0.07, 14.81]**	0.71 [-8.85, 10.26]
	**+$PM_{2.5}$**	-2.78 [-9.16, 3.6]	-	0.18 [-6.46, 6.82]	2.05 [-3.64, 7.73]	-3.62 [-11.31, 4.06]	0.88 [-6.65, 8.42]	-3.8 [-11.05, 3.45]
	**+PM_{Coarse}**	-4.51 [-13.48, 4.46]	4.26 [-1.94, 10.47]	-	4.62 [-2.12, 11.35]	-1.51 [-10.32, 7.3]	3.75 [-3.22, 10.72]	-3.58 [-12.47, 5.31]
	**+PNC**	-9.58 [-18.93, -0.23]**	2.82 [-3.92, 9.56]	-1.8 [-10.34, 6.75]	-	-4.7 [-13.14, 3.74]	-1.38 [-14.77, 12.02]	-6.59 [-15.22, 2.03]
	**+NO_2**	-1.8 [-10.3, 6.7]	6.87 [-0.6, 14.35]	3.87 [-5.31, 13.05]	6.52 [-0.4, 13.44]	-	8.15 [-0.84, 17.14]	-2.94 [-14.33, 8.44]
	**+NO_X**	-5.92 [-14.18, 2.34]	3.62 [-4.51, 11.76]	-0.48 [-8.54, 7.57]	4.71 [-7.48, 16.91]	-6.19 [-16.16, 3.78]	-	-5.37 [-13.84, 3.1]
	**+PM_{25}abs**	-0.17 [-9.24, 8.91]	6.49 [-0.14, 13.12]	5.31 [-3.4, 14.01]	7.36 [0.71, 14.01]**	3.82 [-6.88, 14.52]	6.81 [-0.37, 13.98]	-

Table S20: Adipose tissue outcomes and long-term exposure to air pollution, two-pollutant models – percent change and confidence intervals of the outcome mean per interquartile range increase in the air pollutant of interest. Main model: adjusted for age, sex, height, income, and physical activity. Significant results with p-value <0.05 are marked with **.
